# Membranes for Lithium Recovery From Conventional and Unconventional Sources

**DOI:** 10.1021/acsestengg.5c00997

**Published:** 2026-04-07

**Authors:** Nurshaun Sreedhar, Rebecca Lee, Sreejith Appukuttan, Hariswaran Sitaraman, Jason DesVeaux, Gary Grim, Mou Paul, Manish Kumar, Abhishek Roy

**Affiliations:** 1 Maseeh Department of Civil, Architectural and Environmental Engineering, 12330 The University of Texas at Austin, Austin, Texas 78712, United States; 2 National Laboratory of the Rockies, Golden, Colorado 80401, United States; 3 McKetta Department of Chemical Engineering, 12330 The University of Texas at Austin, Austin, Texas 78712, United States

**Keywords:** Lithium recovery, Membrane technologies, Direct lithium extraction (DLE), Selective ion separation, Critical mineral recovery

## Abstract

Lithium has been deemed a critical mineral of national importance that finds uses in a wide range of applications, and its demand has been rising significantly in recent years. The urgency of meeting this demand requires lithium extraction from various aqueous sources such as continental brines, geothermal brines, seawater, produced water, and battery waste. While direct lithium extraction (DLE) technologies such as adsorption, ion exchange, and solvent extraction have emerged as possible solutions, membrane technologies are also being investigated for various sources and at different stages of the recovery process. Here, we analyze the application of membranes for pretreatment of lithium source waters, bring management, lithium/magnesium separation, lithium/sodium separation, and lithium hydroxide conversion, and evaluate performance metrics for critical lithium separations from the literature. We explore the potential of membranes at every stage of the recovery process and describe their current status and future prospects. We describe hypothetical process trains with integrated membrane technologies for each source type and address their feasibility and challenges. The potential energy and water impacts of membrane-integrated and conventional DLE processes are also critically considered alongside performance and selectivity metrics, and this is illustrated using examples and calculated from published technical reports. This paper thus provides a comprehensive overview of the application of membranes along every stage of the lithium recovery process, emphasizing the versatility and potential of membrane technologies for critical mineral recovery.

## Graphical abstract

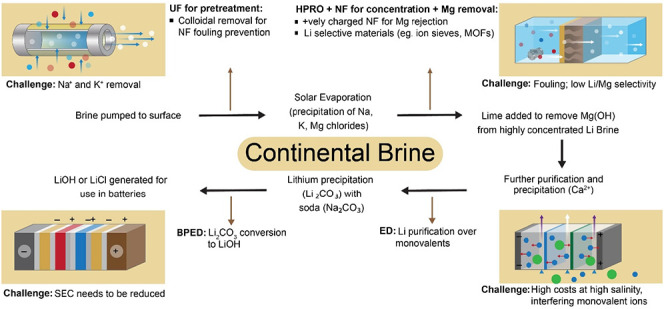

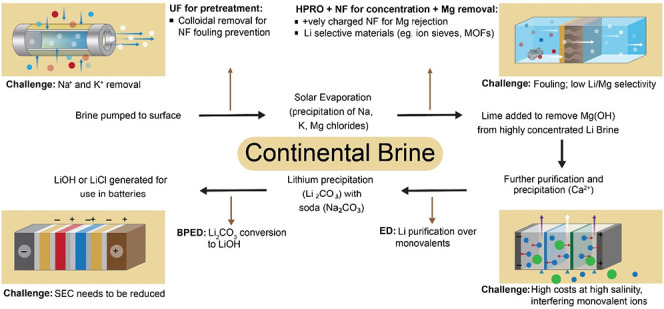

## Section 1: Introduction and Background to Lithium Demand and Recovery

Lithium is in high demand as a critical mineral^1^ because of its use in Lithium ion batteries (LiB) and the role of LiBs in electrification of the transportation and energy storage sectors. However, the extraction of lithium has raised various concerns regarding water consumption, waste generated, chemical usage, damage to local ecosystems and its high energy usage.^2,3^ Membrane technologies have emerged as viable separation technologies that enhance energy and water efficiency of separation processes. Numerous membrane processes have been developed for process intensification in various industries including wastewater treatment, desalination, chemical processing, and pharmaceutical purification.^4,5^ Membranes have also been studied extensively over the past decade for implementation in lithium extraction processes. In this work, we aim to provide an overview of the current state of membrane processes for lithium recovery, and their potential impact on the efficacy of various steps necessary for lithium recovery from its various sources.

### 1.1 Li^+^ Global Need and Scarcity

The rapid growth of the EV industry is accelerating demand for lithium at an unprecedented rate. Tesla alone estimates that by the year 2030 it would require 1000 kilotons of lithium carbonate equivalent (LCE) per year, which is almost all of the lithium produced in the year 2023 globally.^6,7^ The demand for lithium in EV batteries has approximately doubled from 2022 to 2025, and even non-EV battery demand for lithium is expected to grow by 68% from 2022 to 2025, driven by growth of energy storage systems.^6^ Lithium demand by 2030 is expected to average 3060 kT of LCE considering base case demand but can be as high as 3700 kT (approx.) if more lithium is required in battery components.^7^ Figure 1A depicts the forecast of expected demand versus supply in the coming decade and the potential shortage. As of 2023, the current planned supply can meet only 45% of the base case demand, with up to 55% supply needed to be added in the coming years.^8^

**Figure 1:**
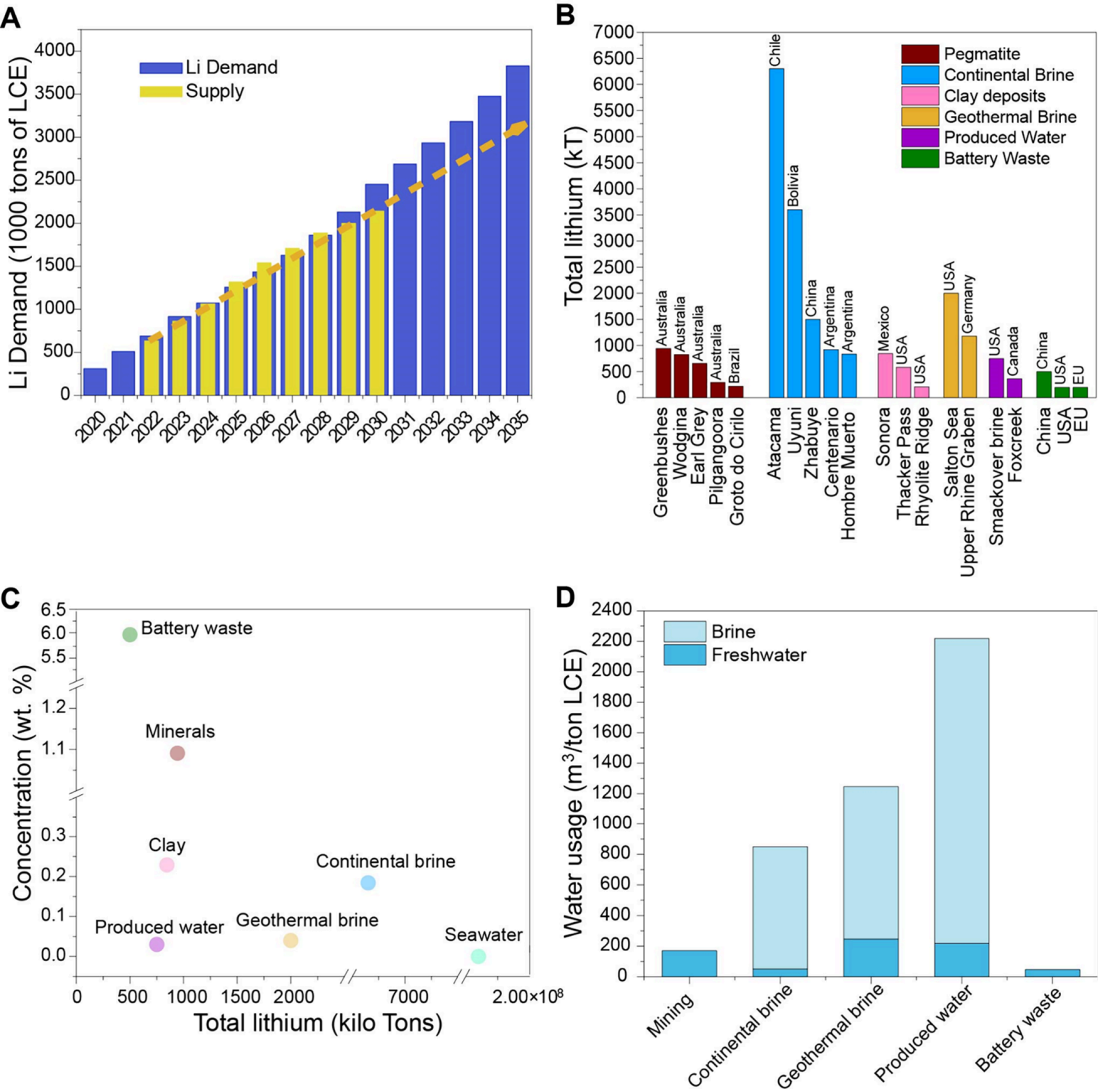
Global demand, supply, quality, and sources of lithium. (A) Global lithium demand and supply projections until the year 2035; (B) breakdown of total lithium availability in various global reserves from the largest deposits in each resource type; (C) lithium concentration versus total lithium available in each type of resource available for recovery; (D) water consumption in cubic meters for every metric ton of lithium carbonate equivalent produced in the different types of lithium rich sources. References for data in all panels compiled in Tables S1–S4 of the Supporting Information.

In terms of lithium extraction, Australia was the global leader in 2023, with 45% of the share from its spodumene hard rock mining deposits, followed by China (continental brine), and Chile (both brine and mining).^9^ When it comes to lithium refining, China holds the majority of the market share by producing 66% of the Li_2_CO_3_ and 87% of the world’s LiOH, followed by Chile and Argentina.^10,11^ Figure 1B shows the different lithium volumes that are present in the conventional and unconventional resources available for recovery. Continental brine is arguably the most valuable form of lithium resource given the high concentrations of lithium present as well as the total lithium volume, particularly in South America. For instance, the Salar de Atacama not only has very high lithium content but also a high lithium concentration (∼1.5 g/L) as well as a more easily processable magnesium to lithium ratio (MLR) of ∼6. It is worth noting that the demand for LiOH is expected to outstrip Li_2_CO_3_ as the preferred form of lithium in newer, higher energy-density battery technologies such as high nickel nickel–manganese-cobalt (NMC) batteries.^12^ This is because some nickel rich cathode materials require LiOH for synthesis, as opposed to cobalt rich batteries that can utilize Li_2_CO_3_.^13,14^ Lithium produced from ore refining of spodumene is more readily converted to LiOH, whereas brine processing for extracting lithium typically affords Li_2_CO_3_ as final product.^7^ Further processing can lead to LiOH, though this is an additional cost.

As can be expected from a resource projected to have increasing demand with a limited supply, the price of lithium has increased steadily in recent years. The price of lithium has fluctuated significantly, from $20,710 per ton of LiOH in 2021, to $72,930 per ton in 2022.^15^ This was followed by a precipitous drop, with the price forecast in 2025 expected to be $15,000 per ton of LiOH.^15^

### 1.2 Conventional and Unconventional Sources of Lithium

Lithium is available in the earth’s crust in a range of sources that vary broadly in both concentration and volume, as illustrated in Figure 1C. Lithium extraction from hard rock mining of various ores, including lepidolite and spodumene, has been carried out in various countries.^16^ The ore has to be crushed, roasted, and treated with sulfuric acid to produce a lithium concentrate, which is further treated through leaching and precipitation to produce a industrially relevant lithium compound of high purity.^17^ However, hard rock lithium mining faces several challenges including financial: high investment needs and expensive machinery, technological: high hardness of lithium-rich rocks (spodumene)^18^ and high concentration of fluorine in some sources (lepidolite),^19^ and ecological: dust generated and the use of carbon dioxide emitting furnaces.^20,21^ In general, the extraction of lithium from mining leads to greater release of pollutants than recovery from brine.^22^ The cost of lithium extraction from rock sources is also estimated to be around twice as high as recovery from brine. This has led to the development of continental brines in South America as a lithium source, in the region known as the lithium triangle (or ABC triangle) shared between Argentina, Bolivia and Chile. The Qinghai-Tibet plateau in China also contains significant lithium deposits in the brine form.^23^

The projected demand for lithium has brought other sources of lithium to prominence, with geothermal brines being significant. The Salton Sea in California, USA is a major example, with lithium concentrations as high as 400 mg/L, as well as the presence of other valuable minerals for recovery.^24^ Geothermal brine is generally the main focus of geothermal energy generation plants, but is also increasingly being explored for resource recovery.^25^

Produced water and oilfield brines are another source that has garnered attention, with the availability of this resource being more widespread than mineral ore, continental, or geothermal brines. Oilfield brines are generated in large quantities across the globe, and methods for treatment, reuse, and responsible disposal of this brine is being increasingly investigated. The relatively high concentrations of ions in this brine have made it of interest for lithium extraction. However, the concentration of lithium in oilfield brine and produced water varies greatly, with Smackover brine (brine present in the Smackover formation in southern Arkansas^26^) showing concentrations up to 500 mg/L, though the general concentration ranges from 100 to 300 mg/L.^27^ While this is lower than most continental brine sources, oilfield brine has certain advantages including the fact that the brine is readily available and does not need to be extracted, which reduces the costs of operation. The need to treat water and remove ions also increases the efficiency of the overall process as it leads to production of clean water.

The lithium content in ocean waters across the globe far exceeds the lithium on land, with a total estimated 180 billion tons of lithium, though it is present in very dilute concentrations (0.2 ppm) compared to the other sources discussed here.^28^ Low concentration represents an enormous challenge for lithium recovery, with the various evaporative and DLE techniques lacking economic viability when it comes to extracting lithium from seawater. Lithium extraction without concentration of the seawater would require an ultrahigh lithium selective membrane (or adsorbent), particularly over competing monovalent ion sodium (Na^+^), such as that demonstrated by Li et al. with a glass-type lithium selective membrane with Li^+^/Na^+^ selectivity of ∼16,000.^29^ An alternative approach could be to utilize seawater brine from reverse osmosis (RO) plants where the reject contains a high concentration of ions. Regardless, if the demand for lithium continues along the projected trends, seawater can be an avenue of interest as it prevents dependence on a small number of countries with mineral and continental brine deposits of lithium.

Lithium ion batteries (LiBs) are a composite of various valuable elements, and currently only 6% of spent battery waste is being recycled, with the rest of the waste expected to end up in landfills.^30^ With the amount of battery waste being generated expected to increase rapidly, recovery of lithium and other valuable elements is imperative. Lithium content in brines is around 0.1 wt % but in battery waste it can be as much as 5–6%. This makes battery waste a very valuable source of lithium that needs to be harnessed. The amount of battery waste that will be generated by 2030 will be 7,300 kilotons, which includes both end of life batteries as well as production scrap. This is up from just 200 kilotons that was generated in 2020.^8^ In addition, resource recovery from batteries (including not only lithium but also other valuable metals such as nickel and cobalt) could also generate revenue amounting to $6 billion in the year 2040.^8^ Recycling from battery waste may account for up to 6% of the global production share of lithium by 2030.^31^

### 1.3 Water Consumption and Waste Generation

Most of the economically recoverable lithium on earth lies in the lithium triangle of South America, present in highly arid regions within salt lakes known as salars. The conventional process of lithium extraction through evaporation for concentration of lithium in the water is widely employed in this region. Indeed, the extraction of lithium through this process is possible due to the aridity of the region, which allows for year-long conditions of water evaporation at a high rate.^2^ This site specific and large “biome scale” process of recovering a technological relevant mineral is an example of the growing concern over “problem shifting”,^32^ where the electrification of the transportation industry goes hand in hand with the growing freshwater scarcity of the lithium rich nations if conventional lithium recovery technology continues to be employed.^33^ Water usage in lithium recovery has only been studied carefully in recent years, and observations from water table levels and soil moisture point toward a significant effect of these recovery processes on the local water levels. Using Salar de Atacama in Chile as an example, an analysis of the groundwater shows that there is very little inflow into the underground aquifer, with most of the precipitation being evaporated before it reaches the groundwater. A water budgeting study indicated that freshwater use in this region does not appear to be sustainable.^34^ Once this saline brine is pumped up to evaporation ponds, up to 95% of the brine needs to be evaporated before the lithium concentration reaches desired levels.^35^ Depending on the lithium recovery rate and the concentration of the brine, up to 800,000 L of water could be evaporated to produce 1 ton of Li_2_CO_3_.^2^

There is also a distinction between the brine used for lithium recovery, and the freshwater used in various stages of the recovery process, such as preparation of the lime and soda solutions, scrubbing solvents used for boron removal, and for purification of the lithium carbonate produced. This freshwater is typically pumped out locally by operating companies. Depending on the porosity and permeability of the rock formation surrounding the salar, freshwater may be drawn into the salar once lithium recovery has begun and the salar concentration increases and is placed under stress.^36^ Mining advocates argue that the size of evaporation ponds and the amount of brine being used is much lower than the brine present in the underground salars, and hence is not consequential in the overall hydrogeological cycle. There is a need for more extensive studies to analyze the effects of brine extraction on the overall water stability of the region. However, as a rough estimate, up to 50,000 L (or 50,000 kgs) of freshwater is used per ton (1000 kg) of battery grade lithium produced.^2^ Likewise, over 115,000 kg of solid waste is generated per ton of Li_2_CO_3_, most of which are the precipitates of various salts in the brine.^2^ Figure 1D shows the different water use classes in the lithium recovery processes, and the large amount of brine water that needs to be processed. Considering that an average plant that produces 20,000 tons of lithium a year, over the course of a decade the enormous waste generated can cover a region of over 11 sq.km at a height of 1 m. It is imperative that the waste generated be handled in a sustainable manner The segregation and repackaging of the various salts so that they can be sold on the market might be a desirable solution that needs to be explored. For example, magnesium which is listed as a critical raw material by the EU, is generated in large quantities during lithium recovery.^37^

DLE technologies were proposed and developed in part to tackle the need for evaporation ponds and minimize the operation time as well as water lost. However, even mature DLE such as adsorption and ion-exchange can require large amounts of water as eluent. Indeed, some studies on DLE technologies indicate freshwater requirements that are ten times higher than the evaporitic method.^2^ For instance, one of the full-scale DLE technologies (selective adsorption) deployed at the Salar del Hombre Muerto (Argentina) reports an overall freshwater usage above 70,000 L per ton of Li_2_CO_3_ produced annually,^38^ which is higher than the requirement for evaporitic technology. Liquid–liquid extraction is also a technology that has high capacity and needs relatively simple equipment for recovery,^39^ but drawbacks such as the requirement of large equipment that can undergo corrosion,^40^ as well as large liquid solvent volumes needed that can be highly flammable, such as kerosene.^41^ Processes like ion-exchange and LLE can also require adjustment of pH,^42^ as well as needing acids as stripping agents and expensive extractants,^41^ which can require significant chemical quantities. Hence we see few examples of scaled-up application of this technology for lithium recovery.^41^

Another less-discussed topic is the fate of the spent brine generated in the recovery process, particularly if DLE technologies are used and most of the liquid is not evaporated. Conventional experience with resource recovery from brine suggests that the reinjection of brine into underground layers is the expected solution. However, this needs to be considered thoroughly. There is also the consideration that most DLE technologies would inevitably leave chemical residues in the solution, such as Mn or Al in the case of adsorbents, or organic solvents in the case of LLE. The pH and alkalinity are also affected, such as in the case of ion-exchange. These questions have not been explored in research.^43^ This modified brine will have changes on the salar, and the existing microbiology and flora can be affected by these changes.^16^ The brine may also cause dilution of lithium in the source salar if the two water bodies mix over time.

Membranes can provide a DLE solution with minimal use of leftover chemicals in the brine, as well as offer solutions such as minimum liquid discharge (MLD) and zero liquid discharge (ZLD). These solutions are already being explored for desalination plants globally.^44,45^ In an ideal scenario, these technologies could greatly minimize the freshwater extraction carried out onsite and fulfill most of the needs at the plant. This is described in more detail in later sections.

### 1.4 Overview and Commercial Status of DLE Technologies

Several separation technologies, initially developed for separation applications such as desalination and wastewater treatment, have recently been deployed for lithium recovery from brines and other unconventional sources as alternatives to solar evaporation.^46^ In this subsection, we provide a brief overview of the DLE technologies that have been implemented for lithium recovery or have a high technology readiness level (TRL) (Table 1).

**Table 1:** Overview of Advantages and Disadvantages of Various DLE Technologies^a^

technology	description	pros	cons	TRL	companies
adsorption	LiCl is physically adsorbed onto a sorbent and recovered with an aqueous strip solution	high lithium selectivity	limited to brines with relatively high salinity and temperature	9	Livent
		low energy and water consumption			Eramet
					Summit Nanotech
					SunResin
		simple operation	requires additional processing		International Battery Materials
					Koch Technology Solutions
					Vulcan Energy
ion exchange	resins exchange lithium ions from a feed solution with hydrogen from an acidic rinse	can operate on low lithium content brines	high cost	8	Lilac Solutions
		low energy and water consumption	resin durability		
			requires acid inputs		
solvent exchange	an organic solution consisting of a solvent and extractant selectively extracts lithium from brines	high lithium recovery	high cost	7	Solvay
		does not require additional processing	requires chemical inputs		
membranes	a lithium-selective membrane separates lithium via pressure-driven (nanofiltration) or potential-based processes (electrodialysis)	low energy and water consumption	limited to brines with low Na^+^/K^+^ content	4–5	DuPont EnergyX
			requires additional processing		

a TRL values taken from reference 46, other information sourced from references cited in text.

#### Adsorption

Adsorption is the most widely adapted DLE technology and has been implemented across the world.^42^ Generally, adsorption involves aluminum-based Li–Al layered double hydroxides (Li–Al LDHs) in the form of spherical beads that adsorb lithium through intercalation and deintercalation at interstitial sites on their surface.^47,48^ Li–Al LDH can be used for longer adsorption–desorption cycles and are easier and cheaper to manufacture than other sorbents.^49–51^ However, these adsorbents have lower selectivity and adsorption capacity compared to manganese and titanium-based adsorbents and require relatively high temperatures (due to the endothermic nature of Li^+^ adsorption on Al-based adsorbents) and Li^+^ concentrations in the feed to be competitive.^46^ Additional drawbacks include the higher cost of equipment and need for further processing as a result of impurities in the eluent.^42^

Aluminum-based sorbents developed by companies such as Livent, Eramet, Summit Nanotech, and SunResin are already at the commercial operation stage.^46^ Livent’s Project Fenix, established in 1997, was the first commercial-scale DLE operation in Argentina.^38^ Livent’s established process begins by pumping lithium-rich brines from production wells in Salar del Hombre Muerto and transporting them either to a selective adsorption plant as direct feed or to preconcentrate ponds to enhance production capacity. During selective adsorption, feed brine is loaded into columns containing proprietary aluminum-based sorbents, which are then stripped with fresh water to elute lithium brine. This product brine is concentrated in evaporation ponds to produce a concentrated solution which is then converted to lithium carbonate using sodium carbonate. The spent brine produced during this process is returned to the salar via infiltration, and freshwater is recycled for sorbent regeneration. Livent’s projected lithium carbonate production is expected to reach 98,000 t per annum by 2030 following proposed expansions to increase brine production, improve lithium recovery, and reduce freshwater consumption.^38^ Production capacity of other commercial sorption based DLE technologies range from 20,000 to 90,000 t per annum.^42^

#### Ion Exchange

Lithium titanium oxides (LTO) and lithium manganese oxides (LMO) are classified as lithium-ion sieves and are mainly used in ion exchange, which is another sorption-based technology that has achieved a high TRL.^46^ Ion exchange uses these sorbents to exchange lithium ions from a feed solution, which are then swapped with hydrogen through an acidic rinse for regeneration. LMOs demonstrate high lithium selectivity, capacity, and excellent regeneration.^46,52^ However, these manganese-based adsorbents are expensive to produce, unsustainable due to manganese dissolution,^53^ and sensitive to acidic conditions that can lead to disintegration of adsorbent.^54,55^ Titanium-based adsorbents have high selectivity and are more robust in harsh chemical environments due to the stronger bonds between the atoms. They are also easier to operate for large number of cycles. Though ion exchange can achieve ∼90% lithium recovery for low lithium concentration brines at a low energy and water consumption, it remains limited by high capital and operational costs, limited resin durability, and required acid inputs.^42^ Additional studies on these adsorbents are needed to address these lower lithium uptake kinetics.

Ion exchange technology is in the demonstration phase and is led by Lilac Solutions.^42^ Similar to selective adsorption technologies, Lilac uses columns packed with proprietary ceramic beads to absorb lithium ions, which are then recovered by flushing with dilute hydrochloric or sulfuric acid. The resulting lithium chloride or sulfate is then converted into lithium carbonate or hydroxide.^56^ Lilac has built three DLE pilot plants in Argentina and the US – the Kachi Project, Salton Sea, and Hell’s Kitchen – and expects a lithium production capacity of 25,000 t per annum for each plant starting in 2024.^42,56^

#### Solvent Extraction

Solvent extraction or liquid–liquid extraction uses an organic solution consisting of a solvent and an extractant to form complexes with lithium and separate them from the feed, after which lithium is recovered and the organic phase is regenerated for reuse. The type of extractant used can be typically classified by diluents/solvents such as kerosene and ionic liquids, extractants with high lithium affinity such as crown ethers.^41,57^ Kerosene mixed with ferric chloride (FeCl_3_) has been considered as an excellent solvent for this purpose, and other ketone and beta-diketone based solvents have also been proposed for this purpose.^58^ While solvent extraction is a well-understood process that is easy to set up, its drawbacks include the processing challenge of solvent-contaminated brine disposal, cost of organic solvent, and corrosion from the use of these solvents.^42^ More research is needed to deploy this technology reliably for lithium recovery.^58^ Solvent extraction is a pilot-phase DLE technology led by Solvay and their phosphorus-based Cyanex 936P, which is the leading extractant for divalent-free brines at alkaline pHs.^59^

#### Scale of DLE Technologies and Membrane Technologies

Membrane processes are generally energy efficient, easy to scale up, and applicable throughout the lithium recovery process across a variety of lithium sources.^60^ Indeed, while considering the scale of brine that needs to be processed at an average recovery plant for lithium brine, it is important to consider that membranes for desalination are one of the few processes currently deployed successfully that can process such volumes with a minimal footprint and decades long know-how. Figure 2 depicts some of the largest scales at which membranes have been deployed in desalination and wastewater processes, which give them a distinct advantage over some of the DLE technologies when it comes to the question of processing large volumes of brine water to recover the required amount of lithium for profitability.

**Figure 2:**
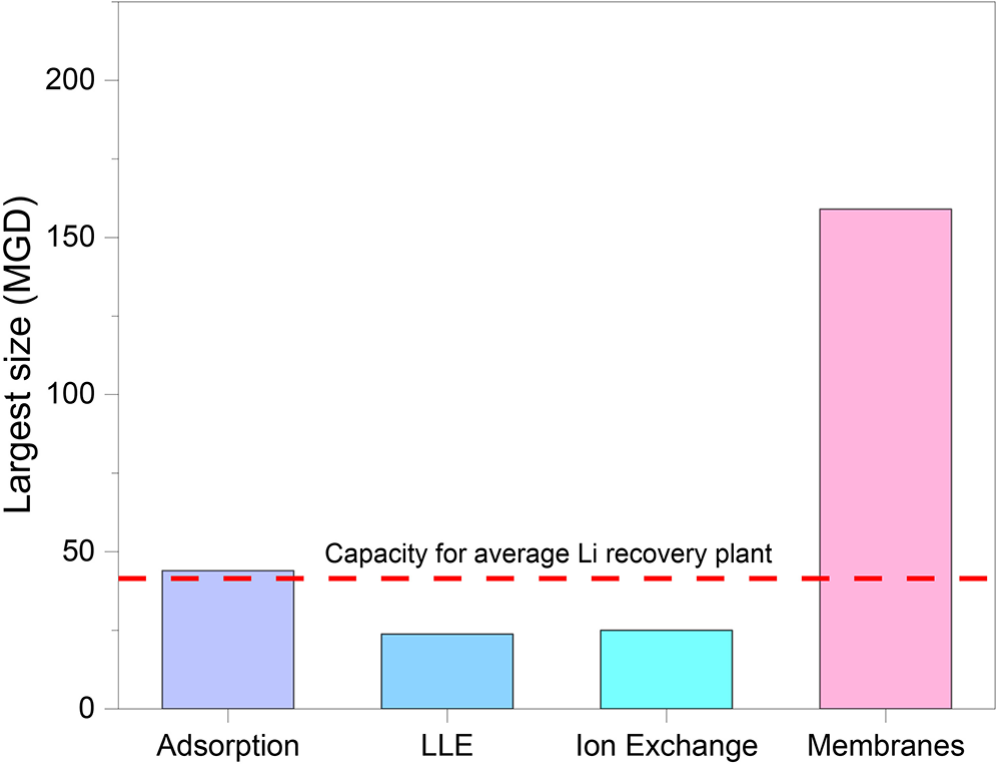
Comparison of some of the largest commercial units for each technology and how it compares with the requirements for brine processing volumes in lithium recovery. Plant sizes used compiled in the Supporting Information, Table S5.

Traditional (evaporation based) lithium recovery methods have processing timelines of months to years. In contrast, DLE technologies including membrane technologies have a much shorter turnaround time as well as production period for lithium (hours or days). Thus, DLE technologies are much more suited to responding to fluctuations in demand and price. Conventional evaporitic technology can also struggle with rapid increases in demand, as construction of new ponds is very capital intensive. On a related note, conventional evaporitic recovery is highly source specific and strongly dependent on weather and elevation, and requires years of planning, piloting and feasibility studies before a plant can be constructed. This makes investment decision making for these plants highly complex. The development of DLE and membrane technologies that can handle a broader spectrum of source salinities and on-site variables can significantly reduce the time needed to conduct feasibility analyses, and lead to increased trust in the robustness of the technology. It can also minimize the high dependence that local weather and aridity has on the lithium recovery, which is of increasing importance with erratic weather patterns that could potentially affect regions of aridity.^61^

Current nanofiltration membranes can be scaled up with minor modifications for Li^+^/Mg^2+^ separation,^62^ and ultrafiltration can offer solutions in pretreatment of the feed stream as well. Electrodialysis (ED) has shown promise in recovery from seawater.^29^ DuPont has released a class of nanofiltration membranes, FilmTec LiNE-XD and LiNE-XD HP, for lithium separation from divalent ions.^63,64^ Additionally, EnergyX uses membrane-based ED and bipolar membrane ED (BPED) as a part of its LiTAS (Lithium-Ion Transport and Separation) suite alongside adsorption and solvent extraction.^65^

However, there are still challenges associated with this process, such as the need for high Li^+^/Na^+^ selectivity, and enhanced water recovery after treatment. Membrane fouling and scaling remains an ever-present challenge when dealing with complex feedwater systems. We will discuss the potential and limitations of membrane processes across the lithium recovery lifecycle in subsequent sections.

## Section 2: Conventional Recovery Processes and Membrane Applications

In this section we lay out the most practiced conventional strategies to recover lithium from various sources and then describe the potential roles for membranes at different stages. While certain specific stages of lithium recovery from particular sources have been studied in detail for membrane application (such as for Li^+^/Mg^2+^ separation in brines; bipolar membranes for LiOH production) there are several other steps where membranes have the potential to be applied but have not been explored. The need to rapidly scale up lithium recovery is urgent, and membranes may already provide solutions at different stages to be able to reach production goals. We also provide preliminary energy and water savings possible by replacing conventional processes currently applied with membrane technologies for continental brine, geothermal brine and produced water. Energy expenditure and water usage in the following sub sections were determined from technical reports available from various commercial operations (these are cited in Supporting Information). Calculations were not carried out for membrane-based lithium recovery from seawater and battery waste due to the absence of full-scale commercial applications at an industrial scale for these sources.

### 2.1 Continental Brine

Continental brine contains the largest deposit of lithium on land across the globe, and the typical lithium concentrations in these brines are above 500 ppm. South American brine contains ideal conditions for the conventional lime-soda evaporation process, with the yearlong arid climate as well as high elevation locations which leads to high irradiation. Both these factors contribute to a steady and high rate of evaporation that is needed to concentrate the brine. The brine locations in China do not have arid conditions and are also plagued with high magnesium to lithium ratios making recovery much more challenging.^66^

In the conventional process (lime-soda evaporation process), the brine is initially pumped up from underground salars from a depth of ∼300 m into large evaporation ponds lined with impermeable liners. Here, the brine is concentrated by water evaporation over a period of 10–24 months, depending on the concentration of the ions. This leads to the precipitation of the Na^+^ and K^+^ ions from the solution. Once the lithium has been concentrated to a level close to 5000–6000 ppm the brine can be moved on for further processing. The major challenges at this stage include the need to remove boron, magnesium, and sulfate ions. Boron can affect the performance of the battery grade lithium end-product and is removed by solvent extraction using an organic solvent. Magnesium and sulfate ions are removed by the addition of CaO (lime), which is prepared on site. This leads to the precipitation of MgSO_4_ that is extracted from the brine. Depending on the presence of calcium, further processing is required to remove this divalent ion as well, and the calcium generated can be reused in the process as lime. Next, residual concentrations of Na^+^ and K^+^ need to be removed by brine polishing. The current battery grade lithium purity required is ∼99.5% and processes such as ion exchange and solvent extraction can be used here to remove the low levels of impurities. Next, Na_2_CO_3_ is added to the brine to induce the precipitation of Li_2_CO_3_, which is again solubilized and further purified. In the final stage, either crystals of Li_2_CO_3_ can be generated, or a further process can be carried out with calcium hydroxide to prepare LiOH, the desired lithium compound for battery technology.

Here we analyze the different stages (represented by specific unit operations) in conventional continental brine process described above to determine where membrane processes can be applicable (see Figure 3). We propose that microfiltration (MF), ultrafiltration (UF), and nanofiltration (NF) can be employed in pretreatment of the brine to remove colloidal particles and dissolved salts, respectively. Colloidal silica is a common presence in these brines along with other trace elements such as iron and aluminum, and these can be targeted for removal using UF. Traditionally, silica removal is carried out through lime softening and coagulation, and membranes have not been studied extensively in this context. The concentration of divalent ions can also be reduced at this stage if NF is employed.

**Figure 3:**
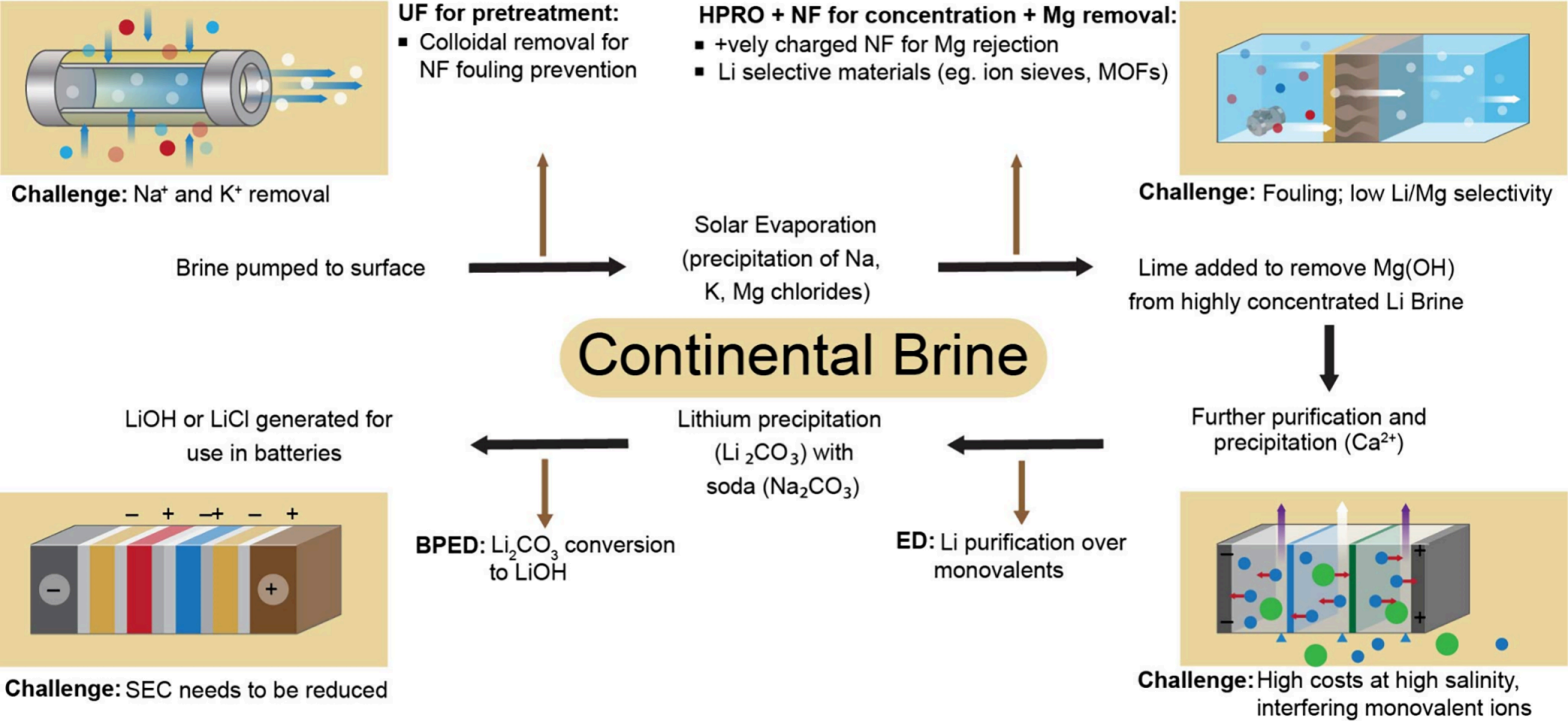
Process flow of the conventional lime soda evaporation process for Li^+^ recovery from continental brine and membrane process replacements.

Next, the evaporitic stage in pond evaporation is considered. This is an important step for various cost, energy, and sustainability reasons, because of (i) the quantity of water lost through evaporation, (ii) need for large land areas to build ponds, (iii) duration of evaporation extending to years, and (iv) the capital costs (CAPEX) of building these ponds. This is also a challenging stage for membranes to be applied for multiple reasons, including (i) the high feed concentrations (Na^+^ concentrations from 50,000 to 70,000 ppm), (ii) high pressure or electricity requirements at these concentrations, (iii) membrane fouling and scaling considerations, and (iv) poor monovalent-monovalent ion selectivity in most current membranes. One of the potential applicable technologies is selective electrodialysis (SED), which can utilize a single-ion selective cation exchange membrane (CEM) for separation of Li^+^ from the feed. The Lai group demonstrated an ion-sieve Li–Ti glass membrane with a high degree of Li^+^ selectivity.^29^ However, the energy requirements for ED rise significantly with brine concentration. At increasing concentration, greater number of ions need to be transported across the IEM which requires more energy in the form of higher electrical potential.^67^ Rising concentration polarization also increases the concentration gradient, which can lead to the back-diffusion of counterions into the diluate stream.^68^ When monovalent selective IEMs are used, Na^+^ and K^+^ ions migrate through the CEM preferentially compared to Li^+^, which is another challenge for ED in Li^+^ recovery.^69^ A solution to this problem can be using ED in series, to minimize both the electrical potential required and the large variations in concentration gradient across membranes. Another application for consideration is high pressure RO (HPRO) for brine processing, that has been employed in certain ZLD and MLD strategies. Current HPRO modules can operate at 120 bar, and there is also development of HPRO that can operate at pressures up to 200 bar. However, these utilize conventional polyamide membranes that do not have the required selectivity to concentrate lithium over the other monovalent ions. The development of TFC membranes that provide the desired selectivity while operating at these pressures is an enormous challenge.

The next step in lithium recovery that can be targeted by membrane replacement is magnesium removal, and in this case, membranes are well-suited for the process. Conventional negatively charged NF membranes already allow for an appreciable monovalent-divalent selectivity that may well be sufficient (NF90 has Li^+^/Mg^2+^ selectivity of ∼20).^62^ There are many studies in literature currently that evaluate the application of NF membranes for this step, and various modifications have been carried out to enhance efficiency. The development of positively charged NF membranes that can employ Donnan exclusion for high Mg^2+^ rejection based on divalent charge is the most popular strategy that can be relatively easy to scale.^70^ There are also studies analyzing narrow pore size distribution for size sieving effects, and the implementation of Li^+^ selective chemistries such as crown ethers,^71^ MOFs^72,73^ and 2D materials^74^ to enhance this separation. Ideally, NF can be employed most successfully in the Li^+^ recovery process with relative ease, as they can provide a high degree of purity and recovery in this separation.^62^ Substituting liming with membranes will also negate the need to continuously transport large quantities of lime into the salar regions as lime demands will grow with the installation of newer lithium recovery plants.

Conversion of Li_2_CO_3_ to LiOH is a process that requires energy input, chemicals, and large equipment. This process also generates chemical waste. While BPED has been employed for Li^+^ recovery,^75^ it may have greater utility in the conversion of Li_2_CO_3_ to LiOH^76^ and such applications have been reported in literature.^77^ There is a need to develop superior BPED membranes that can carry out this process more efficiently, generating high concentrations of LiOH (currently limited to 3–5 wt %), as well as membranes with lower resistances and lower area requirements.^75,78^ The potential to pair BPED with solar energy that is easily available at these locations could make this stage of the process much more optimum at a system design level and remove one of the limitations of brine recovery.

One possible version of membrane process application has been evaluated to show how common processes such as NF, HPRO and BPED can be utilized in the process flow for lithium recovery. In Figures 4 **A and B**, we evaluate the potential cost and water footprint differences between the conventional evaporative process and a combination of membrane processes with HPRO, ZLD, and BPED. Process details were taken from the MSB Blanco lithium project from the Atacama region in Chile. Details for NF, HPRO and ZLD (specific energy consumption and recovery rates) are taken from various techno-economic analyses.^79–81^ Details of calculations and data used are presented in Supporting Information, Appendix C, Tables S6 – S9.

**Figure 4:**
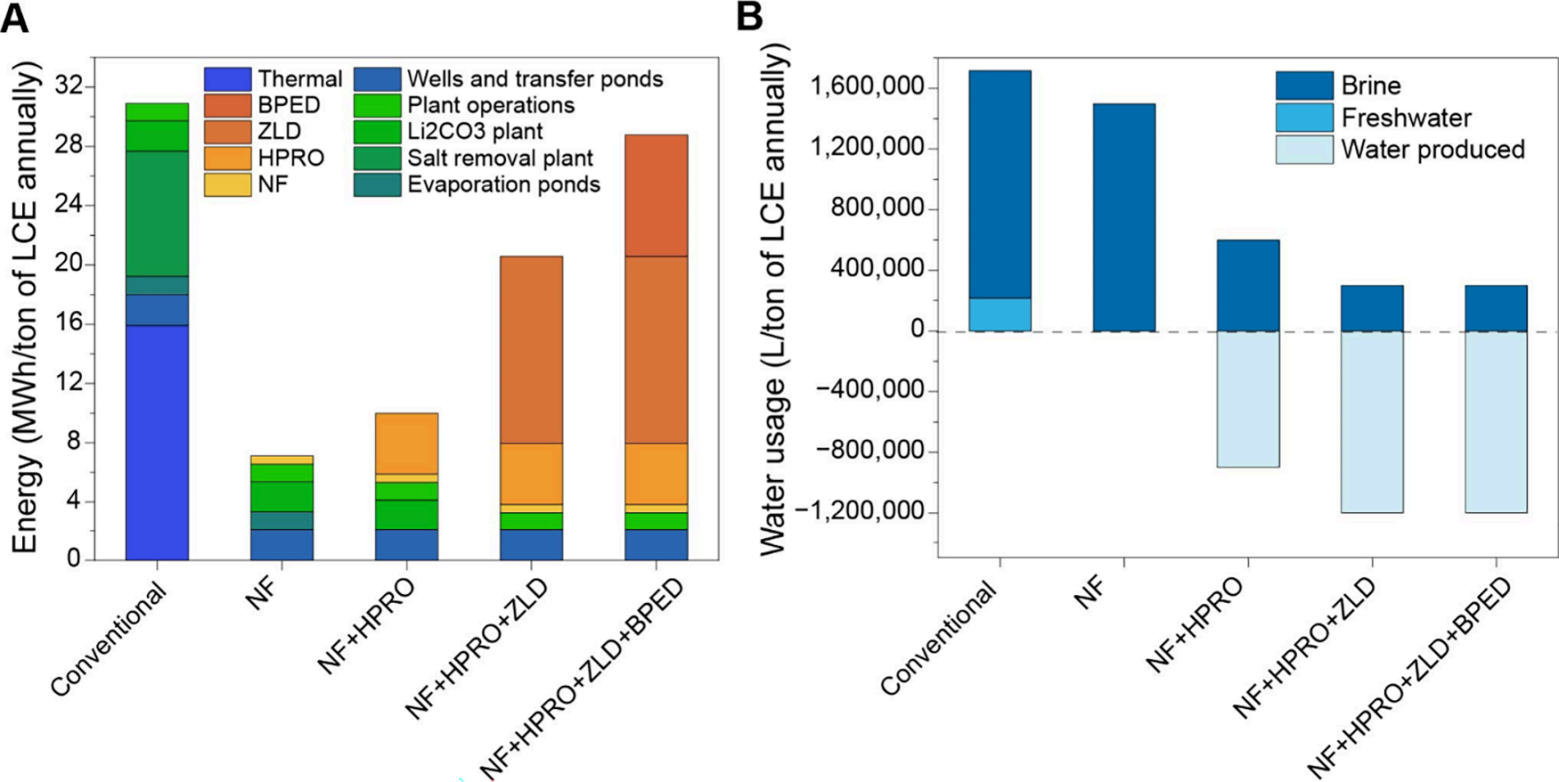
(A) Potential energy savings through replacement of conventional process with various membrane technologies (B) Potential water savings through replacement of conventional process with various membrane technologies. Calculation, sources, and values for panels A and B presented in the Supporting Information, Appendix C, Section 1 and includes Tables S6–S9; NF = nanofiltration; HPRO = high-pressure reverse osmosis; ZLD = zero liquid discharge; BPED = bipolar membrane electrodialysis.

It is important to note that these figures are idealized, and the membrane process has not been simulated with details such as auxiliary costs and other minor expenses. Other membrane processes can be substituted in as well, which are outside the scope of this analysis. Similarly, the water savings and solid waste valorization are ideal case scenarios. Keeping these limitations in mind, this preliminary analysis allows us to see the potential for savings and efficiency with membrane technologies. The potential advantages of the different steps included in each option are shown in Table 2, and choices can be made based on different priorities such as water savings and chemical conversion needed. The CAPEX costs are likely to be significantly lower as well, as evaporation ponds can be eliminated from the cost, while thermal energy required for Mg removal and crystallization can also be eliminated. The water recovered from the membrane ZLD processes can be utilized to fulfill any on-site needs for the plant without stressing local water sources, while the solid waste can be sold with valuable K^+^ and Mg^2+^ compounds generated.

**Table 2:** Value Proposition in Energy and Water Savings for Different Process Combinations

	value proposition			
process	ion selectivity	water recovery	brine transportation/disposal	valorization
conventional	√			
NF	√			
NF + HPRO	√	√		
NF + HPRO + ZLD	√	√	√	
NF + HPRO + ZLD + BPED	√	√	√	√

From this analysis we can observe that different membrane processes allow opportunities for energy and water savings, and the implementation of these processes can be customized based on the requirements of individual brine sources, which is important since individual lithium reservoirs can vary significantly.^2^ The implementation of these processes can also vary based on local regulations and social demands. Greater water savings can be targeted in areas of high aridity, while energy requirements can be considered based on availability of renewable sources that are easily linked with membrane technologies. While detailed techno-economic analyses are necessary to determine if these savings are realistic, our analyses provide a starting point for consideration for membrane technologies.

### 2.2 Geothermal Brines

Geothermal brines have some distinct features that distinguish them from continental brines (see Figure 5). The concentration of Li^+^ in these brines at 100–400 ppm and is thus typically lower than that of continental brines. The location of geothermal brines might also be closer to urban areas with less land area available for pond development, such as the Salton Sea in California and the Rhine region in Germany.^82^ These two factors indicate that it is impractical to utilize the evaporitic method for brine concentration, as the time required to achieve the necessary lithium concentration is unreasonable. Finally, these brines are available at an elevated temperature (250–300 °C) which necessitates certain considerations when selecting a DLE process.^24^ Issues such as corrosion and scaling can become more severe, and several membrane processes can no longer be applicable at these temperatures. The increased possibility of corrosion increases the importance of membrane pretreatment, for the removal of silica as well as reducing the concentration of Ca and Mg scalants in the feed.^83^ The long-term stability of the plant could be dependent on the effectiveness of this pretreatment which can be carried out by NF membranes. On the other hand, the heat content provides energetic advantages for use with heat assisted membrane processes such as membrane distillation.

**Figure 5:**
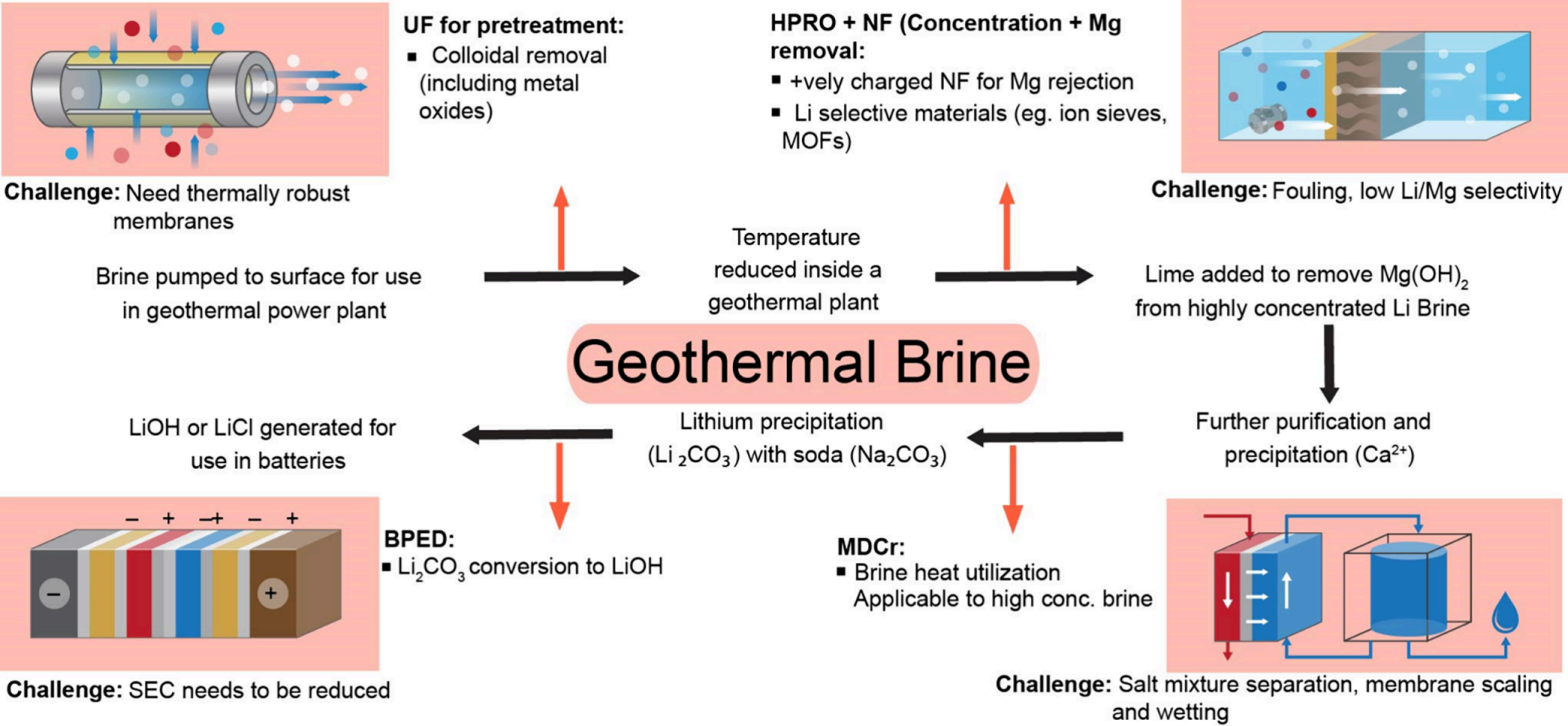
Process flow of conventional process for Li^+^ recovery from geothermal brine and membrane process replacements.

Many of the challenges in Li^+^ recovery from geothermal brines are similar to continental brine, membrane technologies proposed for continental brines remain relevant here. An exception is the possible use of membrane distillation (MD), which becomes more attractive in this case. The presence of a feed at higher temperature removes the requirement of thermal energy input for membrane distillation, which can be employed more readily after sufficient pretreatment. A process such as membrane distillation-crystallization (MDCr) has been tested for this step of lithium recovery.^84^ MDCr can operate at high brine salinities and provide the desired water removal for brine concentration, while also preserving lithium in the feed. The crystallization step can allow for the precipitation of Na^+^ and K^+^ ions as well. Essentially, this process can replicate the evaporitic process with a much lower footprint and much shorter duration. However, MD has yet to be employed at a large scale and remains a developing technology. Alternatively, MD can also be employed at the end of the process for brine management since it can tolerate very high salt concentrations at an elevated temperature.^85^

Further, an analysis of pilot plants being tested for Li^+^ recovery from Salton Sea make it clear that the geothermal brine that is extracted is initially used to generate geothermal energy, following which it is processed for resource recovery^17,24^ ). The presence of a geothermal power plant in the process increases the overall sustainability of the recovery, while also potentially reducing the brine temperature to a more reasonable level for membrane stability while at a level that is still advantageous for membrane distillation. An analysis of DLE technologies being tested at the Salton Sea by Warren led to the conclusion that the lithium generated from geothermal brines is economically viable and competitive, which can also be seen in our analysis in Figure 6A,B based on the Vulcan Energy plant in Upper Rhine Valley, Germany.^17^ Geothermal brines are ideally placed to be the next source exploited for lithium recovery.

**Figure 6:**
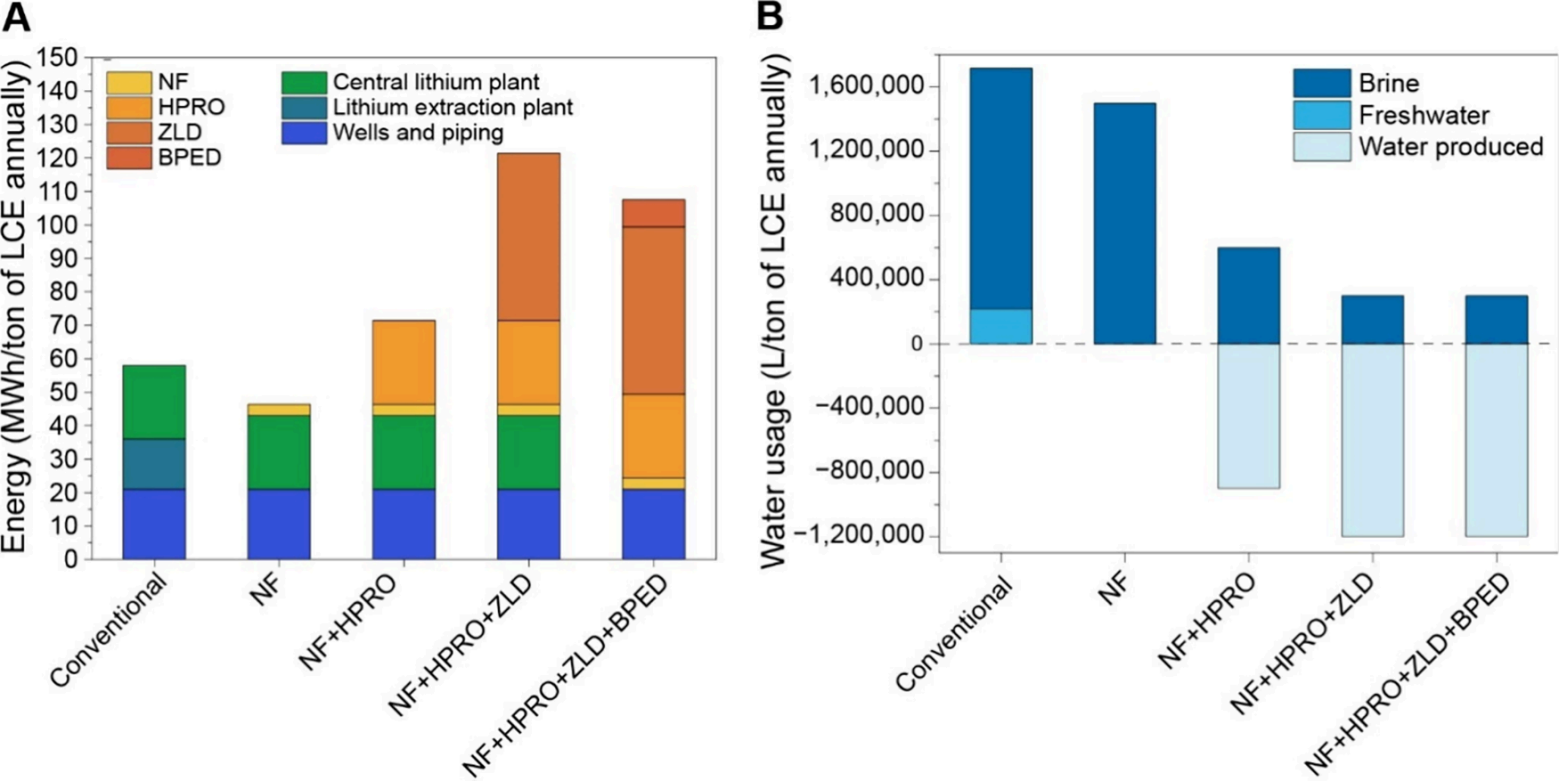
(A) Energy savings through replacement of conventional process with various membrane technologies. (B) Water savings through replacement of conventional process with membrane technologies. Calculation, sources, and values for panels A and B presented in the Supporting Information, Appendix C, Section 2 and includes Tables S10 and S11; NF = nanofiltration; HPRO = high-pressure reverse osmosis; ZLD = zero liquid discharge; BPED = bipolar membrane electrodialysis.

### 2.3 Seawater and Seawater Reverse Osmosis brine

Seawater is one of the largest sources for a wide range of elements on earth, from common elements such as sodium, potassium, magnesium etc. to highly valuable elements including gold and uranium, and lithium is no exception.^86^ Seawater contains up to 5000 times as much Li^+^ as contained on land on a total mass basis, which makes it a near-unlimited source.^87^ This is in contrast with Li^+^ on land, which may be exhausted within a century at the current rate of demand and extraction.

However, the concentration of Li^+^ in seawater is extremely low (0.1–0.2 ppm), particularly when compared to other ions such as Na^+^ (roughly 12,500 ppm).^29^ This makes Li^+^ recovery extremely challenging and unproductive when it comes to seawater, particularly with the technologies and separation processes available currently.^88^ Despite this severe limitation, there are certain advantages to pursuing Li^+^ recovery from seawater. While Li^+^ on land is locked in rocks and brine that can be difficult to access, seawater is readily available for processing in coastal areas. The brine sources on land are present in regions that are by necessity water-scarce, and the process of extracting Li^+^ can be taxing on these regions and exacerbate existing water scarcity issues.^36^ By comparison, seawater Li^+^ extraction can be carried out along any coastline. Li^+^ locked in various brines is a limited resource, with diminishing returns as the process goes on, while seawater can provide a stable and consistent source for processing. While mineral deposits and brine resources (especially of sufficient size to make extraction feasible) are limited to a total of 10–12 countries, seawater extraction can be carried out in a much larger number of countries. This is desirable for the sake of energy independence and distribution of resources perspective. Finally, an increasing number of countries already process water through various desalination plants globally for potable water needs, which means that the infrastructure required for seawater resource recovery is already partially available, with reliable expertise and technologies that can be deployed. While lithium recovery from geothermal brine has been the focus of majority of research in this area, there has been a sizable effort in studying resource recovery from seawater as well.^29,87–91^ The recovery of Li^+^ from seawater will be of interest to various countries in the coming years, and it is important to analyze various cases and determine if a sustainable pathway exists for stable recovery of Li^+^ from this source.

Membrane technologies are ubiquitous across desalination and its pre- and post-treatment processes (see Figure 7). UF and NF technologies have been well studied and deployed in pretreatment for the reduction of colloidal, microbial, and inorganic foulants from the feed. The importance of effective pretreatment using membranes and its effects on the overall process efficiency and energy use has been determined as a very important factor in desalination plants.^92^ When it comes to feed concentration and lithium recovery, we face a similar issue of monovalent selectivity in membranes. As mentioned earlier, Li et al. developed a membrane for seawater lithium recovery that showed desirable results over a 5-stage separation process, with a Li^+^/Na^+^ selectivity in a single stage over 16,000.^29^ The scalability of these glass-like membranes remains an unanswered question. For polymer membranes in pressure-driven processes, the Li^+^/Na^+^ selectivity remains <2–2.2, which is not sufficient for seawater recovery. The development of a membrane that can successfully carry out this separation with the help of nanoparticles such as crown ethers and MOFs is crucial, as well the scalability of these membranes, which is an area of ongoing research.^93,94^ An alternative is the utilization of the RO brine for recovery, which has higher concentrations of lithium. A “second pretreatment” step can be utilized after the RO brine is generated through the use of NF, which can further remove divalent ions before the water is sent for lithium recovery.^95^ Various MLD and ZLD technologies are being studied, and resource recovery from RO brine has been the subject of much deliberation in the membrane and desalination communities. The idea of valorization of the RO brine and pursuing a circular economy strategy requires the development of highly ion-selective membranes, with lithium recovery from seawater expected to gather more interest in coming years if the demand for lithium batteries in electric vehicles and energy storage remains as important as it is today.

**Figure 7:**
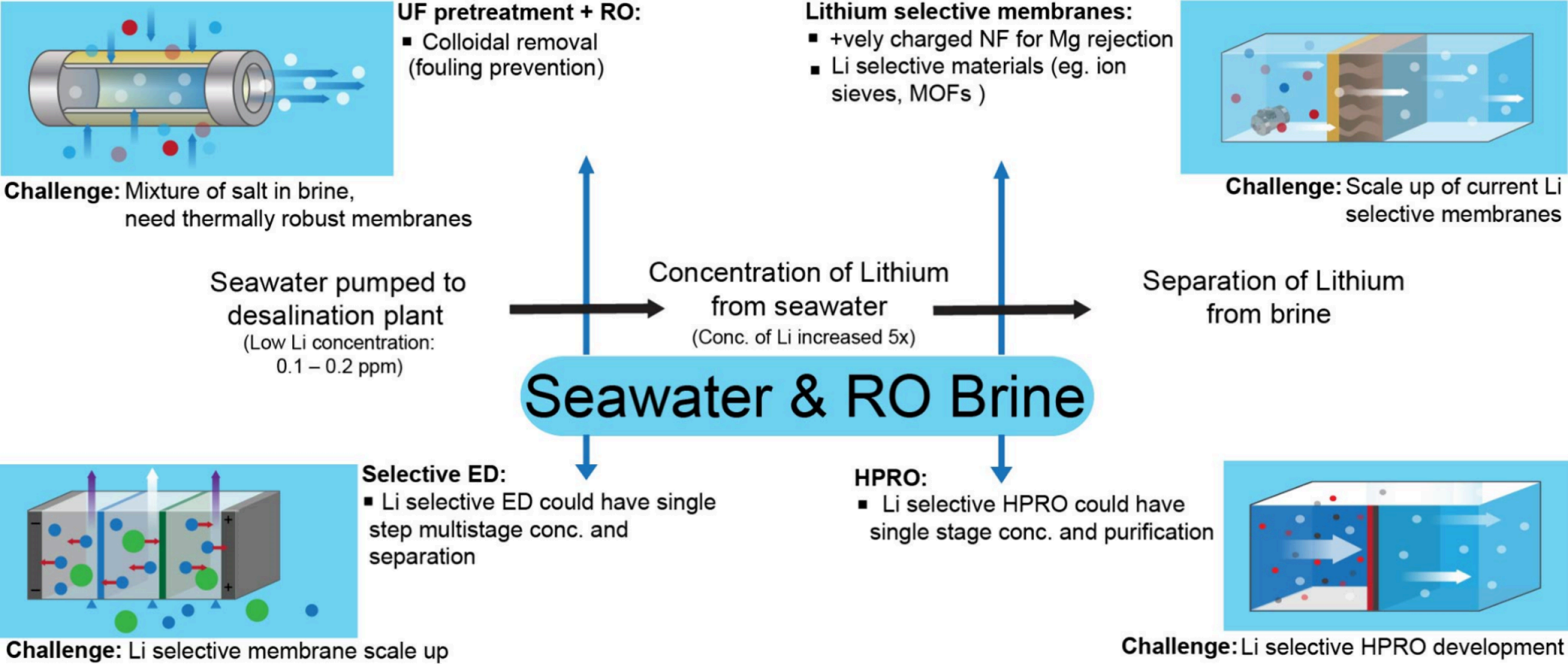
Seawater reverse osmosis followed by brine treatment for lithium recovery.

### 2.4 Produced Water

Oilfield water is present in underground reservoirs that are adjacent to oil reserves, which contain several hydrocarbon mixtures as well as elements such as lithium. This water is extracted along with crude oil and gas, and also contains various fluids used during processing such as fracking fluids, cementing fluids drilling fluids etc. and is called produced water (PW).^96^ It is estimated that with every 1 barrel of oil produced, 3 barrels of wastewater are generated, though this can be up to 9–10 barrels for mature oilfields.

Besides high concentrations of ions such as sodium, calcium, magnesium and chlorides among others, PW contains a variety of impurities both organic (benzene, toluene, phenols, aromatic hydrocarbons, polyaromatic hydrocarbons etc.) and inorganic (heavy metal ions, radioisotopes, suspended solids etc.), and additionally there are Fe and SO_4_^2–^ reducing bacteria that generate hydrogen sulfide. These characteristics vary significantly based on the local geology, reservoir age and brine composition, and processing chemicals can be present such as corrosion inhibitors, biocides, defoamers and emulsion breakers may be present, as well as chemicals used for oil–water separation such as coagulants and flocculants.^97^ Currently, this brine is typically reinjected into the ground after gravity separation, but there have been growing concerns about seismic activity linked to reinjection wells as well fears of pollution of nearby groundwater and freshwater sources.^98,99^

As mentioned in section 1, the concentration of lithium in this brine can range from 100 to 500 mg/L, though it is typically closer to the lower end of the estimate.^27^ This means that evaporitic process for lithium recovery is not feasible, and furthermore the produced water is not permitted to be exposed to air for too long. Hence if lithium recovery is to be undertaken from PW, alternate DLE technologies will need to be implemented. In a traditional PW treatment strategy, a physical treatment based on gravity separation, density (liquid–liquid hydrocyclones etc.) or adsorption can be used to remove suspended solids and large portions of oil from the PW. A chemical treatment with precipitation or advanced oxidation processes (AOPs) to remove heavy metals and organics, followed by tertiary treatment to remove salts and biological waste is carried out.

Membranes can be applied at different stages for the demineralization and deoiling prior to resource recovery.^100^ Microfiltration (MF) and UF have been studied in the removal of suspended solids, heavy metals and crude oil in water, and have shown high levels of removal, particularly when an efficient pretreatment is utilized (see Figure 8). Indeed, there have been pilot scale tests to demonstrate the efficiency of UF membranes with produced water.^101^ Ceramic membranes have certain advantages over polymeric membranes in this application due to the potential for harsher cleaning strategies, though the high cost of these membranes and toxic waste generated in their cleaning could be limitations.^102,103^

**Figure 8:**
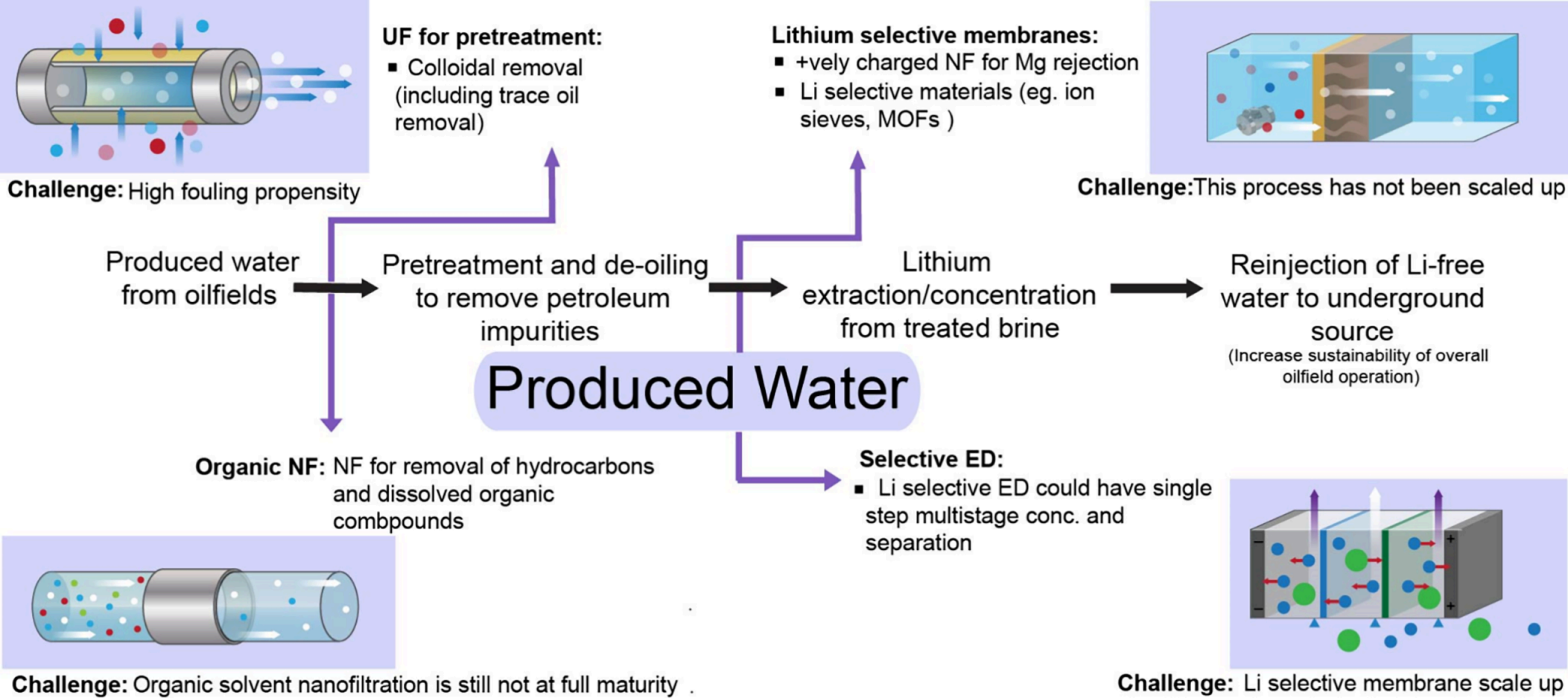
Process flow of conventional process for Li^+^ recovery from produced water and oilfield brine with membrane process replacements.

Membrane fouling is a significant concern in the treatment of PW, and a combination of physical treatment methods such as gravity separation and hydrocyclones can be used in pretreatment to reduce the suspended solids that reach the membrane, as well as optimization of membrane cleaning strategies such as backwashing and ultrasonic cleaning is necessary.

Following treatment by MF/UF, a secondary treatment with NF is required. NF can successfully remove dissolved organics (calculated by chemical oxygen demand, COD), TDS removal by targeting divalent ions like calcium and magnesium, sulfate, as well as oil and grease remnants that passed through the UF membrane.^104^ A positively charged NF membrane can be applied at this stage to further reject divalent ions and concentrate lithium, as seen in the case of other brines. This would require robust pretreatment as positively charged membranes are more prone to fouling.^105^ Finally, a lithium selective ED process can be applied here to recover lithium from the monovalent rich brine. The addition of an MLD/ZLD stage at different stages to treat the various reject streams can be implemented to ensure a clear water stream for reinjection or surface runoff.

Resource recovery from PW comes with many additional challenges due to the nature of the composition of the feed, but these are offset by some advantages. First, the infrastructure for water pumping already exists, and as in the case of seawater recovery, the lithium extraction infrastructure will simply augment the existing plant. Various water treatment processes also exist at the plant to treat PW to acceptable standards before reinjection, and a lithium extraction process can be implemented into this existing process chain as needed. The lithium recovered will also generate additional profits for the oil and gas industry. Finally, the treatment of the PW to a high level is increasingly being demanded by local communities where these plants are located, and resource recovery can go hand in hand with this strategy that needs to be implemented. Indeed, profitable lithium recovery can further incentivize oil and gas companies to comprehensively treat the PW (see Figures 9 **A and B**). Lithium recovery is already being studied at a preliminary level in Canada, USA and China.^96^

**Figure 9:**
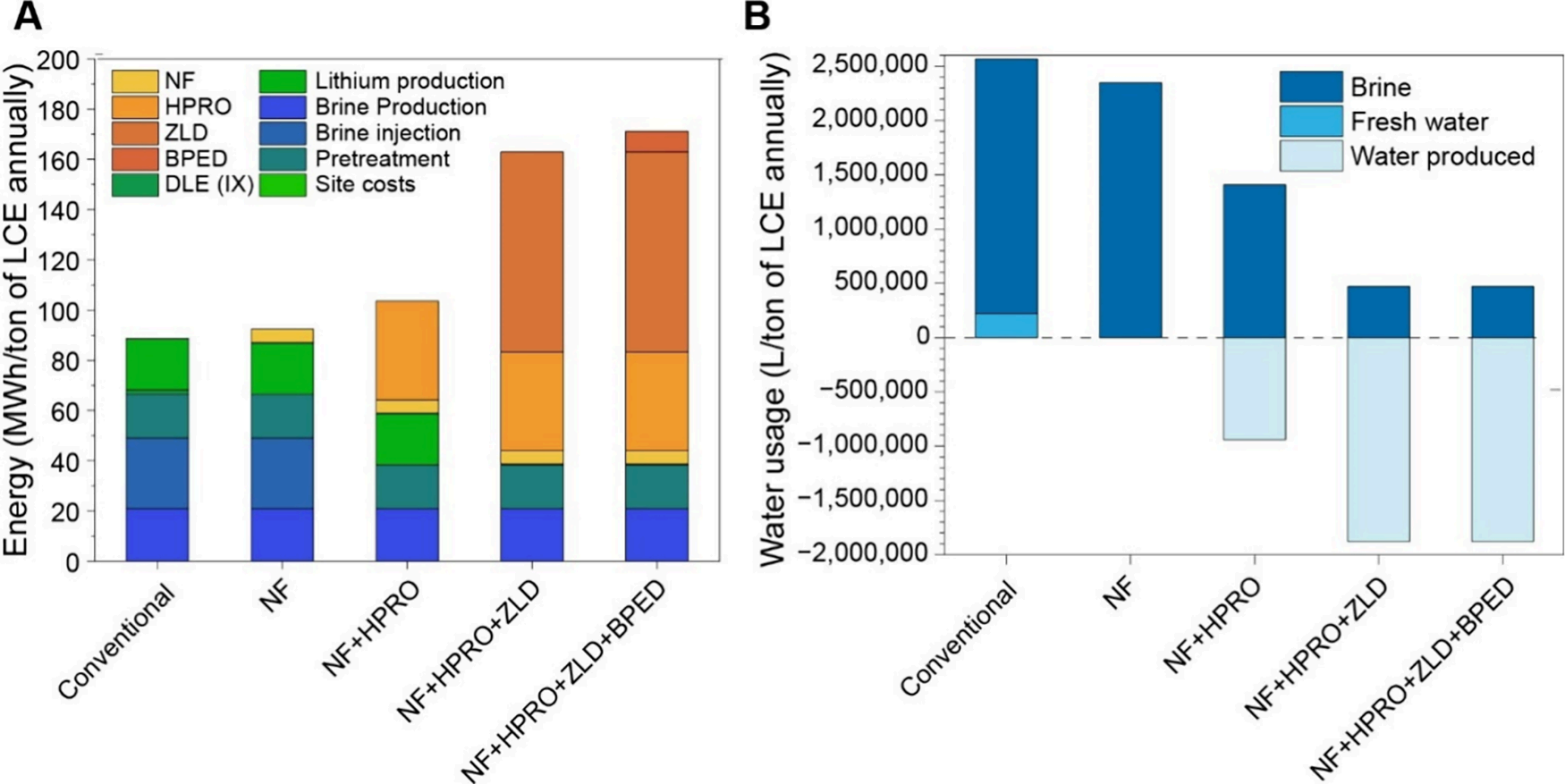
(A) Energy savings through replacement of conventional process with various membrane technologies. (B) Water savings through replacement of conventional process with membrane technologies. Calculation, sources, and values for panels A and B presented in the Supporting Information, Appendix C, Section 3 and includes Tables S12 and S13; NF = nanofiltration; HPRO = high-pressure reverse osmosis; ZLD = zero liquid discharge; BPED = bipolar membrane electrodialysis.

### 2.5 Lithium-Ion Battery (LIB) Waste

Approximately 464,000 tons of lithium ion battery waste is expected to be generated by 2025.^106^ A single Tesla Model S Long range battery can contain 350 kg of Li. Currently, around 95% of LiB waste generated is not recycled and ends up in landfills.^107^ This conflicts with the idea of LiBs being the cornerstone of a energy efficient and circular economy paradigm, and rapid scaling up of battery waste recycling is essential. Additionally, we must consider the amount of solid waste generated and water used in conventional lithium recovery, contrasted with the amount of lithium that is disposed of in batteries. Lithium is currently a limited resource with an increasing cost, and assuming there is no battery technology to replace LiBs soon, coupled with the ever-increasing demand, lithium recovery from LiBs is inevitable. Battery recycling has largely focused on cobalt as a strategic and scarce element.

Battery recycling begins with a preliminary treatment carried out in different ways, which is targeted toward separating the valuable cathode element from the unwanted parts such as metal casing, organic binder, electrode foils etc. This is followed by one of multiple strategies, pyrometallurgical treatment, hydrometallurgical treatment, or bioleaching. Pyrometallurgical method consists of heating the battery elements in a muffle furnace at high temperatures followed by reduction.^108^ This process generates toxic gases as well as making resource recovery challenging. Bioleaching is promising but faces many challenges toward scale-up currently. Hence, we will focus our attention on the hydrometallurgical process, which is more complex but allows greater possibility for resource recovery.

In essence, the hydrometallurgical process consists of solubilizing valuable elements in the cathode into a solution, followed by extraction.^109^ This is done by leaching through inorganic acids (highest efficiency), organic acids (less corrosion and toxic waste) and alkaline leaching.^108^ Following leaching, the leachate consists of the different elements in the battery such as Li, Co, Ni, Mn, Fe, Al etc. A combination of chemical precipitation, solvent extraction and electrochemical methods is utilized to extract the valuable elements selectively.^110^

Battery waste is a lithium source that can be targeted by membrane technologies in a number of interesting ways (see Figure 10). The ‘black mass’ that is formed after the preliminary treatment still contains a number of impurities which are passed into the leachate that can affect the recovery step.^111^ Pretreatment can be a vital step to prevent membrane damage in subsequent stages of selective membrane separations. A combination of MF/UF can be utilized to purify the feedstock and enhance the recovery step. Membrane processes can be applied to the leachate for recovery of lithium, and additionally other elements can be targeted as well. Electrodialysis has already gained attention, including regular ED and bipolar membrane ED.^112,113^ Recovery of Li^+^, as well as Co can be carried out, and a charged membrane employing Donnan exclusion can be applied here.^114^ Monovalent selective ED can also be applied in multiple stages to recover Li^+^ along with Co and Ni. The tolerance of ion exchange membranes to harsher acidic environments makes ED and other electrically driven processes such as membrane capacitive deionization (MCDI) of interest in battery recycling.^115^ The application of NF can also be strongly considered here, as the separation of monovalent and divalent ions can be carried out without hindrance from other similarly charged ions in this case, unlike the challenges encountered in the various types of brine sources.^116^ While TFC polyamide membranes have some tolerance to acidic environments, it will be necessary for long-term operation to neutralize the pH of the leachate before the feed is passed through the NF membranes. This can also assist with the precipitation of Fe and Al for easy removal. A positively charged NF membrane to employ Donnan exclusion can be ideal here as well.

**Figure 10:**
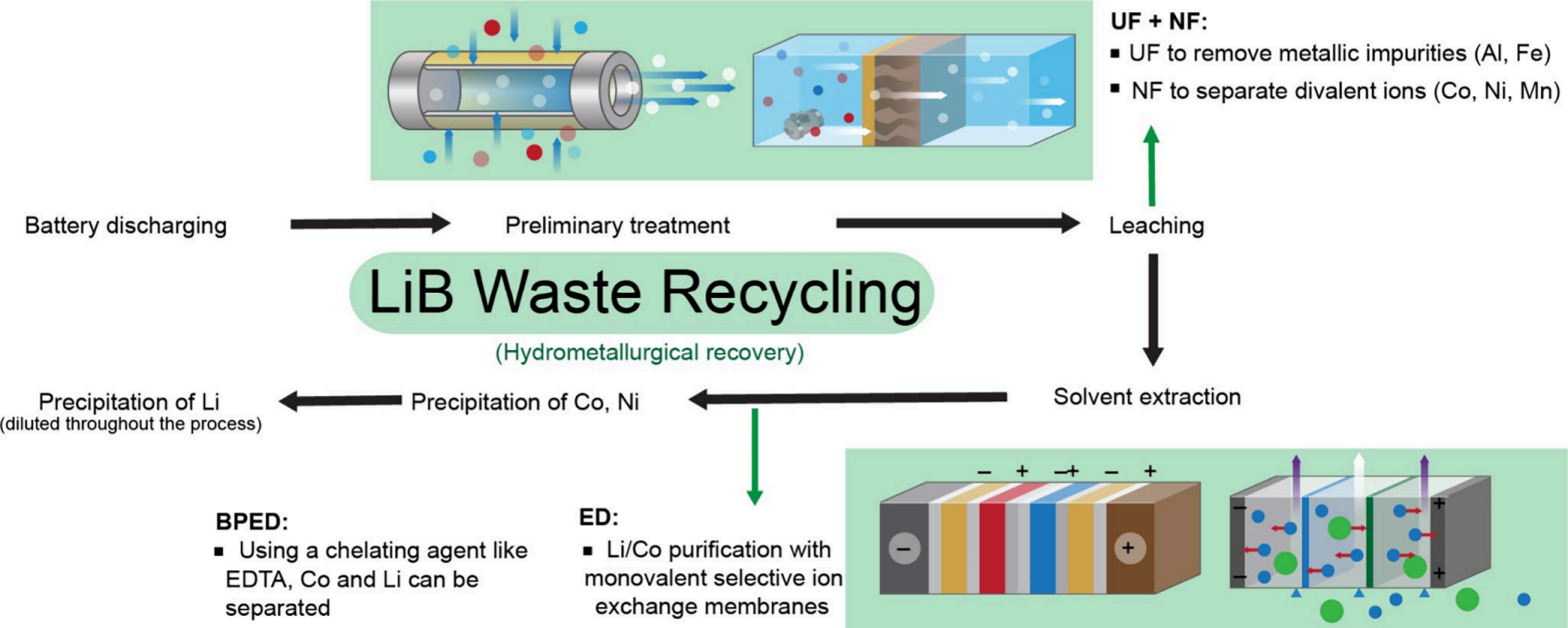
Simplified process flows for battery recycling commonly practiced (focusing on hydrometallurgical treatment) with lithium recovery.

Finally, membranes can play a useful role in turning the battery recovery process into a closed loop by helping in the recovery of the acid used for leaching, so that it can be reused.^117^ BPED can be used for the recovery of the acids used for leaching as well as conversion of LiOH as necessary.^118^ Another interesting membrane solution could be organic solvent nanofiltration (OSN) or reverse osmosis (OSRO) which can be utilized if organic solvents are used for the leaching step instead of acids.^119,120^ Membrane technologies can therefore play a crucial role in making battery recycling highly efficient.

## Section 3: Current State of Membrane Technologies for Lithium Recovery

In this section we evaluate the current state of membrane research as it pertains to lithium recovery and analyze its suitability for commercial applications. A host of membrane processes have been applied to lithium recovery over the years, with a range of chemistries applied to impart lithium selectivity to the membranes. An increased focus on lithium as a key resource for various technologies and batteries has consequently raised lithium prices, and this has coincided with a growing focus on ion–ion separations and membrane selectivity in the research community.^121,122^ Analyzing the burgeoning space of membranes for lithium recovery is thus a timely exercise.

### 3.1 Membrane Performance Analysis

We consider the major processes in lithium purification that can be targeted for replacement by membranes and the direction needed for implementation on a commercial scale. An overview of the process train for various lithium recovery processes and potential zones of application of membrane process is shown in Figure 11. A thorough bibliometric analysis was carried out to analyze and categorize membrane processes based on their targeted implementation in lithium recovery based on source and type of separation (Supporting Information, Appendix A). There are other processes besides the ones mentioned below, but our current analysis indicates that the following steps can benefit from replacement by membranes in terms of water and energy efficiency, as well as modular construction that minimizes land footprint.

**Figure 11:**
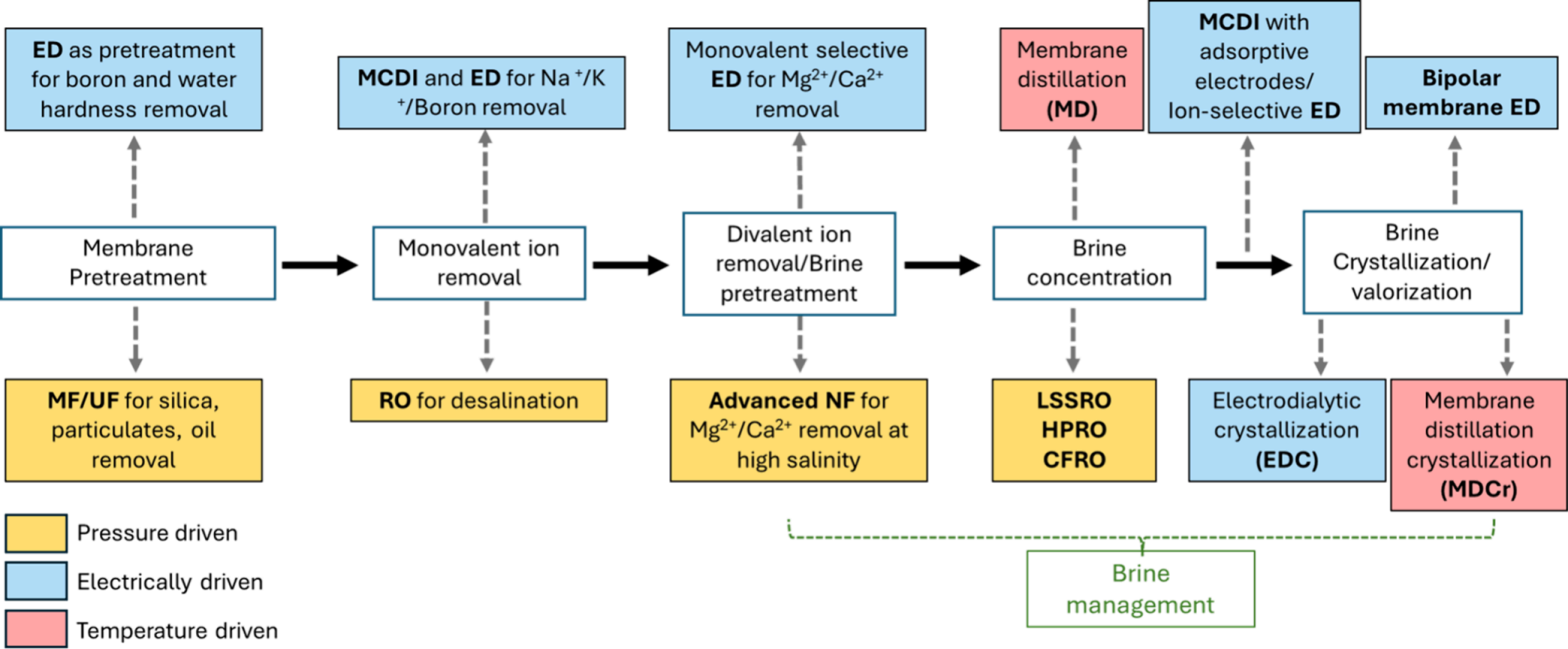
Sequence of stages in lithium recovery from multiple sources and potential membrane technologies that are applicable across the process trains.

The current and future implementation of pressure-driven, electrically driven and thermally driven membrane technologies is presented in Figure 11. Electrically driven membrane processes have wide applicability, but need to overcome challenges at the material, process and economic level. Thermal processes are largely applicable in the brine stage due to their high tolerance of feed salinity, while pressure-driven processes are either already implemented or close to commercial application.

### 3.2 Pretreatment

DLE technologies generally require more pretreatment than conventional evaporation ponds where impurities can settle over time. Pretreatment for removal of impurities such as silica, competing metals such as Fe and Al, and other divalent ions such as Ca^2+^ is necessary for DLE technologies to prevent fouling and scaling. Pretreatment can also help to improve efficiencies of technologies such as adsorption by reducing competing ions.^123^ While the use of pretreatment membranes specific to lithium recovery processes is scant in literature, membranes (MF, UF and NF) are used widely in pretreatment for desalination and water treatment.^124^ The feed characteristics vary greatly based on location and type for the various lithium recovery sources, and depending on the target impurities, the appropriate membrane process can be selected. The implementation of the right membrane process in pretreatment for foulant removal (silica, suspended solids, organics etc.) and reduction of scalants (Ca^2+^ and Mg^2+^ removal) will have a critical impact in a range of capital and operational cost factors.^125^ This includes membrane replacement age, chemicals and antiscalants used for fouling removal, corrosion minimization, higher lithium recovery and purity, energy efficiency through lower osmotic pressure requirements or voltage, and reduced number of process units.^125,126^ Membrane technologies are well proven in literature for pretreatment in a variety of industries.

Subsequently, even DLE technologies dependent on nonmembrane processes have already incorporated membranes for pretreatment. Tenova Advanced Technologies incorporates NF in its process flow for lithium recovery for removal of divalent ions prior to solvent extraction,^127^ which is being implemented at Clayton Valley, Nevada. Standard lithium plans to remove suspended solids from pumped up Smackover brine through UF membranes prior to adsorption processing.^128^ Eramet incorporates NF as well in its process flow following lithium extraction for the removal of divalent ions in its operation in Argentina.^129^ Membranes can have a significant role to play as a reliable and universal technology for pretreatment of lithium sources.

### 3.3 Li^+^/Mg^2+^ Separation

In this subsection we consider the application of membranes for Li^+^/Mg^2+^ separation. In particular, we address its potential for incorporation into the existing conventional process for replacing the liming process that removes the magnesium and sulfates in the feed prior to lithium carbonate production. Lithium selectivity values of membranes designed for NF are collected and plotted in Figure 12A versus the water permeability of the membrane. Subsequently, the selectivity is normalized for the magnesium to lithium ratio (MLR) utilized in the study using lithium purity (η_Li_) as a parameter,^62^ as shown in Figure 12B, where Li purity is defined as follows

ηLi=11+MLRSLi/Mg 1

where

**Figure 12:**
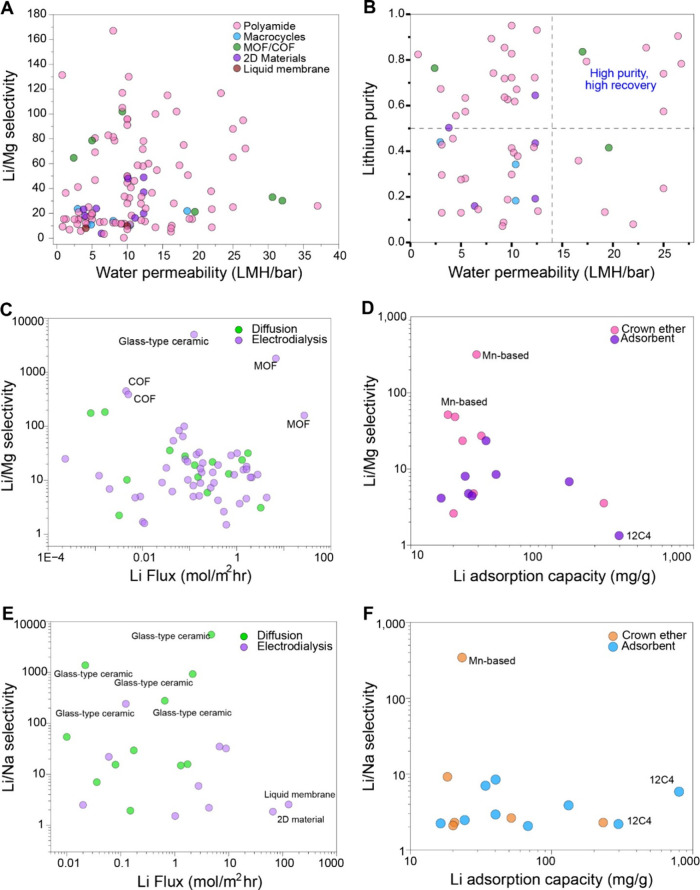
(A) Pressure driven NF membranes for Li^+^/Mg^2+^ separations classified as Li^+^/Mg^2+^selectivity versus Water permeability. Classes of membrane are shown in the figure legends; (B) lithium purity versus water permeability for membrane types shown in (A); (C) Li^+^/Mg^2+^selectivity versus Li^+^ flux for electrically driven and diffusion-based membranes (log scale); (D) Li^+^/Mg^2+^ selectivity versus adsorption capacity for adsorption-based membranes (log scale); (E) Li^+^/Na^+^ selectivity versus Li^+^ flux for electrically driven and diffusion-based membranes (log scale); (F) Li^+^/Na^+^ selectivity versus adsorption capacity for adsorption-based membranes (log scale). References and data for each point in this figure is presented in Tables S14–S19 in the Supporting Information, Appendix D.

MLR = magnesium to lithium ratio of feed solution

S_Li/Mg_ = Selectivity of lithium over magnesium of membrane

Water permeability is used in lieu of lithium recovery, which is often unavailable in literature for membrane coupon studies, as a stand in for productivity. Membranes were also classified based on the material used for solute selectivity.

Polyamide membranes utilize positive surface charge (for Donnan exclusion), or size sieving, or a combination of both mechanisms to reject divalent ions. Metal organic frameworks (MOF) materials such as ZIF-8 and UIO-66-NH2 are used to impart lithium selectivity, and so are macrocycles (primarily crown ethers 12C4, 14C4 and 15C5). Figure 12A shows that the majority of membranes that balance Li^+^/Mg^2+^selectivity with permeability are polyamide membranes. Among the strategies utilized to boost selectivity, polyamide membranes have used (i) positive-charge inducing strategies for increasing amine concentration on the surface including polyethylenimine-related coatings and modifications,^130,131^ (ii) reverse interfacial polymerization with amine polymerized over acyl chloride,^132^ and (iii) incorporation of other additives into the membrane surface.^133^ With selectivity normalized with MLR, the lithium purity graph shows a scatter spread of the different membranes for lithium recovery, with the top right bracket representing membranes that can perform high recovery while maintaining high purity of lithium in the permeate (Figure 12B). This bracket is largely populated by polyamide membranes, suggesting the effectiveness of these highly scalable membranes for Li^+^/Mg^2+^recovery.

Next, we categorize membrane performance for electrochemical and diffusion-based separations in Figure 12C, with Li^+^/Mg^2+^selectivity plotted versus Li^+^ flux. We also categorize the performance of adsorption-based membranes that incorporate either adsorbents (Al, Mn and Ti based) or crown ethers in Figure 12D. We observe that glass-type ceramic membranes^29,134^ show appreciable performance in electrical separations, with extremely high selectivity and flux. Further discussion on these materials is in the next subsection. Utilization of Covalent organic frameworks (COFs)-based membranes^135^ as well as MOFs such as HKUST-1 ^136^ lead to high Li^+^/Mg^2+^selectivity. In terms of adsorbents, Mn based membranes have high Li^+^ selectivity,^137^ which is in line with the performance of Mn based sorption processes for Li^+^ recovery (Figure 12D). It is worth noting that membrane performance tests for NF and ED differ in the type of feed used, with NF membrane studies utilizing more representative MLR, with ED studies so far typically utilizing a binary 1:1 solution for Li^+^/Mg^2+^separation. However, it is noted that existing membranes for electrodialysis can technically meet the requirements for Li^+^/Mg^2+^ separations in hypersaline brines. The utilization of ED for this step requires large energy consumption but this can be made more sustainable by combining the process with renewable energy sources which are typically abundant at sites with continental brine abundance possibly making ED a competitive technology.^69^

The rising demand of lithium in coming years requires implementation of technologies in the short term, and the high performance shown by easily scalable polyamide membranes makes them strong candidates for implementation. The volume of brine required to be processed demands membranes in large modules which are yet to be achieved with some of the more novel materials being tested. Further, while the increasing cost of lithium negates some of the cost of materials used for recovery, it may be challenging to incorporate some of the more expensive chemistries into the recovery process. This is particularly true in cases where material scale-up has not been demonstrated, when considering the current liming process is carried out successfully in conventional plants for large brine volumes that are processed.

Li^+^/Mg^2+^separation might already be successfully achieved by ED and NF at the level required in most saline brines, assuming some challenges are overcome. Both NF and ED rely on Donnan exclusion for Li/Mg separations. It is understood that multivalent ion rejection is reduced by high ionic strength of the feed solution in ED as Donnan exclusion is weakened^138^ and similar trends are seen in NF as well,^139,140^ which is a concern since lithium feedwaters generally have high ionic strength. In the case of ED, the scale-up and low-cost fabrication of ED remains a challenge.^141^ NF thus has a high TRL (Pilot scale^142^) since it is successfully implemented at pilot scale in industry already, while ED has a medium TRL (Experimental scale^142^) that is nevertheless within range of large scale commercialization in the medium term. Indeed, Northern Lithium has completed a 60 day trial in Durham County, UK for divalent ion removal in 3.5 million liters of brine,^143,144^ while EnergyX has deployed electrodialysis at pilot scale in Bolivia.^145,146^ Subsequently, the TRL of NF and ED can be estimated at 6–7 and 5–6 respectively. Additionally, there is also an emphasis on material development to make these membranes more tolerant to the typical challenging feedstocks for lithium sources, with the development of high charge density IEMs for ED as well as stable zwitterionic and polyelectrolyte coatings for NF.^147^ These innovative materials and additives will need to be rigorously tested for fouling removal and long-term stability before they are adapted in the lithium recovery process train.

### 3.4 Monovalent Ion Separations and Li^+^ Concentration from Dilute Sources

Separation of monovalent ions particularly between Na^+^, Li^+^ and K^+^ from each other is challenging due to their similar hydrated radii and charges, making size sieving and Donnan exclusion less effective. This is particularly true for pressure-driven processes. Sodium is a large component in most lithium sources, and the precipitation of Na^+^ is not feasible for sources such as seawater, produced water and geothermal brine. However, this separation is necessary for accessing most of the lithium sources needed to expand beyond current mineral and continental brine resources. Hence Li^+^/Na^+^ separation is a high-value target capable of transforming lithium production globally. In Figure 12 we also examine some of the membranes utilized in literature for Li^+^/Na^+^ separation. Figure 12E examines membranes used in electrically driven processes, as well as diffusion-based studies that can be applied for electrical separation. In Figure 12F we examine the effect of adsorbents and crown ethers for Li^+^/Na^+^ separation.

Monovalent ion separations is a field of great interest in separation science, and various innovative materials have been tested for Li^+^/Na^+^ separation. The most popular among these materials are lithium selective ligands such as crown ethers and MOFs/COFs, which use coordination chemistry to preferentially capture Li over other ions. These materials can either be embedded, coated or systematically aligned within a polymer matrix where the lithium is adsorbed onto the surface ligand followed by penetration through the polymer.^147,148^ These materials face significant challenges to scalability, such as uneven distribution in polymers, defect formation during embedding etc., which need to be overcome.^149^ Other challenges can arise with selectivity such as competing ions for site adsorption in complex mixtures, as well as high adhesion of lithium to the coordination site leading to reduced lithium flux.^147^

Biomimetic membranes depend on artificial channels that utilize the specific charge, hydration energy and ionic radius of lithium to selectively transport it across a polymer matrix. This transport is facilitated by specific functional groups along the channel, which also inhibit the transport of competing monovalent and divalent ions. A lithium-selective biomimetic membrane could allow for a highly selective, highly permeable membrane with a low energy cost and rapid, unhindered ion transport.^150,151^ However, the scalability of biomimetic membranes remains a challenge as the fabrication of these channels can be complex and time-consuming, while the membranes need to be tested for complex feedwaters with a challenging environment. 2D materials such as graphene oxide and MXenes are another class of materials that can be utilized for lithium separation. These materials can be precisely tuned for specific interlayer thickness, which leads to separation based on size exclusion.^152^ Further, functional groups present in the 2D layer can enhance separations based on electrostatic interactions that can promote or delay an ion’s passage through the interlayers.^153^ 2D materials are a promising class of membranes for ion separations, however their scalability remains a challenge. The formation of large scale 2D materials that are defect free has been elusive, which has made it difficult to translate lab scale successes.^154,155^ In addition, there can be a negative effect of complex and high ionic strength brines on the interlayer spacing and chemistry, leading to poor selectivity of the desired ions.^147^

The success of glass-type ceramics including Li_1.5_Al_0.5_Ti_1.5_(PO4)_3_ (LATP)^156^ and Li_0.33_La_0.56_TiO_3_ (LLTO)^29^ using intercalation-deintercalation method based on the size of Li^+^ ions, is very notable for this separation. While these membranes have made Li^+^/Na^+^ separation and Li^+^ recovery feasible, the challenges related to the scale-up of these glass-type membranes remains a question. Nevertheless, these membranes demonstrate the monovalent selectivity that can be required from sources with a low Li^+^/Na^+^ ratio in the feed.

In terms of adsorbents, we note the success of Mn-based adsorbent membranes in terms of selectivity, and 12C4 crown ether based adsorbent membranes in terms of Li^+^ flux again.^157,158^ The development and scale-up of these membranes would be useful as well, for nonseawater feed solutions with equivalent concentrations of monovalent ions, such as during polishing steps in lithium recovery to eliminate low concentrations of monovalent ions present. Boron is a monovalent impurity that has not yet received much attention in literature, however its presence in the feed can hamper the formation of battery-grade lithium products. Currently it is removed through solvent extraction in commercial plants where it is present in undesirable quantities. However, its removal through some of the membranes with lower Li/monovalent selectivity could be advantageous and minimize the use of chemicals utilized in the solvent extraction stage for Boron.

Membranes can also be utilized to increase the concentrations of the lithium in the feed solution, prior to carbonate treatment and crystallization. Here we note that commercial DLE technologies are implementing membranes for this process currently. Eramet utilizes RO for the concentration of the feed solution and removal of water prior to purification and recovery.^129^ Koch separation solutions also utilize RO for brine concentration prior to evaporation and crystallization. This step can be utilized to minimize costs for evaporation of the feed, as well as recovering water to increase the overall sustainability of the entire process.

### 3.5 Brine Management and Treatment

#### Pressure-Driven Membrane Technologies for Brine Management

As stated earlier, the ability to treat brine can be another transformative step in lithium recovery. The combination of brine treatment with lithium selectivity is currently only a theoretical possibility using membrane technology. If implemented, this would allow the elimination of ponds entirely from continental brines, as well as significantly increase the feasibility of geothermal, seawater and produced water lithium production. Various membrane technologies such as osmotically assisted reverse osmosis (OARO),^159^ closed circuit RO (CCRO), counter-flow RO (CFRO), high pressure RO (HPRO), low salt rejection RO (LSRRO) have been proposed and studied for brine management, as they can draw upon decades on technical know-how from the RO industry. In the case of HPRO, Anvari et al. recently discuss the feasibility of ultrahigh pressure RO at up to 400 bar to manage brines with 250,000 ppm concentrations, which is close to what is required for lithium-rich brines.^79^ This will require design considerations for the membrane module, including spacers, housing etc. as well as the membrane, with pressure driven membrane compaction becoming a severe issue.^79,160^ Nevertheless, the target is an engineering challenge that might be more achievable than very high Li^+^ selective membranes on a large scale.

Other pressure-driven membrane technologies that have gained prominence for brine management, such as counter-flow RO (CFRO) and low salt rejection RO (LSRRO), among others. These technologies depend on reducing the osmotic pressure of the feed by increasing the salt concentration in the permeate chamber. This can be done through a range of operating and staging methods, which are explained elsewhere.^161^ These technologies can utilize conventional RO membranes and modules (with some modifications in the case of CFRO) since they can be operated at regular RO pressures. The energy efficiency of these various competing brine management technologies is a matter of ongoing research which highlights the increasing importance that is being attributed to brine management by the membrane research community.^162^ These membrane processes are expected to reduce the energy requirement of ZLD compared to the current thermal-based methods significantly, which should improve the overall plant economics and efficiency of brine management in lithium recovery. The management of brine after lithium recovery could also benefit significantly from minimizing water wastage that is caused by reinjection of chemically polluted brine back into the lithium source.

#### Membrane Distillation/Crystallization

Membrane distillation/crystallization (MDCr) is another technology that can be utilized for brine management. With the application of a temperature gradient, the feed can be concentrated with the removal of water through the hydrophobic membrane until the salt concentrations of most of the other ions is close to supersaturation, which can be subsequently crystallized, while recovering pure water.^86,163^ Membrane distillation (MD) also has the benefit of being able to tolerate extremely high salinities compared to pressure and electrically driven membrane processes, which is a unique benefit when applied to such concentrated brine solutions.^164^ The drawbacks of this process are the requirement of temperature input, however there is the potential of waste heat availability in resources such as produced water near an oilfield, high temperature geothermal brines, as well as the potential to combine with solar technologies.^165,166^ Some drawbacks are pore-wetting and low flux through the membrane for this process, however it can still result in significant time reduction compared to the year-long process in an evaporation pond, with a much smaller footprint.^167^ There are also alternate electrically driven processes for brine reduction and crystallization such as electrodialytic crystallization that could gain prominence in the future.^168^

### 3.6 LiOH Production

As stated earlier, LiOH is increasingly the more desirable form of lithium in batteries, while Li_2_CO_3_ and LiCl are the natural end-products in conventional pond technology. The demand of LiOH is expected to more than double by 2030 as the desirable form of lithium.^169^ Conventional conversion to LiOH through reaction with caustic soda has multiple drawbacks, such as requirement of chemicals, equipment and large footprint, as well as time-consuming reaction stages.^169,170^ Bipolar membrane electrodialysis (BPED) is a technology that employs bipolar membranes (consisting of both an anion exchange layer and cation exchange layer) in combination with a series of cation and anion exchange membranes. BPED presents an attractive pathway to generate LiOH from Li_2_CO_3_ or LiCl while minimizing chemicals and infrastructure needs. We present the timeline and future development required for bipolar membranes to be able to scale up and successfully find commercial application in the lithium recovery process in Figure 13.

**Figure 13:**
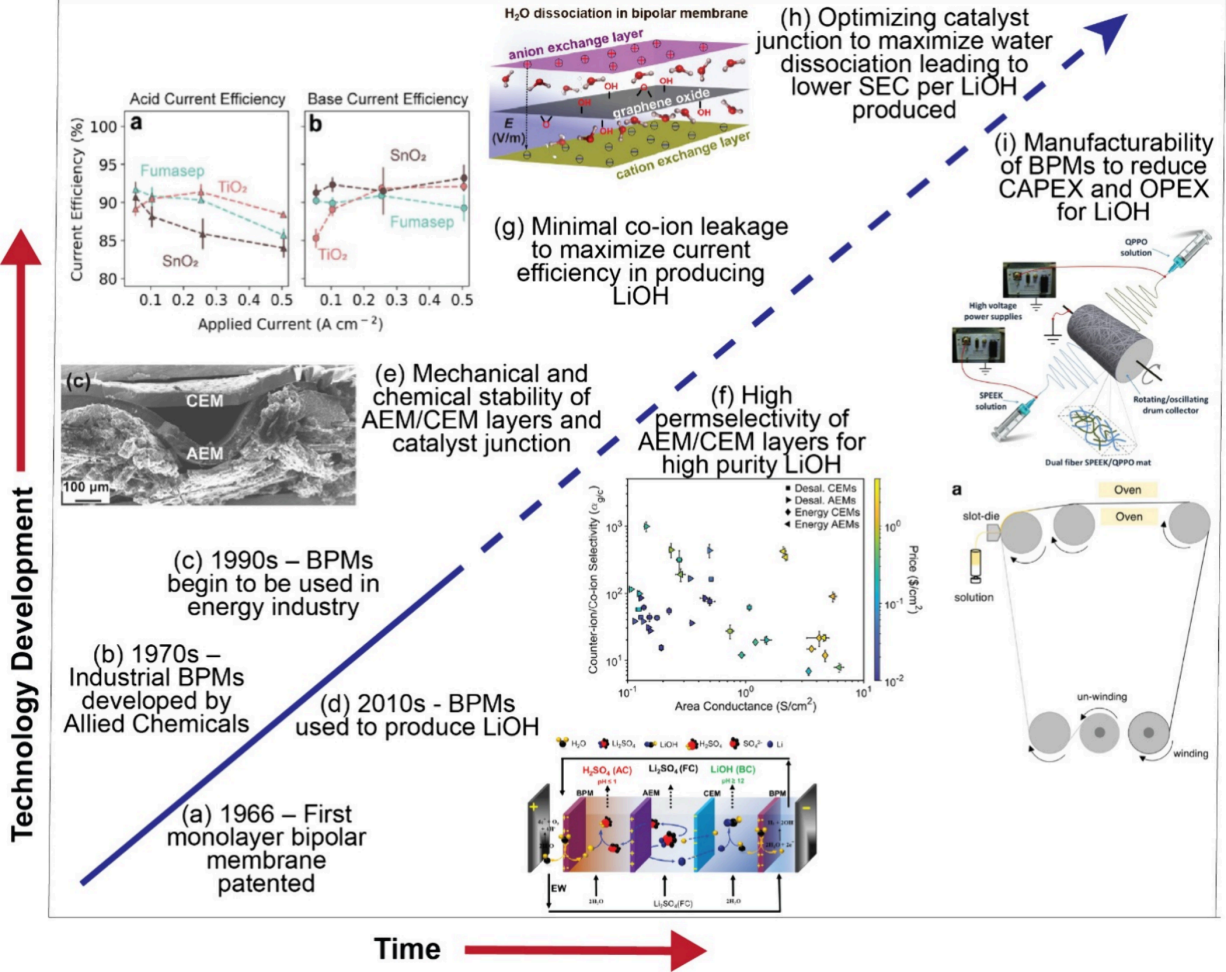
Timeline and roadmap for development of bipolar membranes and future requirements to advance technology for commercial lithium recovery for LiOH production. (a-c) adapted partially from ref 171 (d) bipolar membrane electrodialysis for production of LiOH. Reproduced with permission.^172^ Copyright 2026, American Chemical Society; (e) BPM delamination post operation. Cropped from Figure 3 in ref 173, licensed under CC-BY 4.0; (f) Permselectivity of commercial AEMs and CEMs versus area conductance and price ranges. Reproduced with permission.^138^ Copyright 2023, American Chemical Society; (g) current efficiency measurements for BPED for different membranes. Reproduced with permission.^174^ Copyright 2025, American Chemical Society; (h) Catalyst junction in bipolar membrane for water dissociation. Reproduced with permission.^175^ Copyright 2023, American Chemical Society; (i) manufacturing technologies that can be used for BPM fabrication such as electrospinning and roll-to-roll manufacturing. Reproduced with permission.^176,177^ Copyright 2022, American Chemical Society and Copyright 2018, American Chemical Society (straight line = historical milestones; dashed line = future milestones).

Some of the challenges related to BPED are related to the permselectivity of the membrane layers, which allows for salt leakage and Cl- and OH- transport into the opposite chamber at high feed concentrations, thus minimizing the final purity of the LiOH and reducing the concentration of the feed Li_2_CO_3_ that can be treated. The membranes need to be able to balance lower resistance with the increased permselectivity to minimize energy costs required, as well as increased efficiency in water dissociation. Conventional LiOH production from brines amounts to a SEC of 6.83 kWh/kg, while in BPED it is estimated that a SEC of 4 kWh/kg could be possible.^169,178^ It is also important to develop BPED capable of delivering high concentrations of LiOH that are close to the saturation level, to minimize the cost of evaporation. Recent studies have shown that BPED in combination with other electrochemical separation processes might lead to profitable recovery of high-purity LiOH compared to other processes.^179^ Hence, the design of new and improved bipolar membranes can make this potential application a reality.

While we have mentioned various technologies in this section, there are numerous gaps and limitations that need to be overcome in each case before the membrane technology can be deployed for critical mineral recovery at a commercial scale on-site. The table below (Table 3) presents some of these areas of research that can reach a high TRL. This is not an exhaustive list and there are other gaps that need to be addressed aside from the ones mentioned but will illustrate the challenges present in each case that hinder commercialization and need to be addressed.

**Table 3:** Description of Membrane Technologies at Different Stages of Lithium Recovery and the Potential Research Focus Areas to Drive the Technologies to Commercialization^a^

no.	stage	technology	research areas
1.	pretreatment	UF	1. silica removal studies to reduce fouling from high Si content in brines such as geothermal sources
			2. tolerate harsher feeds with contaminants such as oil residues, colloidal particles etc. that can cause physical and chemical degradation of membranes
		NF	charged membranes for high Mg/Ca rejection and high Li passage based on Donnan exclusion
		ED	development of commercial monovalent selective CEMs for hardness removal in feeds
2.	monovalent ion separation	RO	1. incorporation of lithium selective ligands to bind to Li^+^ in feeds for high purity
			2. higher recovery rates than traditional desalination (50%) for increased lithium recovery rates
		ED	1. ion-selective IEMs with Li^+^ selectivity over other prominent monovalent ions
			2. tolerance to high feed concentrations while maintaining high selectivity
		MCDI	development of lithium-selective adsorbents for electrodes with high regeneration capacity for stable, long-term use
3.	divalent ion separation	NF	divalent rejection at high ionic strength feeds since most brines have high magnesium-to-lithium raios
		ED	Donnan exclusion maintained at high ionic strength brine for divalent rejection with improved polymer chemistry and charge density
4.	brine management	LSRRO	1. membranes with tunable salt and water permeabilities that can be used for varying feed concentrations
			2. optimum membrane module organization for superior specific energy consumption (SEC) while achieving desired brine concentration
		HPRO	1. novel spacer development for minimal concentration polarization and overcoming mass transfer limitations
			2. high-pressure tolerant modules and elements to operate safely at high feed pressures (>120 bar)
		CFRO	1. antiscaling strategies and membrane coatings to avoid scaling as brines approach saturation limits for different salts
			2. stable RO membranes for a range of salt concentrations that can maintain rejection rates with increasing ionic strength of feed
5.	brine valorization	EDC	1. antifouling ion-exchange membranes to avoid scaling at high concentrations
			2. minimizing impurities from precipitated salts and achieving high recovery of salt from brines
		BPED	1. increased purity with minimal co-ion leakage to achieve desired purity of final salt products
			2. increased current efficiency and operability at high current density
		MDCr	1. increased permeate flux and reduced membrane wetting to operate MD for long durations
			2. superior understanding of salt nucleation and antiscaling strategies for crystallization stage

a UF = ultrafiltration; NF = nanofiltration; ED = electrodialysis; RO = reverse osmosis; MCDI = membrane capacitive deionization; LSRRO = low salt rejection reverse osmosis; HPRO = high-pressure reverse osmosis; CFRO = counter-flow reverse osmosis; EDC = electrodialytic crystallization; BPED = bipolar membrane electrodialysis; MDCr = membrane distillation crystallization.

## 4 Future Recommendations

Based on our analysis of literature for lithium separation, we believe some of the following salient points would be worth considering in this burgeoning sphere of membrane research:

1. Membrane development for Li^+^/Mg^2+^ separation is perhaps the separation that is closest to commercialization. Membrane research in this sphere might not benefit significantly from complex and expensive chemistry applications, with conventional materials for NF and ED starting to approach the desired performance levels. The challenge remains in taking lab-scale successes to the pilot scale with surface coatings and modifications of NF membranes that enhance divalent rejection and can be stable over long durations. The efficient application of ED at high ionic strength is also a target that can transform this separation step.
2. Monovalent separation through membranes remains a major challenge, and novel chemistries need to be further explored. Significant efforts are needed to develop next generation ion separation tools through biomimetic membranes, MOF/COFs, crown ethers, and 2D materials. Rapid material screening and testing is needed to identify the most promising avenues, and the potential for eventual scale-up needs to be kept in mind. The successful development of large scale monovalent selective membranes will open up many new lithium sources for recovery and be transformative for membrane application in critical mineral recovery.
3. Brine management, MLD and ZLD are largely ignored for lithium recovery, even though they represent a substantial portion of the energy and water challenges facing lithium extraction. Developing processes for brine management as well as techno-economic analyses that evaluate the impact of brine on costs and energy requirements are needed, not merely freshwater usage. Membranes developed for desalination brine treatment can find applications here as well, and membrane processes such as HPRO, CFRO, and LSRRO need to be studied specifically in the context of lithium-rich brines, since these are even more challenging than RO brine from desalination plants.
4. The impact of membranes on less studied and implemented steps such as pretreatment, concentration, and crystallization need to be studied. There is room for niche technologies such as membrane distillation for the right feed source. Membrane technologies can fill many niche roles alongside other DLE technologies, as well as in conventional evaporation ponds. Technologies such as BPED which can carry out chemical conversion, or MDCr that can perform crystallization, can fulfill unique roles that have a significant impact on the over process efficiency.

## Supplementary Material



## Notes

The Supporting Information consists of: The Supporting Information is available free of charge at https://pubs.acs.org/doi/10.1021/acsestengg.5c00997.

Appendix A: Literature survey for bibliometric analysis on membranes for lithium recovery; Appendix B: data and references used for lithium demand and supply projections, and lithium sources and their concentrations; Appendix C: energy and water usage data with references for continental brine, geothermal brine and produced water; Appendix D: references for data used for figures of merit for lithium selective membranes from literature (PDF)
