# Supplementary material for: Membranes for Lithium Recovery From Conventional and Unconventional Sources
Source: ACS ES T Eng. 2026 Apr 7;6(5):1402–26. doi: 10.1021/acsestengg.5c00997 (PMC13162263; doi:10.1021/acsestengg.5c00997)
Supplement: Supplementary file 1 [file ee5c00997_si_001.pdf]

Membranes for lithium recovery from conventional and unconventional sources

Nurshaun Sreedhar[1,2], Rebecca Lee[2,3], Sreejith Appukuttan[2], Hariswaran Sitaraman[2], Jason DesVeaux[2], Gary Grim[2], Mou Paul[2], Manish Kumar[1,3*], Abhishek Roy[2*]

[1] Maseeh Department of Civil, Architectural and Environmental Engineering, The University of Texas at Austin, Austin, Texas, 78712, USA

[2] National Laboratory of the Rockies, Golden, Colorado, 80401, USA

[3] McKetta Department of Chemical Engineering, The University of Texas at Austin, Austin, Texas, 78712, USA

Appendix A: Bibliometric status

An exhaustive search for literature published in the last decade on membranes related to lithium selectivity was carried out on Scopus. The resulting data is classified and tabulated, with the results present in Figure S1. The rapid rise in papers for this application over the past 5 years shows rising interest in this sphere. Papers are classified based on the membrane process applied (Figure S1 A, C) and the material property employed to impart lithium selectivity (Figure S1 B, D) and some observations can be made regarding the direction of research. Nanofiltration has grown significantly as an avenue of research in this sphere and constitutes a large fraction of the overall research undertaken. This is due to the success of NF charged membranes for application in magnesium rich brines. Membranes for electrical separation through ED have been prevalent for Li recovery while membrane capacitive deionization (MCDI) could be growing in popularity. Observing the material chemistry, we note that the largest number of membranes deployed depend on the pore size and surface charge for separation. MOF/COF based membranes and 2D materials are also being applied to lithium recovery, with the rising costs of lithium providing potential justification for the use of more expensive materials in membranes. Liquid membranes on the other hand have not grown significantly in terms of implementation.

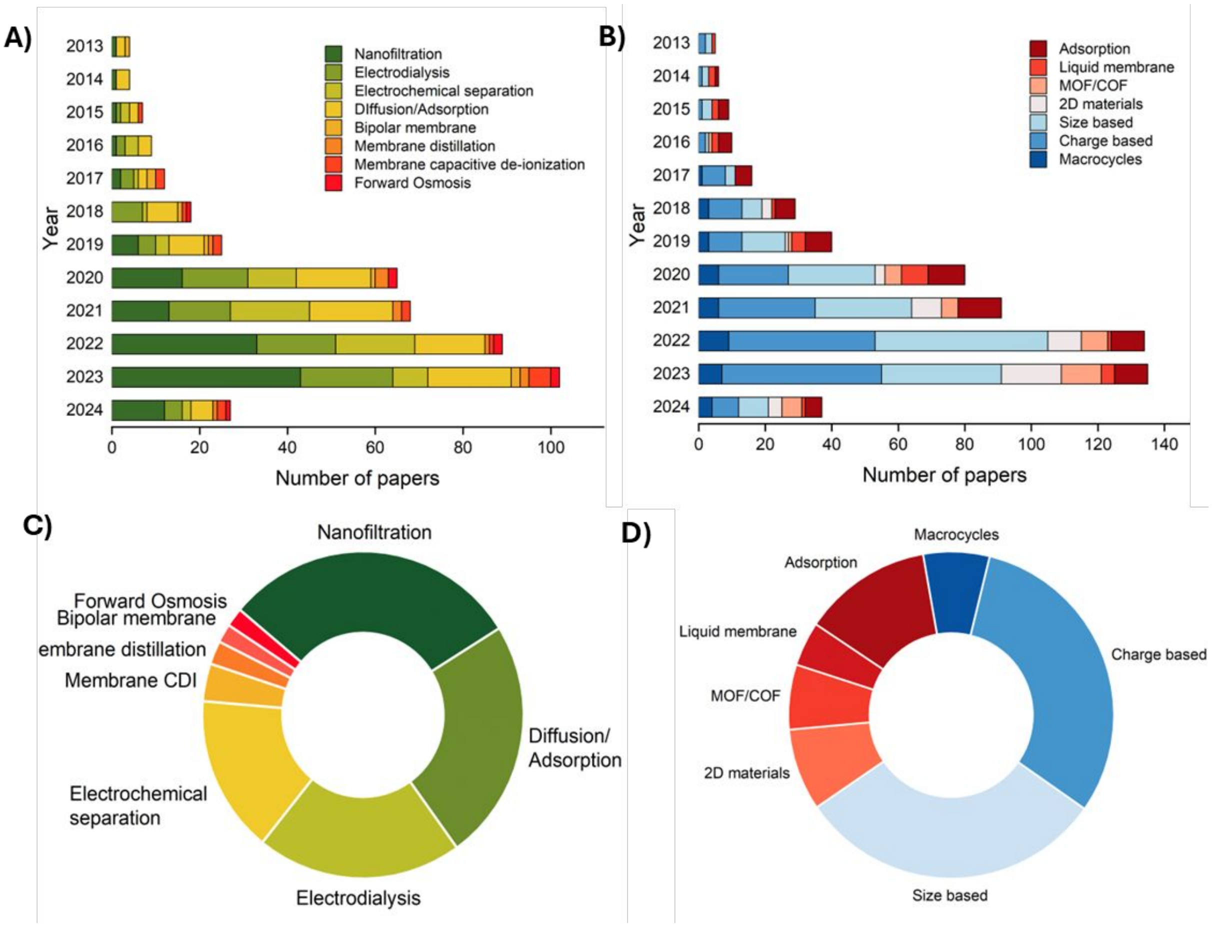

Figure S1: A & C) Breakdown of membrane processes employed for lithium selectivity from 2013-2024; B & D) Breakdown of membrane chemistry employed for lithium selectivity from 2013-2024; (2024 updated in March 2024)

A further breakdown of membrane targets for separation and the feeds utilized in research give us more insight into the direction followed (Figure S2). Hence the largest portion of Li research with membranes is focused on NF and ED with charged membranes that carry out Li/Mg separation (Figure S2 A). The majority of papers focus on divalent/monovalent separation with continental and geothermal brines in mind (Figure S2 B). Focus on monovalent separation and seawater is lower, and battery waste recycling is still a small part of the focus for Li recovery. Produced water is almost completely missing from literature, however membranes developed for geothermal brine can be applied to produced water in most cases with the right pretreatment to remove the oil residues.

A breakdown of processes applied for battery waste recovery shows that NF makes way for electrically driven separations, with ED, BPED and MCDI being popular (Fig. S2 C). High TDS, that can drive up energy demands in ED in saline streams, is not a challenge in battery waste and thus may be a reason for the high interest in this area. On the other hand, most TFC polyamide

membranes struggle with the acidic or alkaline feeds derived from battery waste. pH neutralization as well as utilizing more robust materials such as ceramics can provide a pathway for NF to find more ground in battery waste recycling.

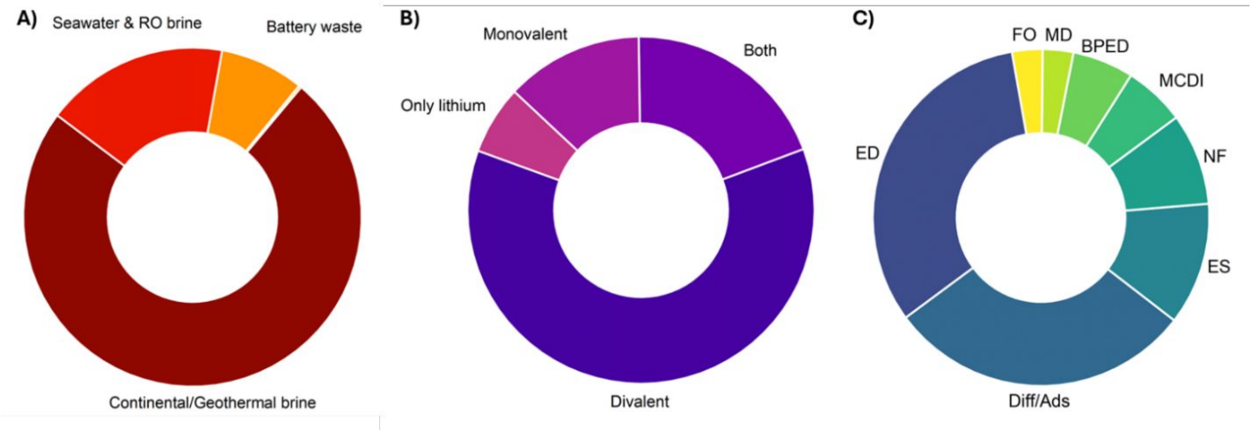

Figure S2: A) Feed sources targeted in literature; B) separation targets for membranes analyzed; C) membrane processes applied for lithium recovery from battery waste. (2013-2024)

Appendix B: Collected data on Lithium sources and DLE performance

Table S1: Global demand and supply forecast for lithium

No.	Year	Demand	Supply
1	2020	310	-
2	2021	508	-
3	2022	690	634
4	2023	917	862
5	2024	1072	1065
6	2025	1257	1320
7	2026	1433	1542
8	2027	1628	1709
9	2028	1861	1887
10	2029	2130	2000
11	2030	2453	2142
12	2031	2688	-
13	2032	2933	-
14	2033	3182	-
15	2034	3477	-
16	2035	3829	-

Data for lithium supply [1] and lithium demand [2]

Table S2: Total volume of major global lithium resources from different sources

No.	Source type	Name	Location	Li (kT)	Ref.
1	Pegmatite	Greenbushes	Australia	943	[3]
2	Pegmatite	Wodgina	Australia	826	[3]
3	Pegmatite	Earl Grey	Australia	658	[3]
4	Pegmatite	Pilgangoora	Australia	293	[3]
5	Pegmatite	Groto do Cirilo	Brazil	220	[3]
6	Continental brine	Atacama	Chile	6300	[3]
7	Continental brine	Uyuni	Bolivia	3600	[3]
8	Continental brine	Zhabuye	China	1500	[3]
9	Continental brine	Centenario	Argentina	921	[3]
10	Continental brine	Hombre Muerto	Argentina	835	[3]
11	Clay	Sonora	Mexico	845	[3]
12	Clay	Thacker Pass	USA	582	[3]
13	Clay	Rhyolite Ridge	USA	209	[3]
14	Geothermal brine	Salton Sea	USA	2000	[3]
15	Geothermal brine	Upper Rhine Graben	Germany	1182	[4,5]
16	Oilfield brine/Produced water	Smackover brine	USA	750	[6]

17	Oilfield brine/Produced water	Foxcreek	Canada	362	[6,7]
18	Battery waste	China	China	500	[8]
19	Battery waste	USA	USA	200	[8]
20	Battery waste	EU	EU	200	[8]

Table S3: Concentration and total volume of different major lithium sources

No.	Source type	Concentration (wt.%)	Total lithium (kT)	Ref.
1	Minerals	1.091	943	[3]
2	Clay	0.229	845	[3]
3	Continental brine	0.184	6300	[3]
4	Geothermal brine	0.04	2000	[3]
5	Seawater	0.00002	180000000	[9]
6	Produced water	0.03	750	[6]
7	Battery waste	6	500	[8]

Table S4: Water usage for lithium recovery from different sources

No.	Source type	Freshwater (m^3/ton of LCE)	Brine (m^3/ton of LCE)	Ref.
1	Mining	170	0	[10]
2	Continental brine	50	800	[11]
3	Geothermal brine	247	1000	[12]
4	Produced water	218	2000	[13]
5	Battery waste	45.9	0	[14,15]

Table S5: Large volumes of different DLE technologies

No.	Technology	Plant used	Size (mgd)	Ref.
1	Adsorption	Sweeney Water Treatment Plant, NC, USA	44	[16,17]
2	Solvent extraction	Soybean oil extractor, Argentina	23.77	[18,19]
3	Ion exchange	Orange county, PFAS treatment, USA	25	[20]
4	Membranes	Rabigh desal plant, KSA	159	[21]

Appendix C: Water and energy calculations

1. **Continental brine**

Technical report used: DEFINITIVE FEASIBILITY STUDY OF MSB BLANCO LITHIUM CARBONATE PROJECT [22]

Freshwater flow = 35 L/s

Brine flow = 158 L/s

Total electrical energy = 15 MW

Thermal energy cost = 658 US$/ton of LCE

Total energy cost annually = 13,160,000 US$/year

Gallons of diesel used per year = 3463157 gallons/year, or 395.34 gallons/hr (based on EIA price [23])

Thermal energy of diesel = 57300.949 MJ/hr, or 15.91 MW (based on energy conversion from EIA [24])

Table S6: Energy calculations from cost fractions

Process	Consumed energy (US$/ ton of LCE)	Energy fraction	Energy used (MW/ton of LCE)
Wells	5	0.017921	0.2688172
transfer ponds	34	0.121864	1.82795699
Evaporation ponds	23	0.082437	1.23655914
Salt removal plant	157	0.562724	8.44086022
Li2CO3 plant	38	0.136201	2.04301075
Camp	10	0.035842	0.53763441
Truck shop	6	0.021505	0.32258065
Transmission losses	6	0.021505	0.32258065
Total	279	-	-

Table S7: Energy breakdown for conventional process and membrane processes

Process	Thermal	Wells and transfer ponds	Evaporation ponds	Salt removal plant	Li2CO3 plant	Plant operations	NF	HPRO	ZLD	BPED
Conventional	15.91	2.1	1.24	8.44	2.04	1.18	0	0	0	0
NF	0	2.1	1.24	0	2.04	1.18	0.57	0	0	0

NF+HPRO	0	2.1	0	0	2.04	1.18	0.57	4.1	0	0
NF+HPRO+ZLD	0	2.1	0	0	0	1.18	0.57	4.1	12.64	0
NF+HPRO+ZLD +BPED	0	2.1	0	0	0	1.18	0.57	4.1	12.64	8.2

Table S8: SEC for different membrane processes

Process	Specific energy consumption (SEC)	Units	Ref.
Nanofiltration	1	kwh/m3	[25]
High pressure RO	9.18	kwh/m3	[26,27]
Brine concentration	24	kwh/m3	[27]
Brine crystallization	66	kwh/m3	[27]
Bipolar ED	4	Kwh/kg of LiOH	[28]

Table S9: Water usage for conventional and membrane processes

Process	Fresh water (L/s)	Brine (L/s)	Freshwater (L/year.perLCE)	Brine (L/year.perLCE)	Water produced (L/year.perLCE)
Conventional	35	158	55188	249134.4	0
NF	0	158	0	249134.4	0
NF+HPRO	0	68	0	107222.4	141912
NF+HPRO+ZLD	0	31.6	0	49826.88	199307.5
NF+HPRO+ZLD+BPED	0	31.6	0	49826.88	199307.5

2. Geothermal brine

Technical reports used:

a. Vulcan Zero Carbon Lithium™ Project Phase One DFS results [29]
b. Positive Zero Carbon Lithium™ Project Bridging Study Results [30]
c. BRIDGING ENGINEERING STUDY RESULTS, PHASE ONE FINANCING LAUNCH EDITION [31]

Freshwater flow = 138 L/s (500 m3/h assumed for the adsorption process based on analysis by Vera et al. [11])

Brine flow = 950 L/s

Total energy = 58 MW

Table S10: Energy breakdown for conventional process and membrane processes

Process	Wells and piping	Lithium extraction plant	Central lithium plant	NF	HPRO	ZLD	BPED
Conventional	21	15	22	0	0	0	0
NF	21	0	22	3.4	0	0	0
NF+HPRO	21	0	22	3.4	25	0	0
NF+HPRO+ZLD	21	0	22	3.4	25	50	0
NF+HPRO+ZLD+BPED	21	0	0	3.4	25	50	8.2

Table S11: Water usage for conventional and membrane processes

Process	Freshwater (L/s)	Brine (L/s)	Freshwater (L/year.perLCE)	Brine (L/year.perLCE)	Freshwater produced (L/year.perLCE)
Conventional	138	950	217598.4	1497960	0
NF	0	950	0	1497960	0
NF+HPRO	0	380	0	599184	898776
NF+HPRO+ZLD	0	190	0	299592	1198368
NF+HPRO+ZLD+BPED	0	190	0	299592	1198368

3. Produced water

Technical report used: E3 Metals Corp. NI 43-101 Technical Report Preliminary Economic Assessment, Clearwater Lithium Project Alberta, Canada [32]

Freshwater flow = 138 L/s

Brine flow = 1490 L/s

Total energy = 88.7 MW

Table S12: Energy breakdown for conventional process and membrane processes

Process	Brine Production	Brine injection	Pretreatment	DLE (IX)	Lithium production	Site costs	NF	HPRO	ZLD	BPED
Conventional	21	28.1	17.4	1.7	20.3	0.26	0	0	0	0
NF	21	28.1	17.4	0	20.3	0.26	5.3	0	0	0
NF+HPRO	21	0	17.4	0	20.3	0.26	5.3	39.3	0	0
NF+HPRO+ZLD	21	0	17.4	0	0	0.26	5.3	39.3	79.7	0
NF+HPRO+ZLD +BPED	21	0	17.4	0	0	0.26	5.3	39.3	79.7	8.2

Table S13: Water usage for conventional and membrane processes

Process	Freshwater (L/s)	Brine (L/s)	Freshwater (L/year.perLCE)	Brine (L/year.perLCE)	Water produced (L/year.perLCE)
Conventional	138	1490	217598.4	2349432	0
NF	0	1490	0	2349432	0
NF+HPRO	0	893	0	1408082	941349.6
NF+HPRO+ZLD	0	298	0	469886.4	1879545.6
NF+HPRO+ZLD+BPED	0	298	0	469886.4	1879545.6

Appendix D: Lithium selective membranes performance metrics

Table S14: Li/Mg selectivity and water permeability for nanofiltration membranes

No.	Mechanism	Selectivity (Li/Mg)	Water permeability (LMH/bar)	Ref.
1	Macrocycles	10.4	10.4	[33]
2	Macrocycles	11.2	10.4	[33]
3	Size based	78.56	8.2	[34]
4	Macrocycles	23.61	2.95	[35]
5	MOF/COF	64.7	2.4	[36]
6	Donnan exclusion	95.8	10	[37]
7	Donnan exclusion	95.9	10	[37]
8	Donnan exclusion	91.1	10	[37]
9	Donnan exclusion	49.4	10	[37]
10	Donnan exclusion	44.6	10	[37]
11	Size based	94.91	26.433	[38]
12	Size based	116.8	23.286	[38]
13	Size based	72.19	26.754	[38]
14	Donnan exclusion, Size based	69.2	5.42	[39]
15	Donnan exclusion, Size based	80.62	5.42	[39]
16	Donnan exclusion, Size based	46.79	5.42	[39]
17	Donnan exclusion, MOF/COF	102	17	[40]
18	Donnan exclusion	15	3.1	[41]
19	Donnan exclusion	21	3.1	[41]
20	Donnan exclusion	15	3.1	[41]
21	Donnan exclusion, Size based	131.37	0.76	[42]
22	Donnan exclusion	32.3	10.3	[43]
23	Donnan exclusion	33.6	9.6	[43]
24	MOF/COF	21.3	19.6	[44]
25	Size based	28	16.6	[45]
26	2D materials	3.81	6.35	[46]
27	Donnan exclusion, Size based	88	25	[47]
28	Donnan exclusion, Size based	65	25	[47]
29	Donnan exclusion, Size based	25	25	[47]
30	Donnan exclusion	167	8	[48]
31	2D materials, Donnan exclusion	37	12.33	[49]
32	2D materials, Donnan exclusion	20	12.33	[49]
33	2D materials, Donnan exclusion	49	12.33	[49]
34	Donnan exclusion	16.7	4.2	[50]
35	Donnan exclusion	11.6	9.6	[51]
36	Donnan exclusion	11.6	9.2	[51]
37	Donnan exclusion	13.9	9.1	[51]
38	Donnan exclusion	3.4	6.7	[52]
39	Donnan exclusion	41.2	3	[52]

40	Donnan exclusion	40.8	10.5	[52]
41	Donnan exclusion	15.4	19.2	[53]
42	Donnan exclusion	24	12.6	[54]
43	Size based	78.2	12.28	[55]
44	Size based	71.7	12.2	[55]
45	Donnan exclusion, Size based	25	4.5	[56]
46	Donnan exclusion, Size based	19	5	[56]
47	Donnan exclusion, Size based	15	5	[56]
48	Donnan exclusion	8.7	22	[57]
49	Donnan exclusion	117	9.3	[58]
50	Donnan exclusion	102	9.3	[58]
51	Donnan exclusion	105	9.3	[58]
52	Donnan exclusion	130	10.2	[59]
53	Donnan exclusion	13.5	12.5	[60]
54	Donnan exclusion	18.26	10.6	[61]
55	Donnan exclusion	54.8	14.2	[62]
56	Donnan exclusion	11.38	2.9	[63]
57	Donnan exclusion, Size based	8.7	14	[64]
58	Donnan exclusion, Size based	15.3	1.3	[65]
59	Donnan exclusion	6.9	1.81	[66]
60	Donnan exclusion	81.5	8	[67]
61	2D materials, Donnan exclusion	18	4	[68]
62	Donnan exclusion	11	4.3	[69]
63	Donnan exclusion, MOF/COF,	30.2	32	[70]
64	Donnan exclusion	15.15	2.5	[71]
65	Donnan exclusion	18	4.8	[72]
66	2D materials, Donnan exclusion	23.3	3.8	[73]
67	Donnan exclusion	12.9	6.1	[74]
68	Donnan exclusion	82.8	7.3	[75]
69	Donnan exclusion, Size based	58.7	17.4	[76]
70	Donnan exclusion	115	15.7	[77]
71	Donnan exclusion	15.4	1	[78]
72	Donnan exclusion, Size based	50.7	18.6	[79]
73	Donnan exclusion	5.2	16.2	[80]
74	Donnan exclusion	60	13	[81]
75	Donnan exclusion, Size based	75	18	[82]
76	Donnan exclusion, Size based	430	7	[83]
77	Donnan exclusion, Size based	100	14	[84]
78	Macrocycles	14	8	[85]
79	Donnan exclusion, Size based	50	10	[86]
80	Donnan exclusion	9.22	0.82	[87]
81	Donnan exclusion	10.1	16.3	[88]
82	2D materials, Donnan exclusion	48	10.1	[89]
83	Liquid membrane	9.8	10	[90]
84	Donnan exclusion	26	37	[91]
85	Macrocycles	21.9	18.5	[92]

86	Donnan exclusion	28	10	[93]
87	Donnan exclusion, Size based	12.5	8	[94]
88	Donnan exclusion	15.6	18.8	[95]
89	Size based	37.8	21.9	[96]
90	Donnan exclusion, MOF/COF,	78.6	5	[97]
91	Donnan exclusion, Size based	36.5	13.5	[98]
92	Donnan exclusion	36.4	1.25	[99]
93	Donnan exclusion	46.2	11	[100]
94	Donnan exclusion, Macrocycles	10.8	4.86	[101]
95	Donnan exclusion, MOF/COF	33	30.6	[102]
96	2D materials, Donnan exclusion	23.9	5.6	[103]
97	Donnan exclusion	14.42	11.98	[104]
98	Donnan exclusion, Size based	12.37	8.33	[105]
99	Donnan exclusion, Size based	23.4	22	[106]
100	Donnan exclusion	8	16.2	[107]
101	Donnan exclusion	58	12.23	[108]
102	Donnan exclusion	27.8	11.94	[109]
103	Donnan exclusion	58.66	11.46	[110]
104	Donnan exclusion	33.4	12	[111]
105	Donnan exclusion, Size based	7.15	10	[112]
106	Size based	5.8	3.4	[113]
107	Donnan exclusion	12.1	4.1	[114]
108	Liquid membrane	8	4.1	[115]
109	Donnan exclusion	0.32	9.5	[116]
110	2D materials, Donnan exclusion	16.3	11.1	[117]
111	Donnan exclusion, Size based	13.1	6.7	[118]
112	Donnan exclusion, Size based	20	5	[119]

Table S15: Lithium purity for Li/Mg separation for different nanofiltration membranes

No.	Mechanism	MLR	Water permeability (LMH/bar)	Lithium purity	Ref.
1	Macrocycles	20	10.4	0.34211	[33]
2	Macrocycles	50	10.4	0.18301	[33]
3	Size based	27.3	8.2	0.74211	[34]
4	Macrocycles	30	2.95	0.4404	[35]
5	MOF/COF, Size based	20	2.4	0.76387	[36]
6	Donnan exclusion	5	10	0.9504	[37]
7	Donnan exclusion	20	10	0.82744	[37]
8	Donnan exclusion	35	10	0.72244	[37]
9	Donnan exclusion	70	10	0.41374	[37]
10	Donnan exclusion	105	10	0.29813	[37]
11	Size based	10	26.433	0.90468	[38]

12	Size based	20	23.286	0.8538	[38]
13	Size based	20	26.754	0.78306	[38]
14	Donnan exclusion, Size based	40	5.42	0.6337	[39]
15	Donnan exclusion, Size based	60	5.42	0.57332	[39]
16	Donnan exclusion, Size based	120	5.42	0.28053	[39]
17	Donnan exclusion, MOF/COF	20	17	0.83607	[40]
18	Donnan exclusion	20	3.1	0.42857	[41]
19	Donnan exclusion	50	3.1	0.29577	[41]
20	Donnan exclusion	100	3.1	0.13043	[41]
21	Donnan exclusion	28	0.76	0.82431	[42]
22	Donnan exclusion	20	10.3	0.61759	[43]
23	Donnan exclusion	20	9.6	0.62687	[43]
24	MOF/COF, Size based	30	19.6	0.4152	[44]
25	Size based	50	16.6	0.35897	[45]
26	2D materials, Size based	20	6.35	0.16002	[46]
27	Donnan exclusion, Size based	31	25	0.7395	[47]
28	Donnan exclusion, Size based	48.2	25	0.5742	[47]
29	Donnan exclusion, Size based	80.5	25	0.23697	[47]
30	Donnan exclusion	20	8	0.89305	[48]
31	2D materials, Donnan exclusion	48	12.33	0.43529	[49]
32	2D materials, Donnan exclusion	84	12.33	0.19231	[49]
33	2D materials, Donnan exclusion	27	12.33	0.64474	[49]
34	Donnan exclusion	20	4.2	0.45504	[50]
35	Donnan exclusion	50	9.6	0.18831	[51]
36	Donnan exclusion	120	9.2	0.08815	[51]
37	Donnan exclusion	150	9.1	0.07178	[51]
38	Donnan exclusion	20	6.7	0.1453	[52]
39	Donnan exclusion	20	3	0.6732	[52]
40	Donnan exclusion	20	10.5	0.67105	[52]
41	Donnan exclusion, Size based	100	19.2	0.13345	[53]
42	Donnan exclusion	150	12.6	0.13793	[54]
43	Size based	30	12.28	0.72274	[55]
44	Size based	100	12.2	0.41759	[55]
45	Donnan exclusion, Size based	20	4.5	0.55556	[56]

46	Donnan exclusion, Size based	50	5	0.27536	[56]
47	Donnan exclusion, Size based	100	5	0.13043	[56]
48	Donnan exclusion	100	22	0.08004	[57]
49	Donnan exclusion	20	9.3	0.85401	[58]
50	Donnan exclusion	40	9.3	0.71831	[58]
51	Donnan exclusion	60	9.3	0.63636	[58]
52	Donnan exclusion	200	10.2	0.39394	[59]
53	Donnan exclusion	1	12.5	0.93103	[60]
54	Donnan exclusion	30	10.6	0.37837	[61]
55	2D materials, Donnan exclusion	23	3.8	0.50324	[73]
56	Donnan exclusion, Size based	15.3	17.4	0.79324	[76]

Table S16: Lithium flux and Li/Mg selectivity for electrically driven membranes

No.	Process	Mechanism	Li Flux (mol/m^2 hr)	S (Li/Mg)	Ref.
1	Diffusion	MOF/COF	0.68	13.2	[120]
2	Diffusion	MOF/COF	0.151	11.5	[121]
3	Diffusion	MOF/COF	0.238	5.92	[122]
4	Diffusion	2D materials	1.3	23.8	[123]
5	Diffusion	Liquid membrane, Macrocycles	0.128	19.1	[124]
6	Diffusion	2D materials, MOF/COF	1.73	31.9	[125]
7	Diffusion	Donnan exclusion	0.306	21.9	[126]
8	Diffusion	Adsorption	0.0032	2.24	[127]
9	Diffusion	Adsorption	0.0047	10.2	[128]
10	Diffusion	Adsorption	0.0016	184	[129]
11	Diffusion	2D materials	0.08	28	[130]
12	Diffusion	Donnan exclusion, MOF/COF	0.038	35.8	[131]
13	Diffusion	Liquid membrane	8E-4	176	[132]
14	Diffusion	Adsorption	3.23	3.1	[133]
15	Electrodialysis	2D materials	0.0451	54.1	[134]
16	Electrodialysis	MOF/COF, Macrocycles	0.002	6.9	[135]
17	Electrodialysis	2D materials, MOF/COF	0.009	5	[136]
18	Electrodialysis	2D materials, Donnan exclusion	0.007	4.78	[137]
19	Electrodialysis	2D materials	0.28	7.3	[138]
20	Electrodialysis	2D materials	0.043	6.2	[138]
21	Electrodialysis	Size based	0.124	5032	[139]
22	Electrodialysis	MOF/COF	27	159.4	[140]
23	Electrodialysis	Size based	0.0012	12.2	[141]
24	Electrodialysis	Donnan exclusion	0.032	17	[142]
25	Electrodialysis	2D materials, Size based	0.6	1.5	[143]
26	Electrodialysis	Adsorption, Donnan exclusion	0.026	9.1	[144]

27	Electrodialysis	Adsorption, Donnan exclusion	0.12	5	[144]
28	Electrodialysis	2D materials	1.02	31.8	[145]
29	Electrodialysis	Size based	0.06	84.11	[146]
30	Electrodialysis	MOF/COF, Size based	0.072	65	[147]
31	Electrodialysis	MOF/COF	6.75	1815	[148]
32	Electrodialysis	2D materials, Size based	0.4	4.27	[149]
33	Electrodialysis	2D materials, Size based	0.17	8.89	[149]
34	Electrodialysis	Donnan exclusion	0.756	29	[150]
35	Electrodialysis	Donnan exclusion	0.531	2.63	[151]
37	Electrodialysis	2D materials	0.092	10.2	[152]
38	Electrodialysis	2D materials	0.14	30	[152]
39	Electrodialysis	2D materials	2.77	12.8	[153]
40	Electrodialysis	Donnan exclusion	1.38	15.69	[154]
41	Electrodialysis	Macrocycles	0.081	24.35	[155]
42	Electrodialysis	2D materials	0.16	33	[156]
43	Electrodialysis	Size based	2.3E-4	25	[157]
44	Electrodialysis	Macrocycles	0.402	19.2	[158]
45	Electrodialysis	2D materials	0.09	22.2	[159]
46	Electrodialysis	Donnan exclusion	1.62	18	[160]
47	Electrodialysis	Donnan exclusion, MOF/COF	0.328	8.99	[161]
48	Electrodialysis	Donnan exclusion	0.31	14	[162]
49	Electrodialysis	Donnan exclusion	1.62	16	[163]
50	Electrodialysis	Donnan exclusion	0.18	14	[164]
51	Electrodialysis	Size based	0.077	100	[165]
52	Electrodialysis	Donnan exclusion	1.6	4.59	[166]
54	Electrodialysis	Donnan exclusion, MOF/COF	0.0045	450	[167]
55	Electrodialysis	Donnan exclusion, MOF/COF	0.005	390	[167]
57	Electrodialysis	Donnan exclusion	0.0102	1.7	[168]
58	Electrodialysis	Donnan exclusion	0.12	8	[169]
59	Electrodialysis	Donnan exclusion	0.19	21	[170]
60	Electrodialysis	Donnan exclusion	0.011	1.6	[171]
61	Electrodialysis	Adsorption	4.32	4.81	[172]
62	Electrodialysis	Size based	1.152	3.7	[173]
63	Electrodialysis	Donnan exclusion	2.08	11.3	[174]
64	Electrodialysis	Donnan exclusion, MOF/COF	1.94	11.38	[175]
65	Electrodialysis	Donnan exclusion	0.882	12.7	[176]
66	Electrodialysis	Donnan exclusion	0.16	5.16	[177]
67	Electrodialysis	Donnan exclusion	0.17	16.5	[178]

Table S17: Adsorption capacity and Li/Mg selectivity for adsorptive membranes

No.	Adsorbent	Li adsorption capacity (mg/g)	Li/Mg selectivity	Ref.
1	Mn based	27.8	4.76	[179]

2	Mn based	18.3	51.63	[180]
3	Silica based	231.77	3.55	[181]
4	Mn based	23.26	23.5	[182]
5	Sulfonate group	20.54	48.3	[183]
6	Mn based	31.55	27.25	[184]
7	Mn based	29.2	318.84	[184]
8	Ti based	20.08	2.6	[185]
9	14C4	40.1	8.47	[186]
10	12C4	132	6.8	[187]
11	12C4	297	1.33	[188]
12	12C4	16.4	4.13	[189]
13	14C4	34.05	23.5	[190]
14	12C4	24.25	8.02	[191]
15	12C4	25.58	4.75	[192]
16	12C4	27.1	4.42	[193]

Table S118: Lithium flux and Li/Na selectivity for electrically driven membranes

No.	Process	Mechanism	Li Flux (mol/m^2 hr)	S (Li/Na)	Ref.
1	Diffusion	MOF/COF	0.151	1.93	[121]
2	Diffusion	2D materials, Size based	0.1741	29.5	[194]
3	Diffusion	2D materials	1.3	14.9	[123]
4	Diffusion	2D materials, MOF/COF	1.73	15.8	[125]
5	Diffusion	Size based	0.022	1389	[195]
6	Diffusion	2D materials	0.08	15.5	[130]
7	Diffusion	Adsorption	2.153	931	[196]
8	Diffusion	Adsorption	4.763	5543	[196]
9	Diffusion	Size based	0.6552	278	[197]
10	Diffusion	Liquid membrane	0.01	54.25	[198]
11	Diffusion	Adsorption	0.036	7	[199]
23	Electrodialysis	2D materials	0.02	2.5	[200]
24	Electrodialysis	Size based	0.124	242	[139]
25	Electrodialysis	Adsorption	129	2.56	[201]
26	Electrodialysis	2D materials	1.02	1.52	[145]
27	Electrodialysis	Size based	0.06	22.04	[146]
28	Electrodialysis	MOF/COF	6.75	35	[148]
30	Electrodialysis	2D materials	2.77	5.9	[153]
31	Electrodialysis	2D materials	66	1.85	[202]
33	Electrodialysis	Adsorption	8.94	32.2	[203]
34	Electrodialysis	Adsorption	4.32	2.2	[172]

Table S19: Adsorption capacity and Li/Na selectivity for adsorptive membranes

No.	Adsorbent	Li adsorption capacity (mg/g)	Li/Na selectivity	Ref.
1	Mn based	18.3	55.33	[180]
2	Silica based	231.77	5.03	[181]
3	Mn based	23.26	186.4	[182]
4	Mn based	51.8	10.1	[204]
5	Sulfonate group	20.54	5.09	[183]
6	Ti based	20.08	1.78	[185]
7	14C4	40.17	52.3	[205]
8	12C4	68	1.33	[206]
9	14C4	40.1	14	[186]
10	12C4	132	24	[187]
11	12C4	297	3.46	[188]
12	12C4	16.4	4.2	[189]
13	14C4	34.05	45.6	[190]
14	12C4	24.25	7.83	[191]
15	12C4	799	39	[207]

References:

[1] Lithium global supply projection 2030, Statista (n.d.). https://www.statista.com/statistics/452026/projected-total-supply-for-lithium-globally/ (accessed September 18, 2024).

[2] Lithium global demand forecast 2025, Statista (n.d.). https://www.statista.com/statistics/452025/projected-total-demand-for-lithium-globally/ (accessed September 18, 2024).

[3] W.T. Stringfellow, P.F. Dobson, Technology for the recovery of lithium from geothermal brines, Energies 14 (2021). https://doi.org/10.3390/en14206805.

[4] Vulcan Lithium Project, NS Energy (n.d.). https://www.nsenergybusiness.com/projects/vulcan-lithium-project/ (accessed September 18, 2024).

[5] L. Kölbel, T. Kölbel, L. Herrmann, E. Kaymakci, I. Ghergut, A. Poirel, J. Schneider, Lithium extraction from geothermal brines in the Upper Rhine Graben: A case study of potential and current state of the art, Hydrometallurgy 221 (2023) 106131. https://doi.org/10.1016/j.hydromet.2023.106131.

[6] A. Kumar, H. Fukuda, T.A. Hatton, J.H.V. Lienhard, Lithium Recovery from Oil and Gas Produced Water: A Need for a Growing Energy Industry, ACS Energy Lett. 4 (2019) 1471–1474. https://doi.org/10.1021/acsenergylett.9b00779.

[7] D. SM, Indigo Exploration - Home, (n.d.). https://indigoexploration.com/ (accessed September 18, 2024).

[8] EV battery recycling capacity by country 2023, Statista (n.d.). https://www.statista.com/statistics/1333941/worldwide-ev-battery-recycling-capacity-by-country/ (accessed September 18, 2024).

[9] S. Yang, F. Zhang, H. Ding, P. He, H. Zhou, Lithium Metal Extraction from Seawater, Joule 2 (2018) 1648–1651. https://doi.org/10.1016/j.joule.2018.07.006.

[10] S. Khakmardan, M. Rolinck, F. Cerdas, C. Herrmann, D. Giurco, R. Crawford, W. Li, Comparative Life Cycle Assessment of Lithium Mining, Extraction, and Refining Technologies: a Global Perspective, Procedia CIRP 116 (2023) 606–611. https://doi.org/10.1016/j.procir.2023.02.102.

[11] M.L. Vera, W.R. Torres, C.I. Galli, A. Chagnes, V. Flexer, Environmental impact of direct lithium extraction from brines, Nat Rev Earth Environ 4 (2023) 149–165. https://doi.org/10.1038/s43017-022-00387-5.

[12] A. Kronbauer, Vulcan Zero Carbon Lithium™ Project Phase One DFS results and Resources-Reserves update, VULCAN ENERGY RESOURCES (2023). https://v-er.eu/blog/vulcan-zero-carbon-lithium-project-phase-one-dfs-results-and-resources-reserves-update/ (accessed September 18, 2024).

[13] E3 Lithium - Technical Reports, (n.d.). https://www.e3lithium.ca/our-assets/technical-reports/ (accessed September 18, 2024).

[14] E. Yoo, U. Lee, J.C. Kelly, M. Wang, Life-cycle analysis of battery metal recycling with lithium recovery from a spent lithium-ion battery, Resources, Conservation and Recycling 196 (2023) 107040. https://doi.org/10.1016/j.resconrec.2023.107040.

[15] J. Qing, X. Wu, L. Zeng, W. Guan, Z. Cao, Q. Li, M. Wang, G. Zhang, S. Wu, Novel approach to recycling of valuable metals from spent lithium-ion batteries using hydrometallurgy, focused on preferential extraction of lithium, Journal of Cleaner Production 431 (2023) 139645. https://doi.org/10.1016/j.jclepro.2023.139645.

[16] Form Center • Water and Wastewater Facility Tours, (n.d.). https://www.cfpua.org/FormCenter/Water-Treatment-16/Water-Treatment-Facility-Tours-114 (accessed May 11, 2024).

[17] Sweeney Treatment Enhancements Project | Cape Fear Public Utility Authority Official Site, (n.d.). https://www.cfpua.org/775/Sweeney-Treatment-Enhancements-Project (accessed May 11, 2024).
[18] M.-H. Cheng, K.A. Rosentrater, Economic feasibility analysis of soybean oil production by hexane extraction, Industrial Crops and Products 108 (2017) 775–785. https://doi.org/10.1016/j.indcrop.2017.07.036.
[19] Oil and Oilseed Processing II - Oklahoma State University, (2016). https://extension.okstate.edu/fact-sheets/oil-and-oilseed-processing-ii.html (accessed May 11, 2024).
[20] Constructing the Nation's Largest Ion Exchange PFAS Water Treatment Plant, Association of California Water Agencies (n.d.). https://www.acwa.com/events/constructing-the-nations-largest-ion-exchange-pfas-water-treatment-plant/ (accessed May 11, 2024).
[21] Today the World's Largest Reverse Osmosis Desalination Facility Ranked by the Guinness World Records™, Rabigh 3 IWP was inaugurated in Kingdom of Saudi Arabia, Idadesal (2022). https://idadesal.org/today-the-worlds-largest-reverse-osmosis-desalination-facility-ranked-by-the-guinness-world-records-rabigh-3-iwp-was-inaugurated-in-kingdom-of-saudi-arabia/ (accessed May 11, 2024).
[22] DEFINITIVE FEASIBILITY STUDY OF MSB BLANCO LITHIUM CARBONATE PROJECT, (n.d.). https://lithiumpowerinternational.com/wp-content/uploads/2019/01/Definitive-Feasibility-Study-of-MSB-Blanco-Lithium-Carbonate-Project.pdf (accessed October 12, 2024).
[23] Gasoline and Diesel Fuel Update, (n.d.). https://www.eia.gov/petroleum/gasdiesel/index.php (accessed September 20, 2024).
[24] Energy conversion calculators - U.S. Energy Information Administration (EIA), (n.d.). https://www.eia.gov/energyexplained/units-and-calculators/energy-conversion-calculators.php (accessed September 20, 2024).
[25] M. Figueira, J. López, M. Reig, J.L. Cortina, C. Valderrama, Techno-economic analysis of seawater reverse osmosis brines treatment using nanofiltration modelling tools, Desalination 568 (2023) 117013. https://doi.org/10.1016/j.desal.2023.117013.
[26] A. Anvari, J. Wu, A. Edalat, N. Voutchkov, A. Al-Ahmoudi, S. Bhattacharjee, E.M.V. Hoek, What will it take to get to 250,000 ppm brine concentration via ultra-high pressure reverse osmosis? And is it worth it?, Desalination 580 (2024) 117565. https://doi.org/10.1016/j.desal.2024.117565.
[27] A. Panagopoulos, Techno-economic assessment and feasibility study of a zero liquid discharge (ZLD) desalination hybrid system in the Eastern Mediterranean, Chemical Engineering and Processing - Process Intensification 178 (2022) 109029. https://doi.org/10.1016/j.cep.2022.109029.
[28] A. González, M. Grágeda, S. Ushak, Modeling and Validation of a LiOH Production Process by Bipolar Membrane Electrodialysis from Concentrated LiCl, Membranes 13 (2023) 187. https://doi.org/10.3390/membranes13020187.
[29] Vulcan Zero Carbon Lithium™ Project Phase One DFS results, (n.d.). https://www.investi.com.au/api/announcements/vul/e617fca6-6d4.pdf (accessed October 12, 2024).
[30] Positive Zero Carbon Lithium Project Bridging Study Results - Vulcan Energy Resources Limited (ASX:VUL), (2023). https://www.listcorp.com/asx/vul/vulcan-energy-resources-limited/news/positive-zero-carbon-lithium-project-bridging-study-results-2957205.html (accessed October 12, 2024).
[31] BRIDGING ENGINEERING STUDY RESULTS, PHASE ONE FINANCING LAUNCH EDITION, (n.d.). https://v-er.eu/app/uploads/2023/11/November-Launch-Presentation.pdf (accessed October 12, 2024).

[32] E3 Metals Corp. NI 43-101 Technical Report Preliminary Economic Assessment, Clearwater Lithium Project Alberta, (n.d.). https://minedocs.com/23/Clearwater-PEA-Amended-09172021.pdf (accessed October 12, 2024).
[33] Z. Zha, T. Li, I. Hussein, Y. Wang, S. Zhao, Aza-crown ether-coupled polyamide nanofiltration membrane for efficient Li+/Mg2+ separation, Journal of Membrane Science 695 (2024) 122484. https://doi.org/10.1016/j.memsci.2024.122484.
[34] S. Zhao, W. Cui, Q. Shen, Z. Yao, C. Fang, L. Zhang, L. Zhu, Porous organic polymer interlayers modulated nanofiltration membranes for ultra-permselective Li+ /Mg2+ separation, Journal of Membrane Science 690 (2024) 122207. https://doi.org/10.1016/j.memsci.2023.122207.
[35] N. Li, T. Zhang, W. Xue, Y. Zhao, B. Zhu, X. Pei, Z. Xu, Tuning composite nanofiltration membranes with γ-cyclodextrin for improved Mg2+/Li+ selectivity, Separation and Purification Technology 330 (2024) 125206. https://doi.org/10.1016/j.seppur.2023.125206.
[36] Y. Zhang, Z. Hao, I. Hussein, Z. Wang, S. Zhao, Tunable Ionic Sieving Membrane via Reactive Layer-By-Layer Assembly of Porous Organic Cages, Advanced Functional Materials 34 (2024) 2315750. https://doi.org/10.1002/adfm.202315750.
[37] G. Zhao, J. Sun, G. Tang, G. Pan, H. Yu, Y. Li, Y. Zhang, Y. Liu, Highly selective Mg2+/Li+ separation membranes prepared by surface grafting of a novel quaternary ammonium bromide, Separation and Purification Technology 335 (2024) 126184. https://doi.org/10.1016/j.seppur.2023.126184.
[38] B. Yuan, Y. Zhang, P. Qi, D. Yang, P. Hu, S. Zhao, K. Zhang, X. Zhang, M. You, J. Cui, J. Jiang, X. Lou, Q.J. Niu, Self-assembled dendrimer polyamide nanofilms with enhanced effective pore area for ion separation, Nat Commun 15 (2024) 471. https://doi.org/10.1038/s41467-023-44530-2.
[39] Y. Zhang, Y. Fan, G. Zhou, Y. Cao, J. Wang, X. Jiang, N. Zhang, S. Yin, Based on high cross-linked structure design to fabricate PEI-based nanofiltration membranes for Mg2+/Li+ separation, Journal of Membrane Science 693 (2024) 122351. https://doi.org/10.1016/j.memsci.2023.122351.
[40] R. Jia, L.-K. Wu, Z.-L. Xu, M. Hedar, L.-H. Luo, Y.-Z. Wu, H.-X. Li, Y.-H. Tong, S.-J. Xu, Efficient separation of Li+/Mg2+ via positively charged TFN membrane based on the PEI interlayer, Chemical Engineering Science 284 (2024) 119523. https://doi.org/10.1016/j.ces.2023.119523.
[41] Y. Zhang, W.-S. Zou, W. Kong, X. Chen, W. Li, X. Huang, Y. Wang, Dual integration of amine-functionalized carbon dots endowed nanofiltration membranes with highly efficient biofouling/ acid/chlorine resistance for effective Mg2+/Li+ separation, Journal of Membrane Science 696 (2024) 122542. https://doi.org/10.1016/j.memsci.2024.122542.
[42] X. Cheng, Q. Pan, H. Tan, K. Chen, W. Liu, Y. Shi, S. Du, B. Zhu, The construction of an efficient magnesium–lithium separation thin film composite membrane with dual aqueous-phase monomers (PIP and MPD), RSC Adv. 13 (2023) 22113–22121. https://doi.org/10.1039/D3RA04258H.
[43] L. Li, G. Zhu, Y. Tong, K. Ding, Z. Wang, C. Meng, C. Gao, Polyethyleneimine modified polyamide composite nanofiltration membrane for separation of lithium and magnesium, Journal of Water Process Engineering 54 (2023) 103894. https://doi.org/10.1016/j.jwpe.2023.103894.
[44] Z. Si, Z. Zhang, C. Yin, T. Ju, M. Wei, J. Huang, Y. Wang, Engineering transport highways in microporous membranes for lithium extraction: The double role of covalent organic frameworks, Journal of Membrane Science 680 (2023) 121759. https://doi.org/10.1016/j.memsci.2023.121759.
[45] J. Li, H. Peng, K. Liu, Q. Zhao, Polyester Nanofiltration Membranes for Efficient Cations Separation, Advanced Materials 36 (2024) 2309406. https://doi.org/10.1002/adma.202309406.

[46] Z. Feng, C. Liu, B. Tang, X. Yang, W. Jiang, P. Wang, X. Tang, H. Wang, X. Zeng, G. Zeng, Construction of a Two-Dimensional GO/Ti3C2TX Composite Membrane and Investigation of Mg2+/Li+ Separation Performance, Nanomaterials 13 (2023) 2777. https://doi.org/10.3390/nano13202777.
[47] K. Chen, F. Li, T. Wei, H. Zhou, T. Zhang, S. Zhao, T. Xie, H. Sun, P. Li, Q.J. Niu, An interlayer-based positive charge compensation strategy for the preparation of highly selective Mg2+/Li+ separation nanofiltration membranes, Journal of Membrane Science 684 (2023) 121882. https://doi.org/10.1016/j.memsci.2023.121882.
[48] G. Zhao, Y. Zhang, Y. Li, G. Pan, Y. Liu, Positively charged nanofiltration membranes for efficient Mg2+/Li+ separation from high Mg2+/Li+ ratio brine, Advanced Membranes 3 (2023) 100065. https://doi.org/10.1016/j.advmem.2023.100065.
[49] L. Ma, Q. Bi, W. Zhou, X. Liu, F. Qi, H. Zhang, Y. Gao, S. Xu, Nanofiltration membrane with a zwitterion-g-C3N4 composite interlayer for Mg2+/Li+ separation, Journal of Water Process Engineering 53 (2023) 103751. https://doi.org/10.1016/j.jwpe.2023.103751.
[50] Z. Zhao, N. Di, Z. Zha, J. Wang, Z. Wang, S. Zhao, Positively Charged Polyamine Nanofiltration Membrane for Precise Ion–Ion Separation, ACS Appl. Mater. Interfaces 15 (2023) 48695–48704. https://doi.org/10.1021/acsami.3c11076.
[51] S. Zhang, Y. Jiang, X. Yue, R. Zhang, R. Li, T. Gu, T. Wu, J. Zhao, S. Zhang, Z. Jiang, Bifunctional polyhedral oligomeric silsesquioxane engineered polyamide membrane for efficient Li+/Mg2+ separation, Separation and Purification Technology 327 (2023) 124875. https://doi.org/10.1016/j.seppur.2023.124875.
[52] Q. Wang, Y. Wang, Y. Huang, H. Wang, Y. Gao, M. Zhao, L. Tu, L. Xue, C. Gao, Polyethyleneimine (PEI) based positively charged thin film composite polyamide (TFC-PA) nanofiltration (NF) membranes for effective Mg2+/Li+ separation, Desalination 565 (2023) 116814. https://doi.org/10.1016/j.desal.2023.116814.
[53] H. Peng, Y. Su, X. Liu, J. Li, Q. Zhao, Designing Gemini-Electrolytes for Scalable Mg2+/Li+ Separation Membranes and Modules, Advanced Functional Materials 33 (2023) 2305815. https://doi.org/10.1002/adfm.202305815.
[54] E. Wang, S. Liu, L. Liu, L. Han, B. Su, Positively charged thin-film composite hollow fiber nanofiltration membrane via interfacial polymerization and branch polyethyleneimine modification for Mg2+/Li+ separation, Journal of Membrane Science Letters 3 (2023) 100061. https://doi.org/10.1016/j.memlet.2023.100061.
[55] G. Zhao, H. Gao, Z. Qu, H. Fan, H. Meng, Anhydrous interfacial polymerization of sub-1 Å sieving polyamide membrane, Nat Commun 14 (2023) 7624. https://doi.org/10.1038/s41467-023-43291-2.
[56] S. Hu, T. Liu, Z. Zhou, J. Xiao, J. Yang, Y. Hu, A novel interfacial polymerization assisted phase inversion process for the facile fabrication of ion-selective nanofiltration membranes, Chemical Engineering Journal 477 (2023) 147212. https://doi.org/10.1016/j.cej.2023.147212.
[57] H. Peng, K. Yu, X. Liu, J. Li, X. Hu, Q. Zhao, Quaternization-spiro design of chlorine-resistant and high-permeance lithium separation membranes, Nat Commun 14 (2023) 5483. https://doi.org/10.1038/s41467-023-41169-x.
[58] P. Xu, K. Guan, Y.-H. Chiao, Z. Mai, Z. Li, M. Hu, P. Zhang, R.R. Gonzales, H. Matsuyama, Fine-tuning polyamide nanofiltration membrane for ultrahigh separation selectivity of Mg2+ and Li+, Journal of Membrane Science 688 (2023) 122133. https://doi.org/10.1016/j.memsci.2023.122133.
[59] Q. Li, Y. Liu, Y. Jia, Y. Ji, F. Yan, J. Li, Y. Mohammad, B. He, High performance Li+/Mg2+ separation membrane by grafted short chain amino-rich monomers, Journal of Membrane Science 677 (2023) 121634. https://doi.org/10.1016/j.memsci.2023.121634.

[60] Q.-Y. Zhu, Z.-Y. Xu, J.-H. Fu, J. Huang, Z.-L. Xu, M. Tong, E.-C. Li, Y.-J. Tang, Can the mix-charged NF membrane directly obtained by the interfacial polymerization of PIP and TMC?, Desalination 558 (2023) 116623. https://doi.org/10.1016/j.desal.2023.116623.
[61] C. Guo, Y. Qian, P. Liu, Q. Zhang, X. Zeng, Z. Xu, S. Zhang, N. Li, X. Qian, F. Yu, One-Step Construction of the Positively/Negatively Charged Ultrathin Janus Nanofiltration Membrane for the Separation of Li+ and Mg2+, ACS Applied Materials and Interfaces 15 (2023) 4814–4825. https://doi.org/10.1021/acsami.2c19956.
[62] C. Jiang, Z. Liu, L. Jiang, Z. Fei, Y. Hou, Rapid transport of water and monovalent ions through ultrathin polyamide nanofilms for highly efficient mono/bivalent ions separation, Applied Surface Science 608 (2023). https://doi.org/10.1016/j.apsusc.2022.155025.
[63] H. Li, Y. Li, M. Li, Y. Jin, G. Kang, Y. Cao, Improving Mg2+/Li+ separation performance of polyamide nanofiltration membrane by swelling-embedding-shrinking strategy, Journal of Membrane Science 669 (2023) 121321. https://doi.org/10.1016/j.memsci.2022.121321.
[64] T. Li, X. Zhang, Y. Zhang, J. Wang, Z. Wang, S. Zhao, Nanofiltration membrane comprising structural regulator Cyclen for efficient Li+/Mg2+ separation, Desalination 556 (2023). https://doi.org/10.1016/j.desal.2023.116575.
[65] Y. Li, S. Wang, H. Li, D. Liu, Y. Jin, G. Kang, Y. Cao, Polyamide nanofiltration membranes with rigid–flexible microstructures for high-efficiency Mg2+/Li+ separation, Separation and Purification Technology 306 (2023). https://doi.org/10.1016/j.seppur.2022.122552.
[66] X. Liu, Y. Feng, Y. Ni, H. Peng, S. Li, Q. Zhao, High-permeance Mg2+/Li+ separation nanofiltration membranes intensified by quadruple imidazolium salts, Journal of Membrane Science 667 (2023). https://doi.org/10.1016/j.memsci.2022.121178.
[67] Y. Liu, Q. Li, S. Wang, M. Liang, Y. Ji, Z. Cui, M. Younas, J. Li, B. He, A nanofiltration membrane with positively and negatively charged groups by grafted p-aminosalicylic acid-Fe(III) chelation for Li+/Mg2+ efficient separation, Separation and Purification Technology 308 (2023). https://doi.org/10.1016/j.seppur.2022.122968.
[68] H. Ni, N. Wang, Y. Yang, M. Shen, Q.-F. An, Positively-charged nanofiltration membrane constructed by polyethyleneimine/layered double hydroxide for Mg2+/Li+ separation, Desalination 548 (2023). https://doi.org/10.1016/j.desal.2022.116256.
[69] H. Peng, Y. Hu, S. Li, J. Rao, Q. Zhao, Sulfonium-polyamide membranes for high flux Mg2+/Li+ separation, Journal of Membrane Science 674 (2023). https://doi.org/10.1016/j.memsci.2023.121515.
[70] L. Ren, J. Chen, J. Han, J. Liang, H. Wu, Anti-scaling covalent organic framework membranes with custom-tailored nanochannels for efficient lithium extraction, Chemical Engineering Journal 462 (2023). https://doi.org/10.1016/j.cej.2023.142112.
[71] O. Setiawan, Y.-H. Huang, Z.G. Abdi, W.-S. Hung, T.-S. Chung, pH-tunable and pH-responsive polybenzimidazole (PBI) nanofiltration membranes for Li+/Mg2+ separation, Journal of Membrane Science 668 (2023). https://doi.org/10.1016/j.memsci.2022.121269.
[72] Q. Wang, Y. Dong, J. Ma, H. Wang, X. Xue, C. Bai, M. Lin, L. Luo, C. Gao, L. Xue, Polyamide/polyethylene thin film composite (PA/PE-TFC) NF membranes prepared from reverse-phase interface polymerization (RIP) for improved Mg(II)/Li(I) separation, Desalination 553 (2023). https://doi.org/10.1016/j.desal.2023.116463.
[73] R. Wang, J. Wu, J. Zheng, B. Chen, X. Zhu, Janus membrane with tailored upper and lower surface charges for ion penetration manipulation in high-performance nanofiltration, Journal of Membrane Science 667 (2023). https://doi.org/10.1016/j.memsci.2022.121191.
[74] H. Wu, H. Zhao, Y. Lin, X. Liu, L. Wang, H. Yao, Y. Tang, L. Yu, H. Wang, X. Wang, Positively-charged PEI/TMC nanofiltration membrane prepared by adding a diamino-silane coupling agent for Li+/Mg2+ separation, Journal of Membrane Science 672 (2023). https://doi.org/10.1016/j.memsci.2023.121468.
[75] P. Xu, R.R. Gonzales, J. Hong, K. Guan, Y.-H. Chiao, Z. Mai, Z. Li, S. Rajabzadeh, H. Matsuyama, Fabrication of highly positively charged nanofiltration membranes by novel

interfacial polymerization: Accelerating Mg2+ removal and Li+ enrichment, Journal of Membrane Science 668 (2023). https://doi.org/10.1016/j.memsci.2022.121251.
[76] B. Yuan, N. Wang, S. Zhao, P. Hu, J. Jiang, J. Cui, X. Zhang, M. You, X. Lou, T. Plisko, A.V. Bildyukevich, T.A. Hliavitskaya, Q. Jason Niu, Asymmetric polyamide nanofilm with coordinated charge and nanopore, tuned by azlactone-based monomer to facilitate ion separation, Separation and Purification Technology 304 (2023). https://doi.org/10.1016/j.seppur.2022.122361.
[77] T. Zhang, Y. Chen, Q. Yu, H. Sun, K. Chen, H. Ye, S. Tang, H. Zhang, P. Li, Q. Jason Niu, Advanced Mg2+/Li+ separation nanofiltration membranes by introducing hydroxypropyltrimethyl ammonium chloride chitosan as a co-monomer, Applied Surface Science 616 (2023). https://doi.org/10.1016/j.apsusc.2023.156434.
[78] M. Awais Ashraf, M. Usman, I. Hussain, F. Ahmad, S. Guo, L. Zhang, Lithium extraction from high magnesium salt lake brine with an integrated membrane technology, Separation and Purification Technology 302 (2022). https://doi.org/10.1016/j.seppur.2022.122163.
[79] K. Chen, S. Zhao, H. Lan, T. Xie, H. Wang, Y. Chen, P. Li, H. Sun, Q.J. Niu, C. Yang, Dual-electric layer nanofiltration membranes based on polyphenol/PEI interlayer for highly efficient Mg2+/Li+ separation, Journal of Membrane Science 660 (2022). https://doi.org/10.1016/j.memsci.2022.120860.
[80] Y. Feng, H. Peng, Q. Zhao, Fabrication of high performance Mg2+/Li+ nanofiltration membranes by surface grafting of quaternized bipyridine, Separation and Purification Technology 280 (2022). https://doi.org/10.1016/j.seppur.2021.119848.
[81] T. Gu, R. Zhang, S. Zhang, B. Shi, J. Zhao, Z. Wang, M. Long, G. Wang, T. Qiu, Z. Jiang, Quaternary ammonium engineered polyamide membrane with high positive charge density for efficient Li+/Mg2+separation, Journal of Membrane Science 659 (2022). https://doi.org/10.1016/j.memsci.2022.120802.
[82] R. He, C. Dong, S. Xu, C. Liu, S. Zhao, T. He, Unprecedented Mg2+/Li+ separation using layer-by-layer based nanofiltration hollow fiber membranes, Desalination 525 (2022). https://doi.org/10.1016/j.desal.2021.115492.
[83] R. He, S. Xu, R. Wang, B. Bai, S. Lin, T. He, Polyelectrolyte-based nanofiltration membranes with exceptional performance in Mg2+/Li+ separation in a wide range of solution conditions, Journal of Membrane Science 663 (2022). https://doi.org/10.1016/j.memsci.2022.121027.
[84] P. Hu, B. Yuan, Q.J. Niu, K. Chen, Z. Xu, B. Tian, X. Zhang, Modification of polyamide nanofiltration membrane with ultra-high multivalent cations rejections and mono-/divalent cation selectivity, Desalination 527 (2022). https://doi.org/10.1016/j.desal.2022.115553.
[85] H. Li, Y. Wang, T. Li, X.-K. Ren, J. Wang, Z. Wang, S. Zhao, Nanofiltration membrane with crown ether as exclusive Li+ transport channels achieving efficient extraction of lithium from salt lake brine, Chemical Engineering Journal 438 (2022) 135658. https://doi.org/10.1016/j.cej.2022.135658.
[86] Q. Li, Y. Liu, Y. Liu, Y. Ji, Z. Cui, F. Yan, J. Li, M. Younas, B. He, Mg2+/Li+ separation by electric field assisted nanofiltration：the impacts of membrane pore structure, electric property and other process parameters, Journal of Membrane Science 662 (2022). https://doi.org/10.1016/j.memsci.2022.120982.
[87] Y. Li, S. Wang, W. Wu, H. Yu, R. Che, G. Kang, Y. Cao, Fabrication of positively charged nanofiltration membrane with uniform charge distribution by reversed interfacial polymerization for Mg2+/Li+ separation, Journal of Membrane Science 659 (2022). https://doi.org/10.1016/j.memsci.2022.120809.
[88] H. Luo, H. Peng, Q. Zhao, High flux Mg2+/Li+ nanofiltration membranes prepared by surface modification of polyethylenimine thin film composite membranes, Applied Surface Science 579 (2022). https://doi.org/10.1016/j.apsusc.2021.152161.

[89] L. Ma, Q. Bi, Y. Tang, C. Zhang, F. Qi, H. Zhang, Y. Gao, S. Xu, Fabrication of High-Performance Nanofiltration Membrane Using Polydopamine and Carbon Nitride as the Interlayer, Separations 9 (2022). https://doi.org/10.3390/separations9070180.
[90] Y. Ni, H. Peng, Q. Zhao, Ultrathin Poly(Ionic Liquid) Nanomembranes for High Performance Mg2+/Li+ Separation, Advanced Materials Interfaces 9 (2022). https://doi.org/10.1002/admi.202201797.
[91] F. Soyekwo, H. Wen, D. Liao, C. Liu, Nanofiltration Membranes Modified with a Clustered Multiquaternary Ammonium-Based Ionic Liquid for Improved Magnesium/Lithium Separation, ACS Applied Materials and Interfaces 14 (2022) 32420–32432. https://doi.org/10.1021/acsami.2c03650.
[92] M. Wang, M. Li, Z. Fei, J. Li, Z. Ren, Y. Hou, Synergistic regulation of macrocyclic polyamine-based polyamide nanofiltration membranes by the interlayer and surfactant for divalent ions rejection and mono-/di-ions sieving, Desalination 544 (2022). https://doi.org/10.1016/j.desal.2022.116131.
[93] M.-B. Wu, H. Ye, Z.-Y. Zhu, G.-T. Chen, L.-L. Ma, S.-C. Liu, L. Liu, J. Yao, Z.-K. Xu, Positively-charged nanofiltration membranes constructed via gas/liquid interfacial polymerization for Mg2+/Li+ separation, Journal of Membrane Science 644 (2022) 119942. https://doi.org/10.1016/j.memsci.2021.119942.
[94] H.-Y. Xie, R.-H. Tang, G.-E. Chen, Z.-L. Xu, H.-F. Mao, Highly heat-resistant NF membrane modified by quinoxaline diamines for Li+ extraction from the brine, Journal of Industrial and Engineering Chemistry 112 (2022) 323–334. https://doi.org/10.1016/j.jiec.2022.05.028.
[95] Y. Xu, H. Peng, H. Luo, Q. Zhang, Z. Liu, Q. Zhao, High performance Mg2+/Li+ separation membranes modified by a bis-quaternary ammonium salt, Desalination 526 (2022). https://doi.org/10.1016/j.desal.2021.115519.
[96] Y. Yang, Y. Li, K. Goh, C.H. Tan, R. Wang, Dopamine-intercalated polyelectrolyte multilayered nanofiltration membranes: Toward high permselectivity and ion-ion selectivity, Journal of Membrane Science 648 (2022). https://doi.org/10.1016/j.memsci.2022.120337.
[97] B. Yuan, N. Wang, S. Zhao, P. Hu, J. Jiang, J. Cui, X. Zhang, M. You, X. Lou, Q.J. Niu, Polyamide nanofiltration membrane fine-tuned via mixed matrix ultrafiltration support to maximize the sieving selectivity of Li+/Mg2+ and Cl–/SO42–, Desalination 538 (2022). https://doi.org/10.1016/j.desal.2022.115929.
[98] B. Yuan, S. Zhao, S. Xu, N. Wang, P. Hu, K. Chen, J. Jiang, J. Cui, X. Zhang, M. You, Q.J. Niu, Aliphatic polyamide nanofilm with ordered nanostripe, synergistic pore size and charge density for the enhancement of cation sieving, Journal of Membrane Science 660 (2022). https://doi.org/10.1016/j.memsci.2022.120839.
[99] S. Zhang, R. Zhang, R. Li, Z. Zhang, Y. Li, H. Deng, J. Zhao, T. Gu, M. Long, X. Wang, S. Zhang, Z. Jiang, Guanidyl-incorporated nanofiltration membranes toward superior Li+/Mg2+ selectivity under weakly alkaline environment, Journal of Membrane Science 663 (2022). https://doi.org/10.1016/j.memsci.2022.121063.
[100] X. Zhang, F. Li, M. Liu, C. Zhu, X. Zhao, Positively charged modification of commercial nanofiltration membrane to enhance the separation of mono−/divalent cation, Journal of Applied Polymer Science 139 (2022). https://doi.org/10.1002/app.53204.
[101] Y. Zhao, N. Li, J. Shi, Y. Xia, B. Zhu, R. Shao, C. Min, Z. Xu, H. Deng, Extra-thin composite nanofiltration membranes tuned by γ-cyclodextrins containing amphipathic cavities for efficient separation of magnesium/lithium ions, Separation and Purification Technology 286 (2022). https://doi.org/10.1016/j.seppur.2021.120419.
[102] F. Aghili, A.A. Ghoreyshi, B. Van der Bruggen, A. Rahimpour, A highly permeable UiO-66-NH2/polyethyleneimine thin-film nanocomposite membrane for recovery of valuable metal ions from brackish water, Process Safety and Environmental Protection 151 (2021) 244–256. https://doi.org/10.1016/j.psep.2021.05.022.

[103] Q. Bi, C. Zhang, J. Liu, X. Liu, S. Xu, Positively charged zwitterion-carbon nitride functionalized nanofiltration membranes with excellent separation performance of Mg2+/Li+ and good antifouling properties, Separation and Purification Technology 257 (2021). https://doi.org/10.1016/j.seppur.2020.117959.
[104] C. Guo, X. Qian, F. Tian, N. Li, W. Wang, Z. Xu, S. Zhang, Amino-rich carbon quantum dots ultrathin nanofiltration membranes by double “one-step” methods: Breaking through trade-off among separation, permeation and stability, Chemical Engineering Journal 404 (2021). https://doi.org/10.1016/j.cej.2020.127144.
[105] D. Lu, T. Ma, S. Lin, Z. Zhou, G. Li, Q. An, Z. Yao, Q. Sun, Z. Sun, L. Zhang, Constructing a selective blocked-nanolayer on nanofiltration membrane via surface-charge inversion for promoting Li+ permselectivity over Mg2+, Journal of Membrane Science 635 (2021). https://doi.org/10.1016/j.memsci.2021.119504.
[106] M.G. Shin, J.Y. Seo, H. Park, Y.-I. Park, S. Ji, S.S. Lee, J.-H. Lee, Positively charged membranes with fine-tuned nanopores for ultrafast and high-precision cation separation, Journal of Materials Chemistry A 9 (2021) 24355–24364. https://doi.org/10.1039/d1ta07865h.
[107] L. Wang, D. Rehman, P.-F. Sun, A. Deshmukh, L. Zhang, Q. Han, Z. Yang, Z. Wang, H.-D. Park, J.H. Lienhard, C.Y. Tang, Novel Positively Charged Metal-Coordinated Nanofiltration Membrane for Lithium Recovery, ACS Applied Materials and Interfaces 13 (2021) 16906–16915. https://doi.org/10.1021/acsami.1c02252.
[108] P. Xu, J. Hong, Z. Xu, H. Xia, Q.-Q. Ni, MWCNTs-COOK-assisted high positively charged composite membrane: Accelerating Li+ enrichment and Mg2+ removal, Composites Part B: Engineering 212 (2021). https://doi.org/10.1016/j.compositesb.2021.108686.
[109] P. Xu, J. Hong, Z. Xu, H. Xia, Q.-Q. Ni, Novel aminated graphene quantum dots (GQDs-NH2)-engineered nanofiltration membrane with high Mg2+/Li+ separation efficiency, Separation and Purification Technology 258 (2021). https://doi.org/10.1016/j.seppur.2020.118042.
[110] P. Xu, J. Hong, Z. Xu, H. Xia, Q.-Q. Ni, Positively charged nanofiltration membrane based on (MWCNTs-COOK)-engineered substrate for fast and efficient lithium extraction, Separation and Purification Technology 270 (2021). https://doi.org/10.1016/j.seppur.2021.118796.
[111] Z. Yang, W. Fang, Z. Wang, R. Zhang, Y. Zhu, J. Jin, Dual-skin layer nanofiltration membranes for highly selective Li+/Mg2+ separation, Journal of Membrane Science 620 (2021). https://doi.org/10.1016/j.memsci.2020.118862.
[112] M.A. Ashraf, J. Wang, B. Wu, P. Cui, B. Xu, X. Li, Enhancement in Li+/Mg2+ separation from salt lake brine with PDA–PEI composite nanofiltration membrane, Journal of Applied Polymer Science 137 (2020). https://doi.org/10.1002/app.49549.
[113] L. Gao, H. Wang, Y. Zhao, M. Wang, The Application of Nanofiltration for Separating Aluminium and Lithium from Lepidolite Leaching Solution, ChemistrySelect 5 (2020) 4979–4987. https://doi.org/10.1002/slct.202000274.
[114] C. Guo, N. Li, X. Qian, J. Shi, M. Jing, K. Teng, Z. Xu, Ultra-thin double Janus nanofiltration membrane for separation of Li+ and Mg2+: “Drag” effect from carboxyl-containing negative interlayer, Separation and Purification Technology 230 (2020). https://doi.org/10.1016/j.seppur.2019.05.009.
[115] H. Wu, Y. Lin, W. Feng, T. Liu, L. Wang, H. Yao, X. Wang, A novel nanofiltration membrane with [MimAP][Tf2N] ionic liquid for utilization of lithium from brines with high Mg2+/Li+ ratio, Journal of Membrane Science 603 (2020). https://doi.org/10.1016/j.memsci.2020.117997.

[116] X. Wu, H. Liu, Y. Wei, Y. Fei, H. Qi, Negatively charged organic–inorganic hybrid silica nanofiltration membranes for lithium extraction, Chinese Journal of Chemical Engineering 28 (2020) 749–757. https://doi.org/10.1016/j.cjche.2019.11.004.
[117] P. Xu, J. Hong, X. Qian, Z. Xu, H. Xia, Q.-Q. Ni, "Bridge" graphene oxide modified positive charged nanofiltration thin membrane with high efficiency for Mg2+/Li+ separation, Desalination 488 (2020). https://doi.org/10.1016/j.desal.2020.114522.
[118] Q. Shen, S.-J. Xu, Z.-L. Xu, H.-Z. Zhang, Z.-Q. Dong, Novel thin-film nanocomposite membrane with water-soluble polyhydroxylated fullerene for the separation of Mg2+/Li+ aqueous solution, Journal of Applied Polymer Science 136 (2019). https://doi.org/10.1002/app.48029.
[119] P. Xu, W. Wang, X. Qian, H. Wang, C. Guo, N. Li, Z. Xu, K. Teng, Z. Wang, Positive charged PEI-TMC composite nanofiltration membrane for separation of Li+ and Mg2+ from brine with high Mg2+/Li+ ratio, Desalination 449 (2019) 57–68. https://doi.org/10.1016/j.desal.2018.10.019.
[120] X. Cui, G. Kong, S. Wei, Y. Cui, P. Yu, Z. Kang, H. Guo, Amino-grafted MOF-based composite membranes for improving Li+/Mg2+ separation performance, Separation and Purification Technology 330 (2024) 125485. https://doi.org/10.1016/j.seppur.2023.125485.
[121] J. Zhao, R. Fan, S. Xiang, J. Hu, X. Zheng, Preparation and Lithium-Ion Separation Property of ZIF-8 Membrane with Excellent Flexibility, Membranes 13 (2023) 500. https://doi.org/10.3390/membranes13050500.
[122] X. Zeng, L. Xu, T. Deng, Y. Wang, W. Xu, W. Zhang, Anionic MOFs Embedded in Anion-Exchange Membranes for the Separation of Lithium/Magnesium Cations, ACS Sustainable Chem. Eng. 11 (2023) 12877–12887. https://doi.org/10.1021/acssuschemeng.3c00891.
[123] S. Pang, L. Dai, Z. Yi, K. Qu, Y. Wang, Y. Wu, C. Fang, K. Huang, Z. Xu, 2D nanofluidic vermiculite membranes with self-confinement channels and recognition sites for ultrafast lithium ion-selective transport, Journal of Membrane Science 687 (2023) 122054. https://doi.org/10.1016/j.memsci.2023.122054.
[124] J. Hua, J. He, H. Pei, X. Ma, S.R. Wickramasinghe, J. Li, Supported ionic liquid membrane contactor with crown ether functionalized polyimide membrane for high-efficient Li+/Mg2+ selective separation, Journal of Membrane Science 687 (2023) 122038. https://doi.org/10.1016/j.memsci.2023.122038.
[125] Y. Lu, R. Zhou, N. Wang, Y. Yang, Z. Zheng, M. Zhang, Q.-F. An, J. Yuan, Engineer Nanoscale Defects into Selective Channels: MOF-Enhanced Li+ Separation by Porous Layered Double Hydroxide Membrane, Nano-Micro Lett. 15 (2023) 147. https://doi.org/10.1007/s40820-023-01101-w.
[126] Q. Huang, S. Liu, Y. Guo, G. Liu, W. Jin, Separation of mono-/di-valent ions via charged interlayer channels of graphene oxide membranes, Journal of Membrane Science 645 (2022). https://doi.org/10.1016/j.memsci.2021.120212.
[127] L. Xu, X. Zeng, Q. He, T. Deng, C. Zhang, W. Zhang, Stable ionic liquid-based polymer inclusion membranes for lithium and magnesium separation, Separation and Purification Technology 288 (2022). https://doi.org/10.1016/j.seppur.2022.120626.
[128] X. Zeng, L. Xu, T. Deng, C. Zhang, W. Xu, W. Zhang, Polymer Inclusion Membranes with P507-TBP Carriers for Lithium Extraction from Brines, Membranes 12 (2022). https://doi.org/10.3390/membranes12090839.
[129] W. Zhou, Z. Li, Q. Bi, L. Ma, S. Xu, Extraction of Lithium from Brine with a High Mg/Li Ratio Using Polymer Inclusion Membrane Containing Tri-n-butyl Phosphate and Ionic Liquid, Journal of Sustainable Metallurgy 8 (2022) 1639–1649. https://doi.org/10.1007/s40831-022-00592-y.
[130] Z. Lu, Y. Wu, L. Ding, Y. Wei, H. Wang, A Lamellar MXene (Ti3C2Tx)/PSS Composite Membrane for Fast and Selective Lithium-Ion Separation, Angewandte Chemie - International Edition 60 (2021) 22265–22269. https://doi.org/10.1002/anie.202108801.

[131] F. Sheng, B. Wu, X. Li, T. Xu, M.A. Shehzad, X. Wang, L. Ge, H. Wang, T. Xu, Efficient Ion Sieving in Covalent Organic Framework Membranes with Sub-2-Nanometer Channels, Advanced Materials 33 (2021). https://doi.org/10.1002/adma.202104404.
[132] C. Zhang, Y. Mu, S. Zhao, W. Zhang, Y. Wang, Lithium extraction from synthetic brine with high Mg2+/Li+ ratio using the polymer inclusion membrane, Desalination 496 (2020). https://doi.org/10.1016/j.desal.2020.114710.
[133] J. Zhang, X. Cui, F. Yang, L. Qu, F. Du, H. Zhang, J. Wang, Hybrid Cation Exchange Membranes with Lithium Ion-Sieves for Highly Enhanced Li+ Permeation and Permselectivity, Macromolecular Materials and Engineering 304 (2019). https://doi.org/10.1002/mame.201800567.
[134] J. Lu, C. Dai, S. Li, D. Zou, Y. Sun, W. Jing, Ultraefficient Li+/Mg2+ separation with MXene/CNT membranes under electric field assistance, Separation and Purification Technology 338 (2024) 126508. https://doi.org/10.1016/j.seppur.2024.126508.
[135] J. Li, Y. Shi, C. Qi, B. Zhang, X. Xing, Y. Li, T. Chen, X. Mao, Z. Zuo, X. Zhao, Z. Pan, L. Li, X. Yang, C. Li, Charging Metal-Organic Framework Membranes by Incorporating Crown Ethers to Capture Cations for Ion Sieving, Angewandte Chemie International Edition 62 (2023) e202309918. https://doi.org/10.1002/anie.202309918.
[136] L. Fu, Q. Wang, Y. Hu, Y. Qian, W. Xin, S. Zhou, X.-Y. Kong, L. Wen, Construction of a hierarchical membrane with angstrom-scale ion channels for enhanced Li+/Mg2+ separation, Chem. Commun. 59 (2023) 9384–9387. https://doi.org/10.1039/D3CC00777D.
[137] B. Li, J. Peng, M. Li, Z. Yang, J. Lu, J. Han, Facile synthesis and exfoliation of micro-sized LDH to fabricate 2D membranes towards Mg/Li separation, AIChE Journal 69 (2023) e18212. https://doi.org/10.1002/aic.18212.
[138] T. Wen, Y. Zhao, X. Wang, L. Chen, R. Gao, S. Wang, T. Zhang, Efficient and ultrafast separation of Li+ and Mg2+ by the porous two-dimensional nanochannel of perm-selective montmorillonite membrane, Chemical Engineering Journal 475 (2023) 146101. https://doi.org/10.1016/j.cej.2023.146101.
[139] L. Fu, Y. Teng, P. Liu, W. Xin, Y. Qian, L. Yang, X. Lin, Y. Hu, X.-Y. Kong, L. Jiang, L. Wen, Electrochemical ion-pumping-assisted transfer system featuring a heterogeneous membrane for lithium recovery, Chemical Engineering Journal 435 (2022). https://doi.org/10.1016/j.cej.2022.134955.
[140] R. Xu, Y. Kang, W. Zhang, X. Zhang, B. Pan, Oriented UiO-67 Metal–Organic Framework Membrane with Fast and Selective Lithium-Ion Transport, Angewandte Chemie - International Edition 61 (2022). https://doi.org/10.1002/anie.202115443.
[141] T. Zhang, H. Bai, Y. Zhao, B. Ren, T. Wen, L. Chen, S. Song, S. Komarneni, Precise Cation Recognition in Two-Dimensional Nanofluidic Channels of Clay Membranes Imparted from Intrinsic Selectivity of Clays, ACS Nano 16 (2022) 4930–4939. https://doi.org/10.1021/acsnano.2c00866.
[142] Z. Zhou, D.B. Shinde, D. Guo, L. Cao, R.A. Nuaimi, Y. Zhang, L.R. Enakonda, Z. Lai, Flexible Ionic Conjugated Microporous Polymer Membranes for Fast and Selective Ion Transport, Advanced Functional Materials 32 (2022). https://doi.org/10.1002/adfm.202108672.
[143] H. Ahmadi, E. Hosseini, W. Cha-Umpong, M. Abdollahzadeh, A.H. Korayem, A. Razmjou, V. Chen, M. Asadnia, Incorporation of Natural Lithium-Ion Trappers into Graphene Oxide Nanosheets, Advanced Materials Technologies 6 (2021). https://doi.org/10.1002/admt.202000665.
[144] H.M. Saif, R.M. Huertas, S. Pawlowski, J.G. Crespo, S. Velizarov, Development of highly selective composite polymeric membranes for Li+/Mg2+ separation, Journal of Membrane Science 620 (2021). https://doi.org/10.1016/j.memsci.2020.118891.

[145] W. Xin, C. Lin, L. Fu, X.-Y. Kong, L. Yang, Y. Qian, C. Zhu, Q. Zhang, L. Jiang, L. Wen, Nacre-like Mechanically Robust Heterojunction for Lithium-Ion Extraction, Matter 4 (2021) 737–754. https://doi.org/10.1016/j.matt.2020.12.003.
[146] Z. Yuan, Y. Yu, L. Wei, C. Wang, X. Zhong, X. Sui, Z. Yu, D.S. Han, H. Shon, Y. Chen, Thermo-osmosis-Coupled Thermally Regenerative Electrochemical Cycle for Efficient Lithium Extraction, ACS Applied Materials and Interfaces 13 (2021) 6276–6285. https://doi.org/10.1021/acsami.0c20464.
[147] T. Xu, M.A. Shehzad, D. Yu, Q. Li, B. Wu, X. Ren, L. Ge, T. Xu, Highly Cation Permselective Metal–Organic Framework Membranes with Leaf-Like Morphology, ChemSusChem 12 (2019) 2593–2597. https://doi.org/10.1002/cssc.201900706.
[148] Y. Guo, Y. Ying, Y. Mao, X. Peng, B. Chen, Polystyrene Sulfonate Threaded through a Metal–Organic Framework Membrane for Fast and Selective Lithium-Ion Separation, Angewandte Chemie - International Edition 55 (2016) 15120–15124. https://doi.org/10.1002/anie.201607329.
[149] T. Wen, Y. Zhao, B. Kuang, Y. Sun, Y. Li, H. Wang, L. Chen, R. Gao, L. Zhang, T. Zhang, Enhanced Li+/Mg2+ selectivity of two-dimensional montmorillonite membranes by end-face and interfacial layer constraint for nanosheets, Separation and Purification Technology 339 (2024) 126707. https://doi.org/10.1016/j.seppur.2024.126707.
[150] W. Wang, C. Wang, Y. Zhang, H. Xu, L. Shao, Highly positively-charged membrane enabled by a competitive reaction for efficient Li+/Mg2+ separation, Separation and Purification Technology 330 (2024) 125428. https://doi.org/10.1016/j.seppur.2023.125428.
[151] Y. Xu, Q. Chen, Y. Gao, J. Wang, H. Fan, F. Zhao, Performance comparison of lithium fractionation from magnesium *via* continuous selective nanofiltration/electrodialysis, Chinese Journal of Chemical Engineering 59 (2023) 42–50. https://doi.org/10.1016/j.cjche.2022.11.013.
[152] L. Dai, S. Pang, S. Li, Z. Yi, K. Qu, Y. Wang, Y. Wu, S. Li, L. Lei, K. Huang, X. Guo, Z. Xu, Freestanding two-dimensional nanofluidic membranes modulated by zwitterionic polyelectrolyte for mono-/di-valent ions selectivity transport, Journal of Membrane Science 677 (2023) 121621. https://doi.org/10.1016/j.memsci.2023.121621.
[153] W.J. Chang, S. Jang, M. Kim, Y. Kim, D.Y. Jeong, B. Kim, J.M. Kim, S. Nam, W.I. Park, MoS2 Passivated Multilayer Graphene Membranes for Li-Ion Extraction From Seawater, Small 19 (2023) 2207020. https://doi.org/10.1002/smll.202207020.
[154] J. Chen, J. Wang, Z.-Y. Ji, Z. Guo, P. Zhang, Z. Huang, Electro-nanofiltration membranes with high Li+/Mg2+ selectivity prepared via sequential interfacial polymerization, Desalination 549 (2023) 116312. https://doi.org/10.1016/j.desal.2022.116312.
[155] Y. Dong, Y. Liu, H. Li, Q. Zhu, M. Luo, H. Zhang, B. Ye, Z. Yang, T. Xu, Crown ether-based Tröger's base membranes for efficient Li+/Mg2+ separation, Journal of Membrane Science 665 (2023). https://doi.org/10.1016/j.memsci.2022.121113.
[156] M. He, Z. Liu, L. Wang, J. Zhu, J. Wang, R. Miao, Y. Lv, X. Wang, Carboxymethylcellulose (CMC)/glutaraldehyde (GA)-modified Ti3C2Tx membrane and its efficient ion sieving performance, Journal of Membrane Science 675 (2023) 121541. https://doi.org/10.1016/j.memsci.2023.121541.
[157] H. Liu, Y. Liu, Y. Lei, Y. Zhou, N. Ma, J. Xue, Y. Kuang, S. Chen, G. Hong, L. Cao, Latent track PET membranes for high-performance Li+/Mg2+ selectivity assisted with trivalent ion, Nuclear Instruments and Methods in Physics Research, Section B: Beam Interactions with Materials and Atoms 536 (2023) 23–29. https://doi.org/10.1016/j.nimb.2022.12.019.
[158] X. Ruan, C. Zhang, Y. Zhu, F. Cai, Y. Yang, J. Feng, X. Ma, Y. Zheng, H. Li, Y. Yuan, G. Zhu, Constructing Mechanical Shuttles in a Three-dimensional (3D) Porous Architecture for Selective Transport of Lithium Ions, Angewandte Chemie - International Edition 62 (2023). https://doi.org/10.1002/anie.202216549.

[159] S. Wang, L. Zhu, R. Yang, M. Li, F. Dai, S. Sheng, L. Chen, S. Liang, Insights into High Li+/Mg2+ Separation Performance Using a PEI-Grafted Graphene Oxide Membrane, Journal of Physical Chemistry C (2023). https://doi.org/10.1021/acs.jpcc.3c00723.
[160] W. Wang, G. Hong, Y. Zhang, X. Yang, N. Hu, J. Zhang, P. Sorokin, L. Shao, Designing an energy-efficient multi-stage selective electrodialysis process based on high-performance materials for lithium extraction, Journal of Membrane Science 675 (2023). https://doi.org/10.1016/j.memsci.2023.121534.
[161] L. Tao, X. Wang, F. Wu, B. Wang, C. Gao, X. Gao, Highly efficient Li+/Mg2+ separation of monovalent cation permselective membrane enhanced by 2D metal organic framework nanosheets, Separation and Purification Technology 296 (2022). https://doi.org/10.1016/j.seppur.2022.121309.
[162] W. Wang, J. Sun, Y. Zhang, Y. Zhang, G. Hong, R.M. Moutloali, B.B. Mamba, F. Li, J. Ma, L. Shao, Mussel-inspired tannic acid/polyethyleneimine assembling positively-charged membranes with excellent cation permselectivity, Science of the Total Environment 817 (2022). https://doi.org/10.1016/j.scitotenv.2022.153051.
[163] W. Wang, Y. Zhang, F. Li, Y. Chen, S.M. Mojallali Rostami, S.S. Hosseini, L. Shao, Mussel-inspired polyphenol/polyethyleneimine assembled membranes with highly positive charged surface for unprecedented high cation perm-selectivity, Journal of Membrane Science 658 (2022). https://doi.org/10.1016/j.memsci.2022.120703.
[164] W. Wang, Y. Zhang, X. Yang, H. Sun, Y. Wu, L. Shao, Monovalent Cation Exchange Membranes with Janus Charged Structure for Ion Separation, Engineering (2022). https://doi.org/10.1016/j.eng.2021.09.020.
[165] T. Xu, B. Wu, L. Hou, Y. Zhu, F. Sheng, Z. Zhao, Y. Dong, J. Liu, B. Ye, X. Li, L. Ge, H. Wang, T. Xu, Highly Ion-Permselective Porous Organic Cage Membranes with Hierarchical Channels, Journal of the American Chemical Society 144 (2022) 10220–10229. https://doi.org/10.1021/jacs.2c00318.
[166] J. Ying, Y. Lin, Y. Zhang, Y. Jin, H. Matsuyama, J. Yu, Layer-by-layer assembly of cation exchange membrane for highly efficient monovalent ion selectivity, Chemical Engineering Journal 446 (2022). https://doi.org/10.1016/j.cej.2022.137076.
[167] L. Hou, W. Xian, S. Bing, Y. Song, Q. Sun, L. Zhang, S. Ma, Understanding the Ion Transport Behavior across Nanofluidic Membranes in Response to the Charge Variations, Advanced Functional Materials 31 (2021). https://doi.org/10.1002/adfm.202009970.
[168] X. Pang, X. Yu, Y. He, S. Dong, X. Zhao, J. Pan, R. Zhang, L. Liu, Preparation of monovalent cation perm-selective membranes by controlling surface hydration energy barrier, Separation and Purification Technology 270 (2021). https://doi.org/10.1016/j.seppur.2021.118768.
[169] N. Ul Afsar, X. Ge, Z. Zhao, A. Hussain, Y. He, L. Ge, T. Xu, Zwitterion membranes for selective cation separation via electrodialysis, Separation and Purification Technology 254 (2021). https://doi.org/10.1016/j.seppur.2020.117619.
[170] J. Zhou, Z. Jiao, Q. Zhu, Y. Li, L. Ge, L. Wu, Z. Yang, T. Xu, Biselective microporous Tröger’s base membrane for effective ion separation, Journal of Membrane Science 627 (2021). https://doi.org/10.1016/j.memsci.2021.119246.
[171] X. Pang, Y. Tao, Y. Xu, J. Pan, J. Shen, C. Gao, Enhanced monovalent selectivity of cation exchange membranes via adjustable charge density on functional layers, Journal of Membrane Science 595 (2020). https://doi.org/10.1016/j.memsci.2019.117544.
[172] P.P. Sharma, V. Yadav, A. Rajput, H. Gupta, H. Saravaia, V. Kulshrestha, Sulfonated poly (ether ether ketone) composite cation exchange membrane for selective recovery of lithium by electrodialysis, Desalination 496 (2020). https://doi.org/10.1016/j.desal.2020.114755.

[173] F. Sheng, N.U. Afsar, Y. Zhu, L. Ge, T. Xu, PVA-based mixed matrix membranes comprising ZSM-5 for cations separation, Membranes 10 (2020) 1–16. https://doi.org/10.3390/membranes10060114.
[174] F. Sheng, L. Hou, X. Wang, M. Irfan, M.A. Shehzad, B. Wu, X. Ren, L. Ge, T. Xu, Electro-nanofiltration membranes with positively charged polyamide layer for cations separation, Journal of Membrane Science 594 (2020). https://doi.org/10.1016/j.memsci.2019.117453.
[175] T. Xu, F. Sheng, B. Wu, M.A. Shehzad, A. Yasmin, X. Wang, Y. He, L. Ge, X. Zheng, T. Xu, Ti-exchanged UiO-66-NH2–containing polyamide membranes with remarkable cation permselectivity, Journal of Membrane Science 615 (2020). https://doi.org/10.1016/j.memsci.2020.118608.
[176] N.U. Afsar, W. Ji, B. Wu, M.A. Shehzad, L. Ge, T. Xu, SPPO-based cation exchange membranes with a positively charged layer for cation fractionation, Desalination 472 (2019). https://doi.org/10.1016/j.desal.2019.114145.
[177] N.U. Afsar, M.A. Shehzad, M. Irfan, K. Emmanuel, F. Sheng, T. Xu, X. Ren, L. Ge, T. Xu, Cation exchange membrane integrated with cationic and anionic layers for selective ion separation via electrodialysis, Desalination 458 (2019) 25–33. https://doi.org/10.1016/j.desal.2019.02.004.
[178] M. Irfan, T. Xu, L. Ge, Y. Wang, T. Xu, Zwitterion structure membrane provides high monovalent/divalent cation electrodialysis selectivity: Investigating the effect of functional groups and operating parameters, Journal of Membrane Science 588 (2019). https://doi.org/10.1016/j.memsci.2019.117211.
[179] D. Sun, M. Meng, Y. Yin, Y. Zhu, H. Li, Y. Yan, Highly selective, regenerated ion-sieve microfiltration porous membrane for targeted separation of Li+, Journal of Porous Materials 23 (2016) 1411–1419. https://doi.org/10.1007/s10934-016-0201-4.
[180] L. Wang, L. Wang, L. Li, Preparation of PVC-LMZO membrane and its lithium adsorption performance from brine, Desalination 561 (2023) 116689. https://doi.org/10.1016/j.desal.2023.116689.
[181] G. He, Z. Li, Y. Liu, M. Liu, C. Zhu, L. Zhang, H. Zhang, A novel lithium ion-imprinted membrane with robust adsorption capacity and anti-fouling property based on the uniform multilayered interlayer, Desalination 539 (2022). https://doi.org/10.1016/j.desal.2022.115973.
[182] Z. Qiu, M. Wang, Y. Chen, T. Zhang, D. Yang, F. Qiu, Li4Mn5O12 doped cellulose acetate membrane with low Mn loss and high stability for enhancing lithium extraction from seawater, Desalination 506 (2021). https://doi.org/10.1016/j.desal.2021.115003.
[183] Z. Wang, X. Li, B. Tao, R. Guo, Preparation of Sulfonated Polyarylene Ether Nitrile Hollow Fiber Membrane Adsorbent and Its Potential in Separation Lithium Ion from Brine, ChemistrySelect 6 (2021) 6652–6660. https://doi.org/10.1002/slct.202101516.
[184] Z.-Y. Guo, Z.-Y. Ji, H.-Y. Chen, J. Liu, Y.-Y. Zhao, F. Li, J.-S. Yuan, Effect of Impurity Ions in the Electrosorption Lithium Extraction Process: Generation and Restriction of "Selective Concentration Polarization," ACS Sustainable Chemistry and Engineering 8 (2020) 11834–11844. https://doi.org/10.1021/acssuschemeng.0c04359.
[185] J. Cui, Z. Zhou, A. Xie, S. Liu, Q. Wang, Y. Wu, Y. Yan, C. Li, Facile synthesis of degradable CA/CS imprinted membrane by hydrolysis polymerization for effective separation and recovery of Li+, Carbohydrate Polymers 205 (2019) 492–499. https://doi.org/10.1016/j.carbpol.2018.10.094.
[186] L. Mao, R. Chen, J. He, H. Pei, B. He, X. Ma, J. Li, Remarkably High Li+Adsorptive Separation Polyamide Membrane by Improving the Crown Ether Concentration and Electron Density, ACS Sustainable Chemistry and Engineering 10 (2022) 10047–10056. https://doi.org/10.1021/acssuschemeng.2c02950.

[187] J. Yang, G. Qu, C. Liu, S. Zhou, B. Li, Y. Wei, An effective lithium ion-imprinted membrane containing 12-crown ether-4 for selective recovery of lithium, Chemical Engineering Research and Design 184 (2022) 639–650. https://doi.org/10.1016/j.cherd.2022.06.039.
[188] Q. Cheng, Y. Zhang, X. Zheng, W. Sun, B. Li, D. Wang, Z. Li, High specific surface crown ether modified chitosan nanofiber membrane by low-temperature phase separation for efficient selective adsorption of lithium, Separation and Purification Technology 262 (2021). https://doi.org/10.1016/j.seppur.2021.118312.
[189] W. Liu, G. Yan, E. Zhang, Q. Liang, L. Qin, M. Wang, X. Liu, Y. Yang, Extraction of lithium ions from acidic solution using electrochemically imprinted membrane, Desalination 496 (2020). https://doi.org/10.1016/j.desal.2020.114751.
[190] Q. Zhu, X. Ma, H. Pei, J. Li, F. Yan, Z. Cui, H. Wang, J. Li, A highly-efficient lithium adsorptive separation membrane derived from a polyimide-containing dibenzo-14-crown-4 moiety, Separation and Purification Technology 247 (2020). https://doi.org/10.1016/j.seppur.2020.116940.
[191] D. Sun, M. Meng, Y. Lu, B. Hu, Y. Yan, C. Li, Porous nanocomposite membranes based on functional GO with selective function for lithium adsorption, New Journal of Chemistry 42 (2018) 4432–4442. https://doi.org/10.1039/c7nj04733a.
[192] D. Sun, M. Meng, Y. Qiao, Y. Zhao, Y. Yan, C. Li, Synthesis of ion imprinted nanocomposite membranes for selective adsorption of lithium, Separation and Purification Technology 194 (2018) 64–72. https://doi.org/10.1016/j.seppur.2017.10.052.
[193] D. Sun, Y. Zhu, M. Meng, Y. Qiao, Y. Yan, C. Li, Fabrication of highly selective ion imprinted macroporous membranes with crown ether for targeted separation of lithium ion, Separation and Purification Technology 175 (2017) 19–26. https://doi.org/10.1016/j.seppur.2016.11.029.
[194] L. Huang, H. Wu, L. Ding, J. Caro, H. Wang, Shearing Liquid-Crystalline MXene into Lamellar Membranes with Super-Aligned Nanochannels for Ion Sieving, Angewandte Chemie International Edition 63 (2024) e202314638. https://doi.org/10.1002/anie.202314638.
[195] H. Ma, Y. Xia, Z. Wang, T. Xu, G.P. Simon, H. Wang, Dual-Channel-Ion Conductor Membrane for Low-Energy Lithium Extraction, Environ. Sci. Technol. 57 (2023) 17246–17255. https://doi.org/10.1021/acs.est.3c05935.
[196] T. Ounissi, L. Dammak, J.-F. Fauvarque, E. Selmane Bel Hadj Hmida, Ecofriendly lithium-sodium separation by diffusion processes using lithium composite membrane, Separation and Purification Technology 275 (2021). https://doi.org/10.1016/j.seppur.2021.119134.
[197] T. Ounissi, L. Dammak, C. Larchet, J.-F. Fauvarque, E. Selmane Bel Hadj Hmida, Novel lithium selective composite membranes: synthesis, characterization and validation tests in dialysis, Journal of Materials Science 55 (2020) 16111–16128. https://doi.org/10.1007/s10853-020-05147-8.
[198] C. Cai, F. Yang, Z. Zhao, Q. Liao, R. Bai, W. Guo, P. Chen, Y. Zhang, H. Zhang, Promising transport and high-selective separation of Li(I) from Na(I) and K(I) by a functional polymer inclusion membrane (PIM) system, Journal of Membrane Science 579 (2019) 1–10. https://doi.org/10.1016/j.memsci.2019.02.046.
[199] T. Harit, F. Malek, Elaboration of new thin solid membrane bearing a tetrapyrazolic macrocycle for the selective transport of lithium cation, Separation and Purification Technology 188 (2017) 394–398. https://doi.org/10.1016/j.seppur.2017.07.060.
[200] X. Tong, S. Liu, Y. Zhao, L. Huang, J. Crittenden, Y. Chen, MXene Composite Membranes with Enhanced Ion Transport and Regulated Ion Selectivity, Environmental Science and Technology 56 (2022) 8964–8974. https://doi.org/10.1021/acs.est.2c01765.

[201] X. Meng, Y. Long, Y. Tian, W. Li, T. Liu, S. Huo, Electro-membrane extraction of lithium with D2EHPA/TBP compound extractant, Hydrometallurgy 202 (2021). https://doi.org/10.1016/j.hydromet.2021.105615.
[202] S. Liu, X. Tong, L. Huang, C. Xiao, K. Zhang, Y. Chen, J. Crittenden, Lithium-ion extraction using electro-driven freestanding graphene oxide composite membranes, Journal of Membrane Science 672 (2023). https://doi.org/10.1016/j.memsci.2023.121448.
[203] M. Bazrgar Bajestani, A. Moheb, M. Dinari, Preparation of lithium ion-selective cation exchange membrane for lithium recovery from sodium contaminated lithium bromide solution by electrodialysis process, Desalination 486 (2020). https://doi.org/10.1016/j.desal.2020.114476.
[204] Y. Sun, Y. Wang, Y. Liu, X. Xiang, Highly Efficient Lithium Extraction from Brine with a High Sodium Content by Adsorption-Coupled Electrochemical Technology, ACS Sustainable Chemistry and Engineering 9 (2021) 11022–11031. https://doi.org/10.1021/acssuschemeng.1c02442.
[205] F. Yang, L. Li, J. Hua, J. He, X. Ma, J. Li, Electrospun nanofiber membrane of dibenzo 14-crown-4-ether polyimide for efficient selective lithium recovery from discarded lithium-ion batteries, Separation and Purification Technology 334 (2024) 126018. https://doi.org/10.1016/j.seppur.2023.126018.
[206] H. Jo, T.-H. Le, H. Lee, J. Lee, M. Kim, S. Lee, M. Chang, H. Yoon, Macrocyclic ligand-embedded graphene-in-polymer nanofiber membranes for lithium ion recovery, Chemical Engineering Journal 452 (2023). https://doi.org/10.1016/j.cej.2022.139274.
[207] G. Qu, J. Yang, Y. Ran, B. Li, H. Wang, Y. Wei, Adsorption performance and mechanism of TiO2/PVDF-based lithium-ion imprinted membrane in leaching solution of spent lithium-ion batteries, Journal of Cleaner Production 442 (2024) 140982. https://doi.org/10.1016/j.jclepro.2024.140982.
